# Integrated Mental Healthcare and Vocational Rehabilitation for People on Sick Leave with Anxiety or Depression: 24-Month Follow-up of the Randomized IBBIS Trial

**DOI:** 10.1007/s10926-023-10094-7

**Published:** 2023-02-27

**Authors:** Andreas Hoff, Rie Mandrup Poulsen, Jonas Peter Fisker, Carsten Hjorthøj, Merete Nordentoft, Ulla Christensen, Anders Bo Bojesen, Lene Falgaard Eplov

**Affiliations:** 1 Copenhagen Research Centre for Mental Health – CORE, Mental Health Centre Copenhagen, Copenhagen University Hospital, Gentofte Hospitalsvej 15, Hellerup, 2900 Denmark; 2 The National Board of Social Services in Denmark, Edisonsvej 1, Odense, 5000 Denmark; 3 Hejmdal Private hospital, Martinsvej 7-9, Frederiksberg C, 1926 Denmark; 4 Department of Public Health, Section of Epidemiology, University of Copenhagen, Copenhagen, Denmark; 5 Department of Public Health, Faculty of Health and Medical Science, University of Copenhagen, Øster Farimagsgade 5, P.O.B. 2099, Copenhagen K, 1014 Denmark

**Keywords:** Depression, Anxiety, Return to work, Integrated services, Mental healthcare, Cognitive Behavioural Therapy, Vocational rehabilitation, RCT, Common Mental Disorders

## Abstract

Integration of vocational rehabilitation and mental healthcare has shown some effect on work participation at 1-year follow-up after sick leave with depression and anxiety. We aimed to study the effect on work and health outcomes at 2-year follow-up, why we performed a randomized trial was conducted to study the effectiveness of integrated intervention (INT) compared to service as usual (SAU) and best practice mental healthcare (MHC). We included 631 participants, and at 24-month follow-up, we detected no differences in effect between INT and SAU. Compared to MHC, INT showed faster return-to-work (RTW) rates (p = 0.044) and a higher number of weeks in work (p = 0.024). No symptom differences were observed between the groups at 24 months. In conclusion, compared to SAU, INT was associated with a slightly higher work rate reaching borderline statistical significance at 12-month follow-up and lower stress levels at 6-month follow-up. The disappearance of relative effect between 12 and 24 months may be explained by the fact that the intervention lasted less than 12 months or by delayed spontaneous remission in the SAU group after 12 months. Despite the lack of effect at long-term follow-up, INT still performed slightly better than SAU overall. Moderate implementation difficulties, may partly explain the absence of the hypothesized effect. Integrated intervention, as implemented in this trial, showed some positive effects on mid-term vocational status and short-term stress symptom levels. However, these effects were not sustained beyond the duration of the intervention.

**Supplementary Information:**

The online version contains supplementary material available at 10.1007/s10926-023-10094-7.

## Introduction

Common mental disorders like anxiety and depression are highly prevalent [1] and associated with functional decline and sick leave [2]. They represent a substantial challenge for both individuals and society [3], not least since long-term sick leave is a risk factor for permanent exclusion from the labour market [4]. While unemployment is a causal risk factor for poor health and suicide [5], long-term sick leave entails a substantial financial burden in the form of lost productivity and benefit expenses [3, 6]. A review of RTW interventions from 2018 indicated that beneficial interventions often consist of several components [7], and some research suggests that *integration* of vocational rehabilitation and healthcare interventions may be beneficial [8]. The term integration can be defined as a “broad intersectoral system approach that aims to align the healthcare system […] with other human service systems” like vocational rehabilitation services [9]. However, integrated service models are difficult to implement [10], and only a few specific integrated service models such as the Norwegian At-Work-and-Coping (AWaC) intervention [11] have shown improved RTW for persons with common mental disorders (at medium-term follow-up (18 months)). In that study, the effect was sustained at 4-year follow-up for a subgroup on long-term benefits at baseline but it was not statistically significant for the group as a whole [12]. Furthermore, different types of interventions may be needed for different groups of people. For example, work-focused cognitive behavioural therapy (CBT) has been shown to be more effective than regular CBT for people on sick leave with high RTW self-efficacy. [13].

Between 2016 and 2018, a novel, integrated mental healthcare and vocational rehabilitation intervention for persons on sick leave due to depression or anxiety, the *IBBIS Integrated Intervention* (INT), was tested in a randomized controlled trial in Denmark [14]. “IBBIS” is a Danish acronym translating to “*Integrated Healthcare and Vocational Rehabilitation for Sick Leave Benefit Recipients*”. This intervention was compared to two non-integrated interventions: *Service As Usual (SAU)* and *IBBIS Mental Healthcare (MHC)*. In the latter group, best practice healthcare was largely ensured by providing cognitive behavioural therapy (CBT) in due time. The primary outcome of this trial was time to stable RTW at 12-month follow-up. Other outcomes were vocational status, proportion in work and number of weeks in work at 12-month follow-up; symptom and functional level; self-efficacy; quality of life; and intervention satisfaction. At 12-month follow-up, INT was not associated with faster RTW but with a slightly larger proportion in work compared to SAU (56.2% vs. 45%, Odds Ratio 0.64 [98.3%CI: 0.39‒1.05], p = 0.029). The difference in proportion in work was statistically significant when INT was compared to MHC (56.2% vs. 43.7%, Odds Ratio 0.59 [98.3%CI: 0.36‒0.98], p = 0.012). Compared to SAU, INT showed minor benefits in terms of levels of self-reported stress, exhaustion disorder, depression and disease-related self-efficacy and substantial benefits in terms of satisfaction with services at 6-month follow-up. No differences were detected on these outcomes at 12-month follow-up [14]. Since effects were sustained for some groups at long-term follow-up in some studies, we decided to measure outcomes at 24-month follow-up in this trial too. Furthermore, the need for long-term follow-up was highlighted by the fact that more than 40% of the participants employed at baseline had still not returned to work at 12-month follow-up.

### Objectives

The aim of this trial was to test the effect of INT by comparing it to two other interventions, MHC and SAU. We hypothesized that INT would yield better results than both MHC and SAU on all outcomes at all follow-ups, and that MHC would yield better outcomes than SAU. This paper reports the outcomes observed at 24-month follow-up.

## Methods

INT was tested in a three-arm, randomized, multi-centre superiority trial [14, 15]. Supplement 1 shows alterations in methods after trial commencement. The study was registered at ClinicalTrials.org (ID: NCT02885519), where a detailed statistical analysis plan was published before analysis of 6- and 12-month follow-up. Researchers were blinded to group allocation during these analyses but not during analysis at 24-month follow-up. This study is reported according to the *Consolidated Standards of Reporting Trials* criteria (CONSORT) for randomized trials of non-pharmacological interventions [16]. Participants were included from April 2016 to April 2018. This trial ran parallel to a similarly designed trial targeting sickness absentees with stress-related disorders (ClinicalTrials.org ID: NCT02885519) [17], but all outcomes were analysed completely separately.

### Study Setting

This multi-centre study was conducted as a collaboration between the Mental Health Services of the Capital Region of Denmark and four municipalities in the capital region. Denmark is characterized as a welfare society with a comprehensive social security system [18] and a comprehensive, mostly free-of-charge healthcare system funded by state taxes and governed by regions [19]. In Denmark, mental healthcare for persons with common mental disorders, including depression and anxiety, is mostly offered through the general practitioner [20]. More severe cases of anxiety or depression is treated in hospital-based, regional mental healthcare centres. However, there is a growing market for private or workplace-funded therapeutic services offered outside the public healthcare sector. In Denmark, compensation for lost work capacity is governed by municipal jobcentres and consists of either reimbursement to the employer or a direct payment to the person on sick leave, if the person is unemployed. This compensatory system is administered by local jobcentre case managers and governed by the national sickness benefit legislation. A reform of this legislation was introduced between 2014 and 2016 and gradually implemented during the following years.

Potential study participants were referred by case managers in the municipal jobcentres to a central research team based in the Mental Health Centre Copenhagen [15]. Participants were then recruited for a mental health assessment the effect of which was tested in a separate study (paper in preparation). In conjunction with this assessment, eligible absentees were randomized if they gave written consent. The IBBIS services were offered by two teams: Team City (Copenhagen municipality) and Team North (Gentofte, Gladsaxe and Lyngby-Taarbæk municipalities). An elaborate overview of the organization of IBBIS teams in the four trial sites is presented in the previously published study design paper [21].

### Participants

Persons were deemed eligible for the trial if they were ≥ 18-years of age, had been on sick leave for ≥ 4 weeks (irrespective of employment status) and received sickness benefit. Also, they had to be diagnosed with either depression (F32-F33), generalized anxiety disorder (F41.1), social phobia (F40.1) or panic disorder (F41.0). They also had to be proficient in Danish and reside in one of the collaborating municipalities. Persons were ineligible if they were pregnant, deemed at moderate or high risk of suicide, had substance abuse disorder that would hinder psychotherapy, had an unstable general medical condition, showed signs of dementia or were unwilling to refrain from engaging in psychotherapy outside the study during treatment (if allocated to groups INT or MHC). Eligibility was evaluated by trained mental health professionals and supervised by a psychiatric specialist. The instruments that guided the assessment are described in Supplement 2. The intended number of participants was acquired, and the sample size and power calculations are described in the study design paper [15].

### Interventions

#### IBBIS Integrated Intervention – INT

Participants allocated to INT received IBBIS Mental Healthcare as well as IBBIS Vocational Rehabilitation, and these two intervention components were integrated. IBBIS Mental Healthcare was a stepped mental healthcare intervention in which *care managers* provided psychoeducation and CBT according to need and diagnosis. Care managers were trained nurses, occupational therapists, physiotherapists or social workers with at least one year of training in CBT and relevant clinical experience. In IBBIS Vocational Rehabilitation, participants received case management of the sickness benefit case and vocational support based on the principles of *Individual Placement and Support* [22] and the *Sharp-at-work* intervention [23]. This support was provided by *employment consultants* according to need and employment status.

Integration of these two intervention components was ensured through (a) co-location of care managers, employment consultants and their supervisors in interdisciplinary and intersectoral IBBIS teams; (b) at least one so-called *roundtable meeting* between the care manager, the employment consultant and the participant; (c) interdisciplinary supervision for team members; and (d) interdisciplinary training of care mangers and employment consultants.

The plan was to standardize the interventions through the use of manuals. Care managers and the supervisory team were formally employed in Mental Health Centre Copenhagen, and employment consultants were employed in the four municipal jobcentres. No formal eligibility criteria were applied for employment consultants.

#### IBBIS Mental Healthcare – MHC

Since we did not expect that best practice mental healthcare would be available through primary care, it was not provided to participants in the SAU group. Consequently, any difference in outcomes between INT and SAU could solely be due to insufficient primary sector mental healthcare. For that reason, INT was not only compared to SAU but also to IBBIS Mental Healthcare (the MHC group). Finally, the MHC group was also compared to the SAU group.

#### Service as Usual – SAU

In the control group, SAU, services were a priori unknown to the researchers but were investigated during the trial and described elsewhere [14]. In SAU, all services were provided outside of the study. Participants received standard mental healthcare and standard vocational rehabilitation. Self-assessed data showed that participants in the SAU group received about the same number of therapeutic sessions as in IBBIS mental healthcare (delivered in both the INT and MHC intervention groups). Much of this healthcare was delivered outside the public healthcare system. Furthermore, external evaluation showed that the standard vocational rehabilitation and case management of sickness benefit offered to the SAU group were affected by changes to the sickness benefit legislation that were gradually implemented during part of the trial period. The reform entailed greater use of eligibility assessments and a new benefit type with a lower fiscal level, and it fastened RTW in the short term [24].

#### Protocol Adherence

In the two intervention groups, INT and MHC, we sought to investigate and optimize adherence to manuals through standardized fidelity reviews that measured implementation degree on a predefined fidelity scale. IBBIS Mental Healthcare (delivered in INT and MHC) was implemented with high fidelity. In INT, IBBIS Vocational Rehabilitation and the integration of services were only implemented with fair fidelity [14]. A specific problem was that the rather essential intervention component *workplace involvement* was only effectuated in a few cases in the INT group.

### Outcomes

Data were obtained at baseline through multiple sources: researcher-administered semi-structured interviews, self-reported questionnaires and register-based data. At 24-month follow-up, data were obtained through self-reported questionnaires and register-based weekly data from baseline to follow-up. To capture the complex nature of the RTW process, several outcomes were used to describe intervention effects at 24-month follow-up: time to RTW (secondary outcome) and the pre-registered explorative outcomes of weeks in work and proportion in work. The number of recurrent sick leaves was a post-hoc exploratory outcome. Self-reported data were used to measure symptoms, level of functioning, quality of life, self-efficacy and presenteeism (being ill whilst working). All self-assessed outcomes at 24-month follow-up were pre-registered exploratory outcomes. These are described in Supplement 3.

### Statistical Analyses

The allocation ratio between the three trial arms was 1:1:1. The execution of the computer-generated randomization and allocation to intervention is described elsewhere [14]. Baseline characteristics are reported using means and SDs for numeric variables and numbers with percentages for categorical variables. Time-to-RTW outcomes were analysed using Cox-regression and proportion in work using logistic regression. Self-reported outcomes were analysed using linear mixed-effects models. A detailed statistical analysis plan is found in Supplement 4. We performed no parameterization on the pre-planned outcome *recurrent sick leave* but only described the number of RTW events and reccurring sick leaves in each group. This was decided before unblinding.

The outcome analysis was based on the intention-to-treat principles. For strategies of handling missing data, see Supplement 4. To compensate for multiple testing, we performed Bonferroni adjustment of acceptable Type 1 error risk-level and present confidence intervals and p-values accordingly. Therefore, p-values below 0.017 were considered statistically significant, and confidence was reported as 98.3% intervals. Post-hoc, we decided that p-values between 0.017 and the traditional, yet somewhat arbitrary, level of 0.05 would be presented as *borderline statistically significant*, since the Bonferroni method has been criticized for being too conservative [25] (for a discussion of relevant terminology, see for example [26]). Pre-planned analyses were performed for the following subgroups: diagnosis (anxiety; depression), employment status at baseline (employed; unemployed), IBBIS teams (Team City; Team North) and first vs. last half of included participants.

Sensitivity analyses (best/worst-case scenarios) on self-reported outcomes were conducted by imputing all missing data as the mean of the outcome variable ± 2 SD; sensitivity analyses concerning possible missing register data were performed by imputing all missing data as the best/worst outcome. All analyses were performed using R statistical software.

### Blinding

Assessors at baseline and researchers performing statistical analyses up to 12-month follow-up were blinded to allocation. None of the analyses for this publication were performed blinded. Furthermore, no service users were blinded to allocation. No service providers – including the case managers who determined eligibility for sickness benefit (resulting in DREAM register data) after randomization – were blinded to allocation.

## Results

**Fig. 1:**
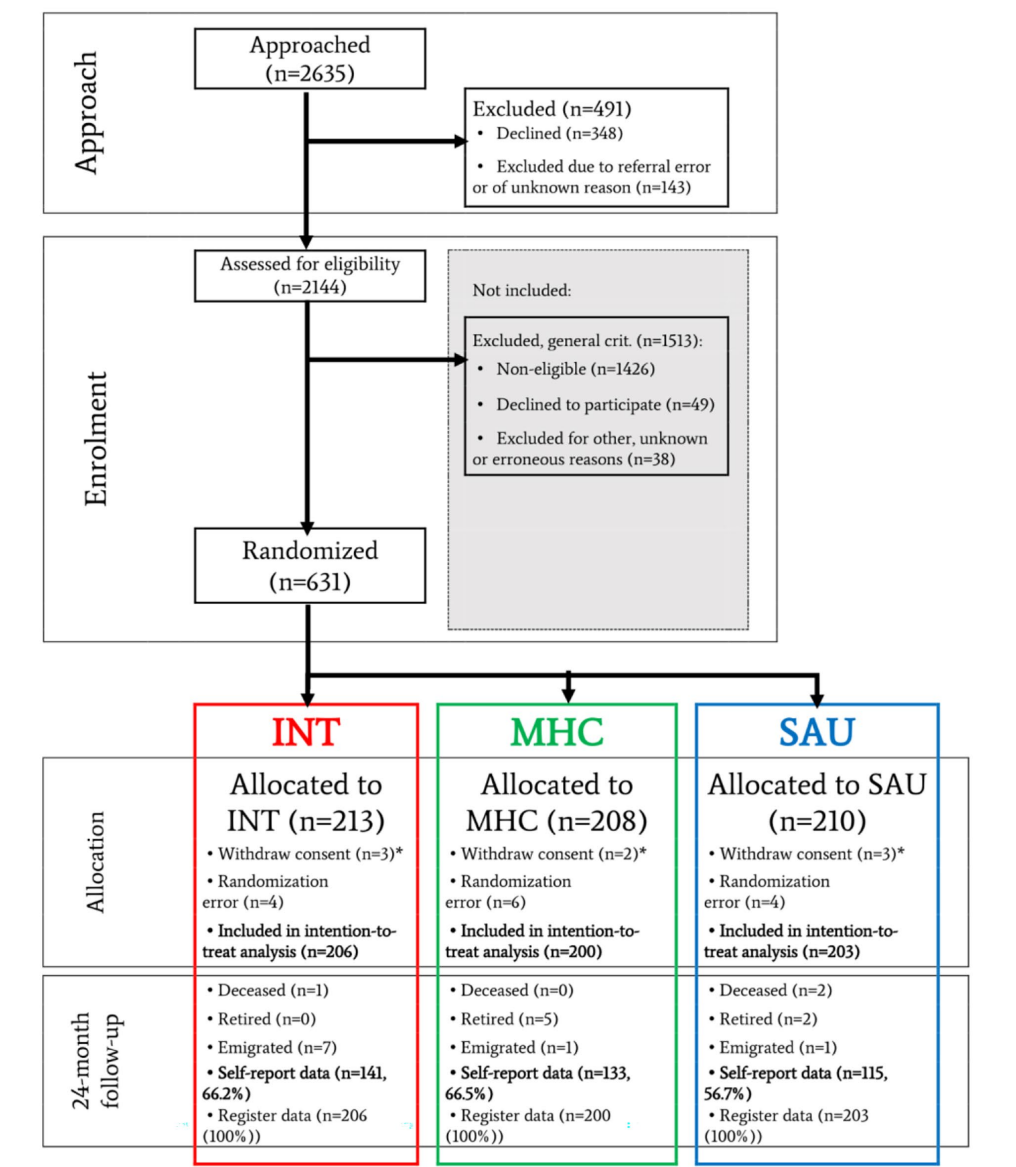
Participant flow. (INT: Integrated intervention; MHC: Mental health care; SAU: Service as usual; RCT: Randomized controlled trial; *Due to European data legislation, complete deletion of data was possible and was requested after randomization by 8 participants)

In total, 2635 sickness absentees were approached to participate in the mental health assessment, and 2144 were assessed. Of these, 631 met the inclusion and exclusion criteria for this study and were randomized. Due to the right to withdraw all information, we have no data (besides intervention allocation) on 8 of the randomized participants, and 14 participants were excluded due to randomization errors(they were randomized despite ineligibility). Altogether, 609 participants were included in intention-to-treat analyses. Of these, 19 participants were censored in survival and proportion-in-work analyses because they had died, emigrated or retired at follow-up. Figure 1 illustrates participant flow and drop-out.

### Baseline Characteristics

The mean age was 41.9 years (SD 10.8), and considerably more women (73.6%) than men (26.4%) were included. Notably, 22.5% were on sick leave from unemployment. A majority of trial participants were diagnosed with depression (63.5%). As the only skewed variable at baseline (p = 0.041), age was included as co-variate in the sensitivity analysis. Table 1 shows the baseline sociodemographic, vocational and clinical characteristics of study participants.

**Table 1:** Baseline characteristics of trial participants according to trial-arm. Abbreviations: BAI: Beck Anxiety Inventory; BDI: Beck Depression Inventory; MHC: Mental Healthcare; PSS: Perceived Stress Scale; SD: Standard Deviation; VR: Vocational rehabilitation; WSAS: Work and Social Adjustment Scale; a: missing data = 0.7%; b: missing data = 2.6%; c: missing data = 0.2%

		Participants, no. (%)		
		SAU (n = 203)	MHC (n = 200)	INT (n = 206)
**Age in years**, **mean (SD)**		43.08 (11.03)	42.17 (11.01)	40.42 (10.33)
**Sex**	Female	151 (74.4)	147 (73.5)	150 (72.8)
	Male	52 (25.6)	53 (26.5)	56 (27.2)
**Highest educational level achieved**	Primary school	28 (13.8)	26 (13)	27 (13.1)
	Secondary or vocational education	78 (38.4)	79 (39.5)	72 (35)
	Bachelor’s degree or above	97 (47.8)	95 (47.5)	107 (51.9)
**Municipality**	Copenhagen	129 (63.5)	123 (61.5)	124 (60.2)
	Gentofte	16 (7.9)	17 (8.5)	18 (8.7)
	Gladsaxe	33 (16.3)	35 (17.5)	38 (18.4)
	Lyngby-Taarbæk	25 (12.3)	25 (12.5)	26 (12.6)
**Vocational status**	Employed	158 (77.8)	154 (77.0)	160 (77.7)
	Unemployed	45 (22.2)	46 (23.0)	46 (22.3)
**Level of depression per BDI, mean (SD)** ^**a**^		27.05 (9.54)	27.71 (9.63)	28.12 (9.08)
**Level of anxiety, BAI, mean (SD)** ^**a**^		21.58 (9.71)	21.68 (9.35)	22.33 (8.51)
**Level of stress, PSS, mean (SD)** ^**a**^		25.97 (5.58)	25.45 (5.07)	26.04 (5.10)
**Level of functioning, WSAS, mean (SD)** ^**b**^		25.44 (7.37)	26.08 (7.42)	25.84 (7.07)
**Sick leave weeks before baseline, mean (SD)** ^**c**^		10.84 (4.44)	11.32 (3.74)	11.22 (4.29)
**Diagnosis**	Anxiety	72 (35.5)	73 (36.5)	77 (37.4)
	Depression	131 (64.5)	127 (63.5)	129 (62.6)

### Vocational Outcomes

**Fig. 2:**
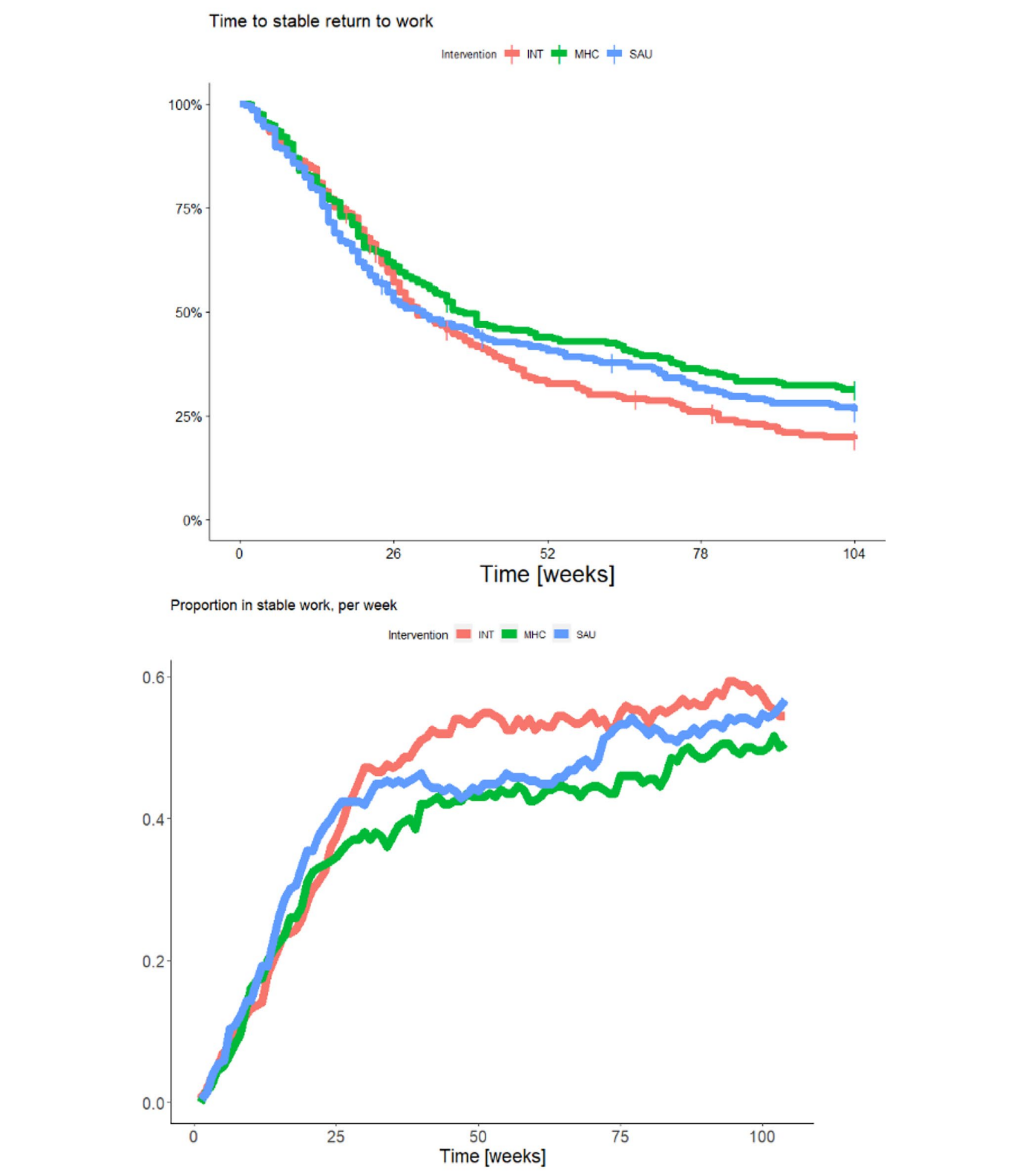
Kaplan-Meier curve for the event ‘first stable return to work’ (top) and proportion in work (bottom); INT: IBBIS Integrated Intervention; MHC: IBBIS Mental Healthcare; SAU: Service As Usual

At 24-month follow-up, the average numbers of weeks in work (of 104 possible weeks) were as follows: SAU 44.5 (SD 9.5); MHC 40.4 (SD 9.0); INT 48.5 (SD 10.5). RTW rates in the SAU group did not differ from those in the MHC and INT groups. However, the MHC group showed slightly slower RTW (borderline statistical significance) compared to INT (Hazard ratio (HR) 0.79 [98.3%CI: 0.60;1.05], p = 0.044), and a borderline lower number of weeks in work compared to the INT group (Rate ratio (RR) 0.84 [98.3%CI: 0.69 to 1.01], p = 0.024). In terms of weeks in work, SAU was equal to MHC and INT. Table 2 shows the estimates of vocational outcomes. Figure 2 displays the Kaplan-Meier curve for the three groups and the curve of proportion in stable work over time. The production of the latter was a post-hoc decision. The proportion of participants working at 24-month follow-up was 54.4%, but we saw no statistically significant differences between the groups on this outcome.

**Table 2:** Vocational outcomes at 24-month follow-up: Group values and pairwise comparisons; HR: Hazard ratio; OR: Odds ratio; RR: Rate ratio; MHC: Mental Healthcare; SAU: Service As Usual; INT: Integrated Intervention; IQR: Inter-quartile Range; CI: Confidence Interval; a: n = 609. b: n = 590; *: p < 0,05

		Group values							Group comparisons										
									SAU - MHC			SAU - INT				MHC - INT			
		**INT**		**MHC**		**SAU**			HR	p (98.3% CI)		HR		p (98.3% CI)		HR		p (98.3% CI)	
Time to return to work^a^		30 [IQR: 17—81]		37 [IQR: 17—>104]		31 [IQR: 15—>104]			1,17	0,196 (0,88—1,55)		0,93		0,523 (0,71—1,22)		0,79		*0,0444 (0,6—1,05)	
		**INT**		**MHC**		**SAU**			OR	p (98.3% CI)		OR		p (98.3% CI)		OR		p (98.3% CI)	
Proportion in work^a^		56.9%		51%		57.5%			1,32	0,176 (0,81—2,17)		1,05		0,826 (0,64—1,72)		0,79		0,24 (0,48—1,29)	
		**INT**		**MHC**		**SAU**			RR	p (98.3%CI)		RR		p (98.3%CI)		RR		p (98.3%CI)	
Weeks of work^b^		48.5 [SD: 10.5]		40.4 [SD: 9]		44.5 [SD: 9.5]			1,1	0,229 (0,91—1,35)		0,93		0,31 (0,78— 1,11)		0,84		*0,024 (0,69—1,01)	

#### Reccurring Sick Leave

Reccurring long-term sick leave (≥ 4 weeks) affected 99 (22.2%) of the 445 participants who returned to work. Of the 149 persons who returned to work in the SAU group, 26 (17.5%) experienced reccurring sick leave. In the MCH group, the corresponding number was 32 (23.5%) of 136 participants, and in the INT group 41 (25.6%) of 160 persons.

### Symptom, Functioning, Self-efficacy, Quality of Life, and Presenteeism

At 24-month follow-up, we observed no statistically significant differences between any of the self-reported outcomes: symptoms of anxiety, depression or stress, level of functioning, self-efficacy, quality of life, and presenteeism. These results are presented in Supplement 5.

### Subgroup and Sensitivity Analyses

Subgroup analyses for all outcomes (including test for interaction between stratification variables and intervention on all vocational outcomes) are shown in Supplement 6. Only selected and significant subgroup or interaction analyses for outcomes at 24 months are described here. When comparing INT with the other two groups, interaction was seen for the variable baseline diagnosis (anxiety vs. depression) on RTW rates and proportion in work. This was supported by subgroup analyses of persons with anxiety in which the MHC group showed considerably poorer vocational outcomes than the other groups. This tendency was not seen among participants with baseline depression; here, no significant differences between the three groups were observed.

In the small subgroup of participants without employment at baseline (132 of 609, 21%), the INT group had worked 39.4 weeks (SD 7.6) at 24-month follow-up. In the other groups, the number was 28.6 weeks (SD 7.0) for SAU and 27.3 weeks (SD 6.6) for MHC. Yet, these differences were not statistically significant (p-values 0.17 and 0.08, respectively).

Sensitivity analyses of all questionnaire outcomes are shown in Supplement 7. Best/worst-case scenarios showed substantial deviations from the main results. This was expected because of the low response rates and large differences in response rates between trial arms (between 56.7% and 66.5%).

## Discussion

This trial was conducted to test if INT would improve the RTW process of persons on sick leave with anxiety or depression. The long-term results of this trial do not generally support our hypotheses, as SAU and INT differed neither on vocational nor on self-assessed outcomes. Compared to MHC, though, INT showed some vocational benefits in terms of weeks in work and time to RTW.

Establishing true integration of the intervention components in INT proved to be a difficult, time-consuming venture that was complicated by distrust and diverging goals and norms between the involved organizations [21, 27]. Thus, the integrational features were only implemented to a fair degree [14]. The 6-month follow-up showed that INT did not improve RTW rates (primary outcome) compared to SAU. However, despite these implementation difficulties, the proportion in work was somewhat higher at 12-month follow-up [14], and at 6-month follow-up (but not at 12- and 24- month follow-up) INT yielded slightly lower levels of stress (borderline statistical significance) and significantly higher satisfaction with services compared to SAU.

The disappearance of effect on proportion in work, specifically between 12- and 24-month follow-up, could be due to several factors. First, the interventions in INT spanned several months, and for many participants it lasted more than 6 months after randomization. Despite the fact that a substantial proportion of the participants had still not returned to work 12 months after randomization, practically nobody received any interventions beyond that point. This could explain why no effect was seen thereafter. If so, a long-term effect might be achievable if the intervention is continued for participants who have still not returned to work after 12 months. Second, the disappearance of effect on this outcome may be caused by spontaneous remission to a ceiling level in the SAU group between 12- and 24-month follow-up, while this ceiling was reached just before 12-month follow-up in the INT group as a result of the intervention. If that explains most of the difference in effect, this group (at baseline) should still be considered at high risk of long-term negative vocational outcomes that call for the development of effective interventions. Third, it may matter that some of the practitioners delivering the interventions were only trained for a rather short period of time and hence lacked experience, unlike in other studies of effective RTW interventions (see for example [13]). Yet, the practitioners were trained in regular CBT, and despite the short training period, this intervention has proved effective in other studies of similar healthcare interventions delivered to a similar population (personal communication, publication in preparation). Thus, the quality of the delivered CBT should in itself ensure symptom reduction. Fourth, the disappearance of effect may be due to the low quantity of sessions in INT compared to SAU. Because previous studies have found healthcare in primary care to be insufficient [28, 29], we had expected an effect just from providing relevant care, without integration, as in the MHC group. Yet, in the SAU group, participants reported receiving on average 9.5 psychotherapy sessions (SD 6.2), which cannot be regarded as insufficient. Therefore, we cannot conclude that the interventions in MHC and INT clearly improved healthcare compared to SAU.

### Other Evidence

One other trial has tested an integrated mental healthcare and vocational intervention: the Norwegian At Work and Coping (AWaC) intervention for persons either at risk of or on actual sick leave or on long term health-related benefits. Here, 7.8% more persons had increased work hours or stable work after the integrated intervention compared to SAU after 18 months (p = 0.018) [11]. Our study showed no difference in proportion in work at 24-month follow-up (p = 0.83). The work-directed component and the integration of the AWaC intervention were also difficult to implement. The AWaC intervention did not produce a statistically significant effect on the subgroup of persons on full-time sick leave [30]. The slightly better effects from the AWaC intervention might be explained by the work-directed character of the therapy [11], which differed from the conventional CBT in our intervention. This interpretation is supported by research indicating that CBT should be work-focused if the aim is to improve vocational outcomes. Yet, this effect was most pronounced in the subgroup of people on sick leave with a high score of return-to-work self-efficacy [13].

### Strengths and Limitations

This trial has a sufficiently large sample size and thus relevant power on primary and secondary outcomes. However, the study has some serious limitations that prevent strong conclusions. Like in similar RTW trials, workplace-directed service components were only implemented to a fair degree [31]. Workplace-directed interventions are well-established, effective intervention components for faster RTW [7, 32]. These integration problems lower the internal validity of the findings and hinder strong conclusion about effect. Furthermore, blinding was not possible with service users, service providers and case managers who determined eligibility for benefit and thus affected DREAM register data. Finally, the novel IBBIS Vocational Rehabilitation intervention was not tested separately in this trial, and any effect or absence of effect cannot be ascribed to that specific intervention, the integration of vocational rehabilitation and healthcare or any combination thereof.

### Implications and Generalizability

Multi-disciplinary and integrated interventions using elements such as roundtable meetings and co-location are difficult to implement and demand great effort from the involved organizations [10]. The implementation problems in INT have several implications. First, the problems with internal validity affect external validity and generalization; we cannot rule out that the true effect, in a situation with full implementation, would be larger or smaller than the observed effect. Therefore, we need more research on well-implemented integrated interventions if any clear recommendations for practice are to be made. Second, implementation difficulties are very common for this type of intervention [30, 31] and reduce the feasibility and usefulness of the intervention. In view of future trials and potential routine practice, integrated interventions like INT should be delivered with considerable managerial attention and caution. Furthermore, international generalizability of the results is affected by the differences in healthcare provision and social insurance systems and legislation between countries [18]. A process evaluation of the intervention components in INT showed that interprofessional collaboration, service users’ perception of the intervention and service users’ RTW process were influenced by the national sickness benefit legislation and the jobcenters’ reputation and demands [21, 27]. Thus, both the delivery and effects of this type of intervention are highly context-dependant. Finally, the aim of improving vocational outcomes may require that practitioners are specially trained to deliver work-focused interventions and not just regular mental healthcare. This could affect the feasibility of large-scale implementation, since a large group of practitioners will need additional qualifications.

## Conclusion

This trial compared INT to SAU and MHC. Against our hypothesis, long-term results showed that, INT did not cause improved vocational status, symptom levels, quality of life or self-efficacy compared to SAU at 24-month follow-up. However, compared to SAU, INT showed slightly greater stress symptom reduction at 6-month follow-up and a slightly higher proportion in work at 12-month follow-up. Compared to MHC, INT showed better vocational outcomes at both 12- and 24-month follow up but no effect in terms of self-reported health. INT was only implemented to a fair degree throughout the trial, and further research should be conducted to affirm the effect and feasibility of integrating healthcare with vocational rehabilitation in similar welfare settings.

## Electronic Supplementary Material

Below is the link to the electronic supplementary material.

Supplementary Material 1

## Data Availability

The datasets generated during the current study are available from the corresponding author on reasonable request, if relevant legislation and required data protection measures are met.

## Abbreviations

BAI: Beck Anxiety Inventory
BDI: Beck Depression Inventory
CONSORT: Consolidated Standards of Reporting Trials
IBBIS: Integreret Behandlings- og Beskæftigelsesindsats til Sygedagpengemodtagere med depression, angst eller stress [In Danish] (Translated:Integrated mental healthcare and vocational rehabilitation to improve return-to-work rates for people on sick leave because of common mental disorders)
IPS: Individual Placement and Support MHC:Mental Healthcare
PSS: Perceived Stress Scale
SD: Standard Deviation
VR: Vocational rehabilitation
WSAS: Work and Social Adjustment Scale
RTW: Return to work
