# Supplementary material for: Integrated Mental Healthcare and Vocational Rehabilitation for People on Sick Leave with Anxiety or Depression: 24-Month Follow-up of the Randomized IBBIS Trial
Source: J Occup Rehabil. 2023 Feb 27;33(3):570–80. doi: 10.1007/s10926-023-10094-7 (PMC10495506; doi:10.1007/s10926-023-10094-7)
Supplement: Supplementary file 1 — Supplementary Material 1 [file 10926_2023_10094_MOESM1_ESM.pdf]

Supplement 1: changes to trial design reflected in statistical analysis plan

- In April, 2017, mid inclusion, a small and technical alteration to the inclusion criteria was made from " Eligible participants in this trial are adults who are on sick leave from work or unemployment and have received sick leave benefit for a minimum of four weeks." to "Eligible participants in this trial are adults who are on sick leave from work or unemployment and have received sick leave benefit for minimum four weeks or have started a sick leave benefit case which is estimated to last for minimum eight weeks". This alteration was done to compensate for a concurrent legislative change were vocational support could be provided before the first day of sickness benefit refusion.
- In the statistical analysis plan we planned statistical assumption control of proportional hazards in cox-regression, and that we would alternatively adjust for different kinds of interactions between time and group assignment. In the primary outcome analysis of difference between the INT and SAU groups we found non-proportional hazards, but did not adjust for time, despite that, since the original intention was to conservatively test a rejection of the null-hypothesis – not vice versa.
- We discarded the outcome time from the first day of return to work until recurrent sick leave, since we realized that this time would not consistently reflect a positive outcome due to the high risk of bias.
- Neither the statistical analysis plan nor the study design article mentioned presenting a proportion per time-curve. Post-hoc we decided that this would be beneficial.
- In the statistical analysis plan, we had planned adjusted analyses with any variables that differed at baseline. Though age was unevenly distributed at baseline, we did not adjust for age in main or sensitivity analyses.

Supplement 2: Eligibility assessment

Assessment of eligibility was performed by research staff, all clinically trained mental health professionals, supervised by a psychiatric specialist, and specifically trained for the purpose. The assessment was conducted trough clinical interview, partly guided by 1) MINI International Neuropsychiatric Interview,(1) 2) Standardized Assessment of Personality - Abbreviated Scale (SAPAS) (2), 3) Attention deficit hyperactivity disorder symptom checklist for adults (Adult Self-Report Scale, ASRS v1·1)(3), and 4) Mini-Mental State Examination (MMSE)(4) (when dementia was clinically suspected).

Eligible were ≥18 year old sick leave benefit recipients for ≥ four weeks with an ICD10 diagnosis of either depression (F32-F33), generalized anxiety disorder (F41.1), social phobia (F40.1) or panic disorder (F41.0). Participants were required to have Danish proficiency and give written consent. Excluded were those who were pregnant, in at least moderate risk of suicide according to clinical assessment and stratification according to the MINI International Neuropsychiatric Interview (1), had clinically significant substance abuse disorder according to clinical assessment by assessor, other unstable medical condition judged by assessor to yield significant obstacles for mental health care treatment in the research project, showed signs of dementia by assessors' clinical judgement, or could not indicate willingness to abstain from seeking mental health care outside the study, whilst receiving such through the study.

References

1. Sheehan D, Lecrubier Y, Sheehan KH, Amorim P, Janavs J, Weiller E, et al. The Mini-International Neuropsychiatric Interview (M.I.N.I.): The development and validation of a structured diagnostic psychiatric interview for DSM-IV and ICD-10. In: Journal of Clinical Psychiatry. 1998. p. 22–33.

2. Moran P, Leese M, Lee T, Walters P, Thornicroft G, Mann A. Standardised Assessment of Personality – Abbreviated Scale (SAPAS): Preliminary validation of a brief screen for personality disorder. Br J Psychiatry. 2003 Sep 2;183(3):228–32.

3. Kessler RC, Adler L, Ames M, Demler O, Faraone S, Hiripi E, et al. The World Health Organization Adult ADHD Self-Report Scale (ASRS): a short screening scale for use in the general population. Psychol Med. 2005;35:245–56.

4. Cole MG, McCusker J, Elie M, Dendukuri N, Latimer E, Belzile E. Systematic detection and multidisciplinary care of depression in older medical inpatients: a randomized trial. Can Med Assoc J. 2006 Jan;174(1):38–44.

Supplement 3: Outcomes

**Outcome class**	**Data source**	**Outcome**
Secondary outcome	DREAM database	Time from baseline to RTW
Pre-defined explorator y outcomes	DREAM database	Weeks in stable work (min 4 weeks) from baseline to current follow-up
		Weeks in stable work (min 8 weeks) from baseline to current follow-up
		Weeks in stable work (min 12 weeks) from baseline to current follow-up
		Weeks in work from baseline to current follow-up
		Proportion in ordinary work
		Number of recurrent sick leaves
	Questionnaires	Symptoms of Distress, anxiety, depression and somatization by Four-Dimensional Symptom Questionnaire (4DSQ)(1)
		Depressive symptoms measured by Beck Depression Inventory (BDI)(2)
		Anxiety symptoms measured by Beck Anxiety Inventory (BAI)(2)
		Stress-symptoms measured by Cohen perceived stress scale (PSS)(3)
		Social and work related function measured by WSAS(4)
		Burn-out symptoms measured by Karolinska Exhaustion Scale (KES)(5)
		Health-related quality of life measured by EQ-5D-5L(6)
		General Quality of life scale measured by Flanagan's' QOLS(7)
		Self-efficacy concerning symptoms measured by IPQ subscale on personal control(8)
		Return to work self-efficacy measured by RTW-SE(9)
		General self-efficacy measured by General Self-efficacy scale (GSS)(10)
		Presenteeism measured by Stanford Presenteeism Scale (SPS)(11)

Table moderated from Statistical analysis plan (12)

Definition of the beneficial outcome direction for all outcomes (6, 12, and 24-month-follow-up)

**Outcome**	Is "better outcome" defined by lower or higher numbers?	
Time from baseline to RTW	Lower	
Proportion in stable work		Higher
Time from baseline to RTW	Lower	
Weeks in work (all variations of stability definitions)		Higher
Number of recurrent sick leaves	Lower[1]	
Depressive symptoms measured by Beck Depression Inventory (BDI)(13)	Lower	
Anxiety symptoms measured by Beck Anxiety Inventory (BAI) (2)	Lower	
Stress symptoms measured by Cohen perceived stress scale (PSS)(3)	Lower	
Social and work related function measured by WSAS(4)	Lower	
Symptoms of Distress, anxiety, depression and somatization by Four-Dimensional Symptom Questionnaire (4DSQ)(1)	Lower	
Burn-out symptoms measured by Karolinska Exhaustion Scale (KES)(5)	Lower	
Health-related quality of life measured by EQ-5D-5L(6)		Higher(14)
General Quality of life scale measured by Flanagan's' QOLS(7)		Higher
Self-efficacy concerning symptoms measured by IPQ subscale on personal control(8)		Higher(15)
Return to work self-efficacy measured by RTW-SE(9)		Higher
General self-efficacy measured by General Self-efficacy scale (GSS)(10)		Higher
Client satisfaction with treatment measure measured by CSQ-8(16)		Higher
Presenteeism measured by Stanford Presenteeism Scale (SPS)(11)		Higher(17)

Table moderated from Statistical analysis plan (12)

[1] A low number of recurrent sick leave is a positive outcome only if duration of index sick leave is ideally balanced between compared groups.

References

1. Terluin B, van Marwijk HWJ, Adèr HJ, de Vet HCW, Penninx BWJH, Hermens MLM, et al. The Four-Dimensional Symptom Questionnaire (4DSQ): a validation study of a multidimensional self-report questionnaire to assess distress, depression, anxiety and somatization. BMC Psychiatry. 2006;6:34.

2. Beck AT, Steer RA, Carbin MG. Psychometric properties of the Beck Depression Inventory: Twenty-five years of evaluation. Clin Psychol Rev. 1988;8(1):77–100.

3. Cohen S, Kamarck T, Mermelstein R. A global measure of perceived stress. J Health Soc Behav. 1983;24(4):385–96.

4. Mundt JC, Marks IM, Shear MK, Greist JH. The Work and Social Adjustment Scale: A simple measure of impairment in functioning. Br J Psychiatry. 2002;180(MAY):461–4.

5. Saboonchi F, Perski A, Grossi G. Validation of Karolinska Exhaustion Scale: Psychometric properties of a measure of exhaustion syndrome. Scand J Caring Sci. 2013;27(4):1010–7.

6. Brazier J, Roberts J, Tsuchiya A, Busschbach J. A comparison of the EQ-5D and SF-6D across seven patient groups. Health Econ. 2004;13(9):873–84.

7. Burckhardt CS, Anderson KL. The Quality of Life Scale (QOLS): reliability, validity, and utilization. Health Qual Life Outcomes. 2003;1(1):60.

8. Moss-Morris R, Weinman J, Petrie KJ, Horne R, Cameron LD, Buick D. The revised Illness Perception Questionnaire (IPQ-R). Psychol Health. 2002;17(1):1–16.

9. Lagerveld SE, Blonk RWB, Brenninkmeijer V, Schaufeli WB. Return to work among employees with mental health problems: Development and validation of a self-efficacy questionnaire. Work Stress. 2010 Oct;24(4):359–75.

10. Schwarzer R, Jerusalem M. Generalized Self-Efficacy scale. In: Wright S, Johnston M, editors. Measures in health psychology: A user's portfolio Causal and control beliefs. Windsor, England; 1995. p. 35–7.

11. Koopman C, Pelletier KR, Murray JF, Sharda CE, Berger ML, Turpin RS, et al. Stanford presenteeism scale: health status and employee productivity. J Occup Environ Med. 2002;44(1):14–20.

12. Hoff A, Fisker J, Poulsen RM, Hjorthøj C, Eplov LF. Statistical Analysis Plan [SAP] - version 2 IBBIS. 2020.

13. Beck AT, Brown G, Epstein N, Steer RA. An Inventory for Measuring Clinical Anxiety: Psychometric Properties. J Consult Clin Psychol. 1988;56(6):893–7.

14. EuroQol Group. EuroQol–a new facility for the measurement of healthrelated quality of life. The EuroQol Group. Health Policy (New York) [Internet]. 1990;16(3):199–208. Available from: http://www.ncbi.nlm.nih.gov/pubmed/10109801

15. Weinman J, Petrie KJ, Moss-Morris R, Horne R. The illness perception questionnaire: A new method for assessing the cognitive representation of illness. Psychol Heal. 1996;11(3):431–45.

16. Attkisson C, Zwick R. The Client Satisfaction Questionnaire: Psychometric properties and correlations with service utilization and psychotherapy outcome. Eval Program Plann. 1982;5:233–7.

17. Koopman C, Pelletier KR, Murray JF, Sharda CE, Berger ML, Turpin RS, et al. Stanford Presenteeism Scale: Health status and employee productivity. J Occup Environ Med. 2002;44(1):14–20.

Supplement 4: IBBIS Statistical Analysis Plan

The following pages contains the pre-registered IBBIS Statistical Analysis Plan

Statistical Analysis Plan [SAP]- version 2

Version 1: July, 2019

Version 2: November 30th, 2020

IBBIS

Integrated mental health care and vocational rehabilitation to improve return to work rates for people on sick leave because of common mental disorders (IBBIS)

IBBIS Statistical Analysis Plan [SAP] - version 2.

Authors: Andreas Hoff[1] (corr. author), Jonas Fisker[1], Rie Mandrup Poulsen[1], Carsten Hjorthøj[1,2], and Lene Falgaard Eplov[1]

[1] Copenhagen Research Center for Mental Health [CORE], Mental Health Services Capital Region of Denmark, University of Copenhagen, Gentofte Hospitalsvej 1, Opgang 15-4, DK-2900
[2] University of Copenhagen, Department of Public Health, Section of Epidemiology

Correspondence: andreas.hoff@regionh.dk

Abbreviations:

DREAM: Den Registerbaserede Evaluering Af Marginaliseringsomfanget (Danish)
DPCR: Danish Psychiatric Central Register
IBBIS: Integreret Beskæftigelses- og Behandlingsindsats til Sygedagpengemodtagere (Danish)
LPR: National Patient Register [In Danish: Landspatientregisteret]
RCT: Randomized controlled trial
RTW: Return to work
SDA: Study Design Article

ADMINISTRATIVE INFORMATION

The structure of this SAP is largely aligned with the recommendations by Gamble et. al[1].

1.1 Title and trial registration

This SAP is the detailed statistical analysis plan, expanding the scientific IBBIS protocol of the two IBBIS randomized clinical trials (ClinicalTrials.gov Identifiers: NCT02872051 (RCT1[1]) and NCT02885519 (RCT2[2])):

RCT1: "Integrated Mental Health Care and Vocational Rehabilitation to Individuals on Sick Leave Due to Anxiety and Depression (IBBIS)"

and

RCT2: "Integrated Mental Health Care and Vocational Rehabilitation to Individuals on Sick Leave Due to Stress Disorders (IBBIS)".

Due to extensive methodological similarities between these studies this SAP applies to both, unless differences are mentioned explicitly.

1.2 SAP version

This is the second version of the SAP.

[1] https://clinicaltrials.gov/ct2/show/NCT02872051

[2] https://clinicaltrials.gov/ct2/show/NCT02885519

Differences between version 1 and 2 are explicitly stated through a .docx-version of this newest version, where all changes are tracked, using the *Track changes* function in Microsoft Office Word. This file will be readily mailed through the corresponding author.

In brief, main changes revolves around the 24-month explorative outcomes: after analyses of 6- and 12-month outcomes we realized that results across work outcomes were heterogenous to a higher extent than expected. E.g., while the SAU group tended to have fastest RTW at 6-month follow-up, from explorative proportion over time-curves we realized that they might also tend to have a higher degree of recurrent sick-leave. Despite this, the SAU and INT groups still showed approx. the same number of weeks in work (when stability is disregarded) at 12-month follow-up. Followingly we speculate that the SAU group experiences faster RTW, but more recurrent sick leave. Therefore, we suggest that number of weeks in stable work is a better outcome, since this number is only high if RTW happens early, and if it is stable, and not disrupted by sick leave recurrence. We do though not know what stability threshold we should apply, and to *explore* this, we defined, prior to 24-month analyses, three different outcomes, with three different thresholds, see section 6.4. We plan these as sensitivity analyses.

1.3 Protocol version

Previous to publication of this SAP, plans have been described in both the protocol (published on clinicaltrials.org in the links provided), as well as in two study design articles (SDA), corresponding to the two RCTs[2,3].

2 INTRODUCTION: BACKGROUND, RATIONALE AND OBJECTIVES

Described thoroughly in the SDAs[2,3]. Furthermore, the protocol was published[3] on the official webpage of the organization (Mental Health Services, Capital Region of Denmark).

3 STUDY METHODS

3.1 Trial design

See SDAs[2,3].

3.2 Randomization

From SDA[2]:

“The allocation ratio between the three arms is 1:1:1. A centralized randomization will take place according to a web-based computer-generated allocation sequence with varying block sizes kept unknown to the assessors. Odense Patient data Explorative Network (OPEN) is responsible for the randomization, administrative personnel in the IBBIS team perform the online randomization and the IBBIS team leader assign the participant to interventions and professionals.

We expect that service delivery can vary from municipality to municipality and the process of gaining a new job from unemployment will take longer time than returning to an existing job. Previous research has shown that diagnosis is a possible predictor of return to work[4]. Thus, the randomization is stratified according to 1) municipality 2) employment status (on sick leave from work vs. on sick leave from unemployment) 3) diagnosis […]”

In RCT 1 diagnosis stratification is depression versus anxiety as primary diagnosis, and in RCT 2 diagnosis stratification is burnout vs. distress vs. adjustment disorder as primary diagnosis.

3.3 Sample size

Replicated from protocol follows:

[3] https://www.psykiatri-regionh.dk/Kvalitet-og-udvikling/udvikling/ibbis/Sider/IBBIS-forskning.aspx

The sample size is based on a sample size calculation, using the 'Power and Sample Size' calculation programme[4]*.*

Type I error (α) risk

In each of the two RCTs we wish to conduct multiple comparisons (between 3 groups), and hence significance level must be as follows, due to Bonferroni correction:

$$\alpha = \frac{0{,}05}{3} = \frac{1}{60} = 0{,}0167$$

Type II error (β) risk

The organizational constellation of the interventions has not yet been trialled, and thus the desired power shall be set to:

$$\beta = 0{,}9$$

If it turns out that we cannot include enough participants, the power could be set to: $\beta = 0{,}8$

Hazard ratio (R)

The mean difference in time for return to work will be calculated as a hazard ratio. We estimate that as sufficient HR is

$$R = 1{,}5$$

since just 50 % faster return to work time in the intervention groups will convey a relevant economic benefit, due to the hence smaller loss of productivity.

Mean time to return to work (M_1)

Number of days from baseline to return to work is conservatively estimated to be 210 days, after an observed range from 104 to 210 days, in the control groups in three Dutch RCTs[5–7]*, which were comparable to the control groups in the IBBIS RCTs.*
Hence,

$$M_1 = 210$$

Inclusion time period (A)

We will include participants through 24 months,

$$A = 730[days]$$

Ratio between groups (m)

Ratio is 1:1:1, and hence m = 1

Follow-up time (F)

We will follow participants up for 365 days, in which they will contribute with risk time in the survival analysis, hence F = 365

Result

In each group, due to the above-mentioned variables, we need

***198** participants per group, and with three groups that yields a need for, with power = 0.9,*

[4] http://ps-power-and-sample-size-calculation.software.informer.com

$$N = 198\,\frac{\text{participants}}{\text{group}} \times 3\,\frac{\text{groups}}{\text{trial}} = 594\text{ participants}$$

If, in case of insufficient inclusion possibilities, power could be lowered to 0.8. In such case we would need the following number:

$$N = 153\,\frac{\text{participants}}{\text{group}} \times 3\,\frac{\text{groups}}{\text{trial}} = 459\text{ participants}$$

3.4 Statistical interim analyses and stopping guidance

No interim analysis will be performed. We planned no stopping guidance.

3.5 Timing of final analysis

The researchers who will perform the 6- and 12-month outcome analyses (AH and JF) will be blinded from intervention group allocation, until the primary outcome and all 12-month follow-up outcome main analyses are completed. The true randomization group allocation is concealed, with values X, Y and Z reflecting group allocation in the blinded dataset. The randomization allocation variable conversion formula is until unblinding only know and hidden by an administrative co-worker, who will not perform or assist any analysis.

At the time of publication of SAP version 1, baseline distributional analyses, and unadjusted estimated marginal means-analyses of self-reported numerical secondary outcomes at 6-month follow-up (and only these) have been calculated *blinded*, but will not be published, since this was not complying with the SDAs, nor any SAP version.

All 24-month follow-up analyses will be conducted unblinded.

4 Statistical principles

4.1 Confidence intervals and p-values

For all outcomes, the three randomization groups are pairwise compared. Due to these multiple comparisons, we will calculate 98,3% confidence intervals, according to Bonferroni correction of desired α-level of 0,05 in testing of 3 hypotheses:

α-level: $0{,}05 \times \frac{1}{3} \cong 0{,}0167 \quad \Rightarrow$ Confidence Interval: $1 - 0{,}0167 \cong 98{,}33\%$

4.2 Analysis populations

All analyses are performed as *intention-to-treat*, unless otherwise stated.

5 Trial population

5.1 Withdrawal and follow-up

Due to legislative circumstances participants can withdraw consent, and followingly all person sensitive data on these subjects will be deleted, yet participant ID number (not CPR number, but generated for this research project) and randomization result will be stored. In sensitivity analyses these ID numbers will be included, as described in "handling of missing data".

5.2 Baseline patient characteristics

The following will be reported per RCT, per randomization allocation group. For all mean values of numeric variables, standard deviations will be reported.

Total number included in RCT and number in each randomization group
Age (mean, year)
Gender (%)

Bech Depr. Inventory (mean)
Bech Anxiety Inventory (mean)
Work and Social adjustment Scale (mean)
Perceived Stress Scale (mean)
Employment status (%, employed vs. unemployed)
Primary diagnosis (%)
Municipality (%)
Sick leave duration at randomization (mean, days)
Educational level (%, short, moderate, long)

Distributional balances of these covariates (except educational level, since this is only added in SAP v. 2, after primary baseline analyses) will be calculated using one way-ANOVA for numerical data and X^2 for categorical data, and analyses with p≤0,05 will define *imbalanced baseline covariates*.

6 ANALYSIS

The first subsections of this section 6, describes general strategies applying to all analyses unless otherwise specifically stated. Subsection 6.8 contains the separate analysis strategies per outcome in 6.8.x.

6.1 Covariate adjustment in general

Analyses will be adjusted for the three stratification variables, and no other, complying with RCT analysis guidelines from European Medicines Agency[5].

6.2 Sensitivity analyses in general

As sensitivity analyses, all outcome analyses will be performed adjusted for any unbalanced baseline covariates, as defined in 5.2, *Baseline patient characteristics*.

Results of sensitivity analyses are only interpreted as supplements to the main analysis and will not substitute main results.

6.2.1 Sensitivity analyses for questionnaire based, self-reported data outcome

As sensitivity analyses, self-reported data outcomes (questionnaire-based) will be calculated with all missing outcome data replaced with a value equalling the mean of the outcome variable ± 2 standard deviations, and participants who withdraw themselves from the study will be included in these analyses with all their data handled as missing.

6.2.2 Sensitivity analyses for register data based outcomes

For register data-based outcomes, sensitivity analyses will be performed including the participants who withdraw themselves from the study, included in these analyses with all their outcomes handled as either the worst possible (never returning to work) vs best possible (returning to work as soon as possible).

Furthermore, all outcomes of number of weeks in stable return to work (outcome number 9, 10, 11 and 12), are sensitivy analyses, exploring the robustness of number of weeks in work (stability disregarded), which is oucome number 13, pre-planned before study commencement.

6.3 Subgroup analyses in general

All outcomes will be analysed with respect to the following subgroups:

a) per primary diagnosis (in RCT1 anxiety vs. depression; in RCT2 per distress, adjustment disorder, and burnout);
b) per employment status group at baseline (vacant vs. employed);

[5] https://www.ema.europa.eu/en/documents/scientific-guideline/guideline-adjustment-baseline-covariates-clinical-trials_en.pdf

c) per IBBIS Team (two teams, Team North and Team Byen)

Furthermore,

d) divided in two groups by relative time of randomization: first and last temporal half of randomized participants.

Finally,

e) we will test for interaction between diagnostic group and treatment allocation group/arm.

No outcomes have other subgroup analyses planned.

6.4 OUTCOME DEFINITIONS

The outcomes are reported as in the study design articles (except for selected outcomes, see alterations to SAP version 1 in appendix). The numbers 1 through 64 denotes the outcome numbers for reference purposes for this SAP section.

**PRIMARY AND SECONDARY OUTCOMES and outcome numbering**						
**Outcome class**	**Data source**	**Outcome**		**6-month follow-up**	**12-month follow-up**	**24-month follow-up**
Primary	DREAM database	Time from baseline to RTW			1	
Secondary	DREAM database	Proportion in ordinary work			2	
	DREAM database	Time from baseline to RTW		3		8
	Questionnaire	Depressive symptoms measured by Beck Depression Inventory (BDI)[8]		4		
	Questionnaire	Anxiety symptoms measured by Beck Anxiety Inventory (BAI)[9]		5		
	Questionnaire	Stress symptoms measured by Cohen perceived stress scale (PSS)[10]		6		
	Questionnaire	Social and work related function measured by WSAS[11]		7		

**PREDEFINED EXPLORATORY OUTCOMES and outcome numbering**					
**Outcome class**	**Data source**	**Outcome**	**Follow-up**		
			**6-month**	**12-month**	**24-month**
Pre-defined exploratory outcomes	DREAM database	Weeks in stable work (≥ 4 weeks) from baseline to current follow-up		9	10
		Weeks in stable work (≥8 weeks) from baseline to current follow-up			11
		Weeks in stable work (≥12 weeks) from baseline to current follow-up			12
		Weeks in work from baseline to follow-up			13
		Proportion in ordinary work			14
		Number of recurrent sick leaves			15
	Questionnaires	Symptoms of Distress, anxiety, depression and somatization by Four-Dimensional Symptom Questionnaire (4DSQ)[12]	16	17	18
		Depressive symptoms measured by Beck Depression Inventory (BDI)[9]		19	20
		Anxiety symptoms measured by Beck Anxiety Inventory (BAI)[9]		21	22
		Stress-symptoms measured by Cohen perceived stress scale (PSS)[10]		23	24
		Social and work related function measured by WSAS[11]		25	26
		Burn-out symptoms measured by Karolinska Exhaustion Scale (KES)[13]	27	28	29
		Health-related quality of life measured by EQ-5D-5L[14]	30	31	32
		General Quality of life scale measured by Flanagan's' QOLS[15]	33	34	35
		Self-efficacy concerning symptoms measured by IPQ subscale on personal control[16]	36	37	38
		Return to work self-efficacy measured by RTW-SE[17]	39	40	41

		General self-efficacy measured by General Self-efficacy scale (GSS)[18]	42	43	44
		Client satisfaction with treatment measure measured by CSQ-8[19]	45		
		Presenteeism measured by Stanford Presenteeism Scale (SPS)[20]	46	47	48

**HARM MEASURES and outcome numbering**					
**Outcome class**	**Data source**	**Outcome**	**Follow-up**		
			**6-month**	**12-month**	**24-month**
Harm measures	LPR	Admission to somatic hospital-based (in-patient) health care at least once		49	50
	LPR	Contact with hospital-based out-patient mental health care, at least once		51	52
		Admission to hospital-based in-patient mental health care, at least once		53	54
		Contact with emergency mental health care, at least once		55	56
	LPR	Probable self-harm, at least once		57	58
	LPR	Suicide		61	62
	LPR	Death		63	64

6.5 Hypotheses and null-hypotheses

Stated below are the generic versions of all three hypotheses (H_1) and all three null-hypotheses (H_0) that apply to each outcome.

Regarding what is a "better outcome" is listed in section 6.6, defined for each outcome measure, respectively.

6.5.1 Hypotheses

This superiority trial hypothesizes that, for all outcomes,

H_{1A} Group 3, "Integrated IBBIS mental health care treatment and vocational rehabilitation"

conveys better outcomes than

Group 2, "IBBIS mental health care (and standard VR)", and

H_{1B} Group 2, "IBBIS mental health care (and standard VR)", and

conveys better outcomes than

Group 1, "Control group, treatment as usual (standard MHC and standard VR)"

and followingly

H_{1C} Group 3, "Integrated IBBIS mental health care treatment and vocational rehabilitation"

conveys better outcomes than

Group 1, "Control group, treatment as usual (standard MHC and standard VR)".

and followingly

Group 3 conveys better outcomes than Group 1,

since if

Group 3 outcome > Group 2 outcome > Group 1 outcome

then

Group 3 outcome > Group 1 outcome.

The groups are thoroughly described in the IBBIS Protocol and the SDAs.

6.5.2 Null-hypotheses

The corresponding null-hypotheses are

H_{0A} Group 3, “Integrated IBBIS mental health care treatment and vocational rehabilitation”

does not convey better outcomes than

Group 2, “IBBIS mental health care (and standard VR)”, and

H_{0B} Group 2, “IBBIS mental health care (and standard VR)”,

does not convey better outcomes than

Group 1, “Control group, treatment as usual (standard MHC and standard VR)”.

and followingly

H_{0C} Group 3 *does not* convey better outcomes than Group 1.

6.6 OUTCOME BENEFIT DIRECTION

Referring to the hypothesis section, this table describes whether a "better outcome" is a higher or lower score on the numeric outcome variables.

**Outcome**	Is "better outcome" defined by lower or higher numbers?	
Time from baseline to RTW	Lower	
Proportion in stable work		Higher
Time from baseline to RTW	Lower	
Weeks in work (all variations of stability definitions)		Higher
Number of recurrent sick leaves	Lower[6]	
Depressive symptoms measured by Beck Depression Inventory (BDI)[8]	Lower	
Anxiety symptoms measured by Beck Anxiety Inventory (BAI)[9]	Lower	
Stress symptoms measured by Cohen perceived stress scale (PSS)[10]	Lower	
Social and work related function measured by WSAS[11]	Lower	
Symptoms of Distress, anxiety, depression and somatization by Four-Dimensional Symptom Questionnaire (4DSQ)[12]	Lower	
Burn-out symptoms measured by Karolinska Exhaustion Scale (KES)[13]	Lower	
Health-related quality of life measured by EQ-5D-5L[14]		Higher[21]
General Quality of life scale measured by Flanagan's' QOLS[15]		Higher
Self-efficacy concerning symptoms measured by IPQ subscale on personal control[16]		Higher[22]
Return to work self-efficacy measured by RTW-SE[17]		Higher
General self-efficacy measured by General Self-efficacy scale (GSS)[18]		Higher
Client satisfaction with treatment measure measured by CSQ-8[19]		Higher
Presenteeism measured by Stanford Presenteeism Scale (SPS)[20]		Higher[23]

[6] A low number of recurrent sick leave is a positive outcome only if duration of index sick leave is ideally balanced between compared groups.

6.7 Missing data in general

In general, proportion of missing data will be reported per intervention group for all outcomes.

6.7.1 Handling of missing data in registers

For RTW-outcomes (outcomes based on the DREAM register) we expected *no missing data*, due to the nature of the Dream Register, prior to study inception. Missing data should only be in case of a participant moving out of Denmark. We considered these events to be so rare in our data that we would handle such missing data as *missing completely at random*. Thus, no imputation or other correction was considered necessary. We will report proportion of data missing.

We will report number of censored participants per treatment group.

At the of this updated version 2a of the SAP, we have realized that some data were missing due to DREAM database errors, against expectation. We included the cases with missing data in sensitivity analyses to explore the potential impact of the missingness.

6.7.2 Handling of missing data in questionnaire based, self-reported data outcome

For questionnaire-based outcomes, missing data will be handled as *missing at random*. To handle this, 100 multiple imputations will be performed, using following variables: stratification variables: diagnosis, municipality, employment status; age; gender; time to stable RTW; psychometric variables at baseline and all follow-up at outcome time: BDI, BAI, WSAS and PSS.

6.8 Analysis methods per outcome group

This section describes the details of the statistical analyses. Since several outcomes require exact same analysis methods, outcomes are grouped for the following description

6.8.1 Time to return to work-outcomes (outcomes #1, #3 and #8)

This section describes primary outcome *Time from baseline to RTW* at 12-month follow-up (1), and the secondary outcomes *Time from baseline to RTW* at 6- (outcome 3) and 24-month follow-up (outcome 8). The 24-month follow-up outcome will be calculated no earlier than June 2020. The other two, readily after the publication of this SAP, but before unblinding of analysists.

6.8.1.1 Calculation of the outcome: specific measurement and units (and transformation, where applicable)

Time from baseline to RTW is defined af the number of weeks from randomization date, to stable return to work. Stable return to work is defined as 4 weeks consecutively in work, i.e. with no sick leave benefit those 4 weeks in the Dream register, and a so-called "branch code" in at least some of this 4 week period (benefit codes are week-based, branch codes are month based, and hence a period of 4 weeks may represent only one month, or overlap a two month period; in the latter case, return to work will be attained if at least one of these registrations contains a branch code; a branch code means that the individual received salary from an employer in this period). Time of event is first day of the four weeks.

These events will define censoring: 1) moving out of the country, 2) death, 3) public retirement pension (Da.: "Folkepension"), and 4) voluntary early retirement scheme (Danish: "Efterløn").

At randomization all participants are, according to inclusion criteria, on sick-leave from employment or vacancy. Some participants might be on sick-leave from an employment in a *flexjob*[7], and hence receiving *flexjob benefit* during employment. This benefit is changed to *flexjob sick-leave benefit* similar to regular sick leave benefit for participants not granted flexjob benefit prior to randomization. In these cases (of participants granted flexjob benefit prior to randomization) RTW is defined as either not receiving flexjob sick-leave benefit for four consecutive weeks, along with a registered branch code as above mentioned (or alternatively not receiving flexjob benefit, but an ordinary salary indicated by a branch code during those four weeks).

[7] "Flexjob" is one of the Danish benefit schemes; it is a subsidy granted those with a chronic reduced work capacity

For participants, who at baseline are on sick-leave from vacancy (but not receiving flexjob benefit), RTW can both be defined as above mentioned (four consecutive weeks without sick leave benefits and a branch code during those four weeks) or receiving flexjob benefit for four consecutive weeks and a branch code during those four weeks.

6.8.1.2 Specific analysis method and result presentation

Comparisons of RTW time will be calculated as hazard rate ratios between groups (and corresponding 98,3%CI), using a Cox-regression model.

Kaplan-Meier curves will be presented to illustrate the cumulative incidence of first stable return to work event in each trial-arm.

6.8.1.3 Covariate adjustment

Only for stratification variables, see *6.1 "Covariate adjustment in general"*.

6.8.1.4 Statistical method assumption control

Assumptions for the proportional hazards (~Cox-) regression model are proportional hazards; this will be controlled performing af Schoenfeld (SF) test for residuals and visual inspection.

6.8.1.5 Alternative analysis method in case of assumption fail

If the SF test is positive ($p<0,05$), the analysis will we performed adjusted for the interaction between time and treatment group allocation. If SF test hereafter is still positive, the analysis will instead be adjusted for the interaction between *quadratic* time ($time^2$) and treatment group allocation. If SF test hereafter is still positive, the analysis will instead be adjusted for the interaction between *log*(time) and treatment group allocation. If SF test hereafter is still positive, the analysis with the highest p-value will be reported.

6.8.1.6 Sensitivity analyses

See *"6.2.2 Sensitivity analyses for register data based outcomes"*.

6.8.1.7 Reporting and statistical methods to handle missing data

On RWT-outcomes we expect *no missing data*, due to the nature of the Dream Register. Missing data will only be in case of a participant dying or moving out of Denmark. We consider these events to be so rare in our data that we will handle such missing data as *missing completely at random*. Thus, no imputation or other correction is necessary. We will report proportion of data missing.

We will report number of censored participants per treatment group.

6.8.2 Proportion in ordinary work at 12-month follow-up (secondary outcome) and 24-month follow-up (exploratory outcome) (outcome #2 and #14)

6.8.2.1 Calculation of the outcome: specific measurement and units (and transformation, where applicable)

This outcome is calculated as the share of the treatment allocation group that on the time of follow-up was in stable RTW (≥ 4 weeks). Stable RTW if defined exactly as in the primary outcome, see 6.8.1.1.

6.8.2.2 Specific analysis method and result presentation

Pairwise odds ratios will be calculated using logistic regression.

In addition to the presentation of odds ratios for tests at 12-month follow-up and 24-month follow-up, graphs are presented with the proportions in stable work at each week (week 1-52 for 12-month follow-up and week 1-104 for 24-month follow-up) for each of the three trial-arms. No statistical test will be performed for differences at week 1-51 or week 53-103. These curves are explorative, descriptive analyses.

6.8.2.3 Covariate adjustment

Only for stratification variables, see *6.1 "Covariate adjustment in general"*.

6.8.2.4 Statistical method assumption control

The assumptions of the model are assumed to be acceptable, due to large sample, binary outcome, categorical independent variable.

6.8.2.5 Alternative analysis method in case of assumption fail

No alternative methods are planned, since assumptions are assumed to hold.

6.8.2.6 Sensitivity analyses

See *"6.2.2 Sensitivity analyses for register data based outcomes"*.

6.8.2.7 Reporting and statistical methods to handle missing data

Same as 6.8.1.7.

6.8.3 All self-reported, numerical outcomes, at 6-, 12-, and 24-month follow-up at (secondary outcomes ##4-7 and predefined exploratory outcomes ##16-48)

6.8.3.1 *Calculation of the outcome: specific measurement and units (and transformation, where applicable)*

All outcomes are calculated as the sum of scores on the respective measurement scales.

All 6-month follow-up outcome analyses are calculating using baseline and 6-month follow-up observations.

All 12-month follow-up outcome analyses are calculating using baseline and 6- and 12-month follow-up observations.

All 24-month follow-up outcome analyses are calculating using baseline and 6-, 12-, and 24-month follow-up observations.

6.8.3.2 *Specific analysis method and result presentation*

Linear mixed-effects model with unstructured covariance. Results will be presented in pairwise group differences between outcomes, from the estimated marginal means from the model, and the confidence intervals of these differences.

6.8.3.3 *Covariate adjustment*

Only for stratification variables, see *6.1 "Covariate adjustment in general*".

6.8.3.4 *Statistical method assumption control*

Assumption: normal distribution of scores. Control: Visual inspection by plotting the score residuals.

Assumption: normal distribution of individuals' score differences between baseline and follow-up. Control: Visual inspection by plotting the score difference residuals.

Assumption: Equality and homogeneity of variance. Control: Breusch Pagan test and Bartlett's test are used to identify violations of these assumptions.

6.8.3.5 *Alternative analysis method in case of assumption fail*

In case of positive tests or visual inspections a robust variance estimator is used to correct standard errors.

6.8.3.6 *Sensitivity analyses*

See "*6.2.1 Sensitivity analyses for questionnaire based, self-reported data outcome*".

6.8.3.7 *Reporting and statistical methods to handle missing data*

Proportion and amount of missing data per outcome variable per follow-up event per treatment group will be reported.

To handle missing data, 100 multiple imputations will be performed, using following variables: stratification variables: diagnosis, municipality, employment status; age; gender; time to stable RTW; psychometric variables at baseline and all follow-up at outcome time: BDI, BAI, WSAS and PSS.

6.8.4 Weeks of work from baseline to 12- and 24-month follow-up (outcomes ##10-14)

6.8.4.1 *Calculation of the outcome: specific measurement and units (and transformation, where applicable)*

From baseline to follow-up, the number of weeks in work per participant is calculated. A week is noted as being in work, if no sick leave benefit has been received, *and* if a branch code is registered in the month of that week (branch codes are registered on monthly basis, if an individual has received salary from an ordinary job during that month).

For participants receiving flexible job benefit prior to randomization, and participants on sick leave from vacancy, the same principles apply, as described in 6.8.1.1, in the section "*Time to return to work-outcomes (outcomes #1, #3 and #8)*".

At 24-month follow-up, this analysis is conducted with three variations each applying a different definition of return to work stability as sensitivity analyses. Whereas the first analysis uses the definition of stability from the primary outcome (minimum four weeks see section 6.8.1.1), these sensitivity analyses are conducted with a more conservative approach where stable return to work is defined as minimum 4, 8 and 12 weeks in work respectively.

6.8.4.2 Specific analysis method and result presentation

Severely skewed data is expected for this outcome, why a robust Poisson regression model will be used to test the differences between groups.

6.8.4.3 Covariate adjustment

Only for stratification variables, see *6.1 "Covariate adjustment in general"*.

6.8.4.4 Statistical method assumption control

Assumption: Poisson distribution. Control: X^2 goodness-of-fit test.

6.8.4.5 Alternative analysis method in case of assumption fail

If X^2 goodness-of-fit test is significant, negative binomial regression model will be used instead. If X^2 goodness-of-fit test is significant for this distribution, zero inflated poisson regression will be used.

6.8.4.6 Sensitivity analyses

See *"6.2.2 Sensitivity analyses for register data based outcomes"*.

6.8.4.7 Reporting and statistical methods to handle missing data

See 6.8.1.7

6.8.5 At 24 months: Number of recurrent sick leaves at 24-month follow-up (outcome #15)

6.8.5.1 Calculation of the outcome: specific measurement and units (and transformation, where applicable)

For each group, the number of persons who have experienced the event 'stable return to work' and followingly experienced the event 'recurring sick leave' is calculated. Recurring sick leave is defined as the first sick leave period starting with the fist week of receiving sickness benefit after a period of stable return to work as defined in paragraph 6.8.1.1.

6.8.5.2 Specific analysis method and result presentation

Only descriptive statistics will be performed for this outcome and no differences between groups will be tested. For each group, the number of persons who have experienced stable return to work and the number of persons who have experienced recurrent sick leave is presented.

6.8.6 Harm measures at 12-, and 24-month follow-up (outcome #49-64)

6.8.6.1 Calculation of the outcome: specific measurement and units (and transformation, where applicable)

For each group, the number of persons who have experienced the harmful event is calculated.

6.8.6.2 Specific analysis method and result presentation

Only descriptive statistics will be performed for this outcome and no differences between groups will be tested.

7 REFERENCES

1. Gamble C, Krishan A, Stocken D, et al. Guidelines for the Content of Statistical Analysis Plans in Clinical Trials. *JAMA*. 2017;318(23):2337. doi:10.1001/jama.2017.18556

2. Poulsen R, Fisker J, Hoff A, Hjorthøj C, Eplov LF. Integrated mental health care and vocational rehabilitation to improve return to work rates for people on sick leave because of depression and anxiety (the Danish IBBIS trial): study protocol for a randomized controlled trial. *Trials*. 2017;18:578. doi:10.1186/s13063-017-2272-1

3. Poulsen R, Fisker J, Hoff A, Hjorthøj C, Eplov LF. Integrated mental health care and vocational rehabilitation to improve return to work rates for people on sick leave because of exhaustion disorder, adjustment disorder, and distress (the Danish IBBIS trial): Study protocol for a randomized controlled tri. *Trials*. 2017;18(1). doi:10.1186/s13063-017-2273-0

4. Nielsen MBD, Madsen IEH, Bültmann U, Christensen U, Diderichsen F, Rugulies R. Predictors of return to work in employees sick-listed with mental health problems: Findings from a longitudinal study. *Eur J Public Health*. 2011;21:806-811. doi:10.1093/eurpub/ckq171

5. Oostrom SH Van, Mechelen W Van, Terluin B, Vet HCW De, Knol DL, Anema JR. A workplace intervention for sick-listed employees with distress: results of a randomised controlled trial. 2010. doi:10.1136/oem.2009.050849

6. Vlasveld MC, van der Feltz-Cornelis CM, Ader HJ, et al. Collaborative care for sick-listed workers with major depressive disorder: a randomised controlled trial from the Netherlands Depression Initiative aimed at return to work and depressive symptoms. *Occup Environ Med*. 2013;70(4):223-230. doi:10.1136/oemed-2012-100793

7. Lagerveld SE, Blonk RWB, Brenninkmeijer V, Wijngaards-de Meij L, Schaufeli WB. Work-focused treatment of common mental disorders and return to work: A comparative outcome study. *J Occup Health Psychol*. 2012;17(2):220-234. doi:10.1037/a0027049

8. Beck AT, Brown G, Epstein N, Steer RA. An Inventory for Measuring Clinical Anxiety: Psychometric Properties. *J Consult Clin Psychol*. 1988;56(6):893-897. doi:10.1037/0022-006X.56.6.893

9. Beck AT, Steer RA, Carbin MG. Psychometric properties of the Beck Depression Inventory: Twenty-five years of evaluation. *Clin Psychol Rev*. 1988;8(1):77-100. doi:10.1016/0272-7358(88)90050-5

10. Cohen S, Kamarck T, Mermelstein R. A global measure of perceived stress. *J Health Soc Behav*. 1983;24(4):385-396. doi:10.2307/2136404

11. Mundt JC, Marks IM, Shear MK, Greist JH. The Work and Social Adjustment Scale: A simple measure of impairment in functioning. *Br J Psychiatry*. 2002;180(MAY):461-464. doi:10.1192/bjp.180.5.461

12. Terluin B, van Marwijk HWJ, Adèr HJ, et al. The Four-Dimensional Symptom Questionnaire (4DSQ): a validation study of a multidimensional self-report questionnaire to assess distress, depression, anxiety and somatization. *BMC Psychiatry*. 2006;6:34. doi:10.1186/1471-244X-6-34

13. Saboonchi F, Perski A, Grossi G. Validation of Karolinska Exhaustion Scale: Psychometric properties of a measure of exhaustion syndrome. *Scand J Caring Sci*. 2013;27(4):1010-1017. doi:10.1111/j.1471-6712.2012.01089.x

14. Brazier J, Roberts J, Tsuchiya A, Busschbach J. A comparison of the EQ-5D and SF-6D across seven patient groups. *Health Econ*. 2004;13(9):873-884. doi:10.1002/hec.866

15. Burckhardt CS, Anderson KL. The Quality of Life Scale (QOLS): reliability, validity, and utilization. *Health Qual Life Outcomes*. 2003;1(1):60. doi:10.1186/1477-7525-1-60

16. Moss-Morris R, Weinman J, Petrie KJ, Horne R, Cameron LD, Buick D. The revised Illness Perception Questionnaire (IPQ-R). *Psychol Health*. 2002;17(1):1-16. doi:10.1080/08870440290001494

17. Lagerveld SE, Blonk RWB, Brenninkmeijer V, Schaufeli WB. Return to work among employees with mental health problems: Development and validation of a self-efficacy questionnaire. *Work Stress*.

2010;24(4):359-375. doi:10.1080/02678373.2010.532644

18. Schwarzer R, Jerusalem M. Generalized Self-Efficacy scale. In: Wright S, Johnston M, eds. *Measures in Health Psychology: A User's Portfolio. Causal and Control Beliefs*. Windsor, England; 1995:35-37.

19. Attkisson C, Zwick R. The Client Satisfaction Questionnaire: Psychometric properties and correlations with service utilization and psychotherapy outcome. *Eval Program Plann*. 1982;5:233-237.

20. Koopman C, Pelletier KR, Murray JF, et al. Stanford presenteeism scale: health status and employee productivity. *J Occup Environ Med*. 2002;44(1):14-20.

21. EuroQol Group. EuroQol–a new facility for the measurement of healthrelated quality of life. The EuroQol Group. *Health Policy (New York)*. 1990;16(3):199-208. doi:10.1016/0168-8510(90)90421-9

22. Weinman J, Petrie KJ, Moss-Morris R, Horne R. The illness perception questionnaire: A new method for assessing the cognitive representation of illness. *Psychol Heal*. 1996;11(3):431-445. doi:10.1080/08870449608400270

23. Koopman C, Pelletier KR, Murray JF, et al. Stanford presenteeism scale: health status and employee productivity. *J Occup Environ Med*. 2002;44(1):14-20. http://www.ncbi.nlm.nih.gov/pubmed/11802460.

Supplement 5: Self-report outcomes results

	Outcome domain	Outcome (E=explorative)		Group means (SDs)			Group comparisons								
							SAU - MHC			SAU - INT			MHC - INT		
				INT	MHC	SAU	Diff.	p	98.3 % CI	Diff.	p	98.3 % CI	Diff.	p	98.3 % CI
24-month follow-up	Symptoms	BAI	E	11,73 (9,04)	12,34 (9,32)	11,69 (9,17)	0,27	0,784	(-2,12–2,66)	0,77	0,429	(-1,56–3,09)	0,51	0,582	(-1,72–2,74)
		BDI	E	11,21 (11,3)	11,41 (10,99)	10,9 (10,38)	-0,05	0,961	(-2,72–2,61)	0,25	0,819	(-2,37–2,87)	0,32	0,765	(-2,21–2,85)
		PSS	E	15,55 (8,37)	15,7 (7,2)	14,97 (7,59)	-0,13	0,867	(-2–1,74)	0,06	0,937	(-1,84–1,97)	0,18	0,81	(-1,62–1,98)
		KES	E	57,78 (19,77)	58,78 (19,58)	58,25 (18,42)	-0,08	0,966	(-4,35–4,2)	0,41	0,819	(-3,9–4,73)	0,48	0,786	(-3,75–4,71)
		4DSQ soma.	E	9,39 (6,96)	9,43 (6,2)	9,73 (6,45)	0,32	0,645	(-1,35–1,99)	0,47	0,497	(-1,18–2,11)	0,15	0,82	(-1,47–1,78)
		4DSQ-distr.	E	12,21 (8,33)	13,12 (8,25)	12,25 (8,51)	-0,60	0,457	(-2,55–1,34)	0,07	0,927	(-1,88–2,03)	0,67	0,391	(-1,2–2,53)
		4DSQ-anx.	E	3,45 (4,36)	4,44 (5,2)	3,48 (4,54)	-0,33	0,538	(-1,61–0,95)	0,22	0,673	(-1,01–1,45)	0,55	0,28	(-0,67–1,77)
		4DSQ-depr.	E	1,62 (2,74)	1,81 (2,81)	1,72 (2,87)	-0,01	0,964	(-0,77–0,74)	0,10	0,753	(-0,65–0,84)	0,11	0,706	(-0,59–0,8)
	Functioning	WSAS	E	11,5 (10,57)	12,01 (10,41)	11,2 (9,83)	-0,50	0,652	(-3,13–2,14)	0,14	0,893	(-2,36–2,64)	0,64	0,536	(-1,83–3,1)
	Presenteeism	SPS	E	18,61 (4,29)	19,23 (3,78)	19,08 (3,39)	0,78	0,306	(-1,03–2,59)	1,04	0,123	(-0,56–2,63)	0,31	0,677	(-1,47–2,08)
	Self-efficacy	IPQ	E	17,49 (4,35)	16,77 (4,11)	16,94 (4,13)	0,18	0,685	(-0,87–1,22)	-0,42	0,323	(-1,43–0,59)	-0,58	0,169	(-1,58–0,43)
		GSE	E	28,13 (6,84)	28 (7,27)	29,15 (7,01)	0,51	0,493	(-1,28–2,3)	0,33	0,645	(-1,39–2,05)	-0,18	0,8	(-1,88–1,53)
	Life quality	QoLs	E	73,08 (16,69)	72,21 (17,2)	72,93 (15,46)	0,35	0,825	(-3,46–4,17)	0,26	0,867	(-3,41–3,93)	-0,09	0,955	(-3,75–3,57)
		EQ5	E	0,8 (0,18)	0,79 (0,16)	0,81 (0,15)	0,01	0,643	(-0,03–0,05)	0,01	0,718	(-0,03–0,05)	0,00	0,917	(-0,04–0,04)

Self-report data outcomes.
Group means are values after imputation. Abbreviations: Diff.: Difference (estimated marginal means from Linear Mixed-effect models); SD: Standard Deviation; MHC: Mental healthcare; SAU: Service as usual; INT: Integrated intervention; BDI: Bech Depression Inventory; BAI: Bech Anxiety Inventory; PSS: Perceived Stress Scale; WSAS: Work and Social Adjustment Scale; 4DSQ: Four Dimensional Questionnaire; KEDS: Karolinska exhaustion disorder scale; IPQ: Illness Perception Questionnaire; EQ5DL: Health related quality of life; QoLs: Quality of Life Scale; RTW-SE: Return to work-self efficacy; SPS: Stanford Presenteeism scale; GSE: Generalized Self-Efficacy Scale; E: Exploratory (outcome)

Supplement 6: Subgroup analyses

The following pages contains the results from the subgroup analyses of the IBBIS Randomized Trial for anxiety and depression

Supplement: Subgroup analyses report

Abbrev.: IBBIS: Integreret Behandlings- og BeskaeftigelsesIndsats til Sygedagpengemodtagere (English translation: Integrated Health Care and Vocational Rehabilitation for Sick Leave Benefit Recipients); HR: Hazard Ratio; INT: Integrated intervention; MHC: Mental health care; RCT: Randomized controlled trial; RTW: Return to work; SAU: Service as usual; OR: Odds Ratio; RR: Relative risk; FUn: n-month follow-up

Table legends: "Est.": Estimate "p": p-value "low/high CI": Low vs. high bounds of the 98.3% Confidence Interval of the estimate.

Subgroup abbrev.:

The following abbreviations reflects subgroups including only participants with following characteristics:

- "depr": Depression,
- "anx": Anxiety,
- "emp": Employment (at baseline),
- "vac": Unemployed (at baseline),
- "north": Team North,
- "city": Team City (Copenhagen),
- "first": All participants randomized in the first temporal half of the RCT,
- "last": All participants randomized in the last temporal half of the RCT,
- "interaction": Analyses adjusted for the diagnosis x treatment arm interaction
- "intempl": Analyses adjusted for the employment status (employed vs uempl.) x treatment arm interaction
- "intteam": Analyses adjusted for the intervention-team (Team North vs. City) x treatment arm interaction
- "inttemp": Analyses adjusted for the time (first vs. last trial period half) x treatment arm interaction

Legend: *: 0,05 > p > 0,016667 | **: p < 0,016667

This report chapter systematically displays all results from the IBBIS RCT trial covering diagnoses of anxiety and depression.

Analyses regarding: >depr<

Proportion over time-curve at 24-month follow-up regarding: depr<

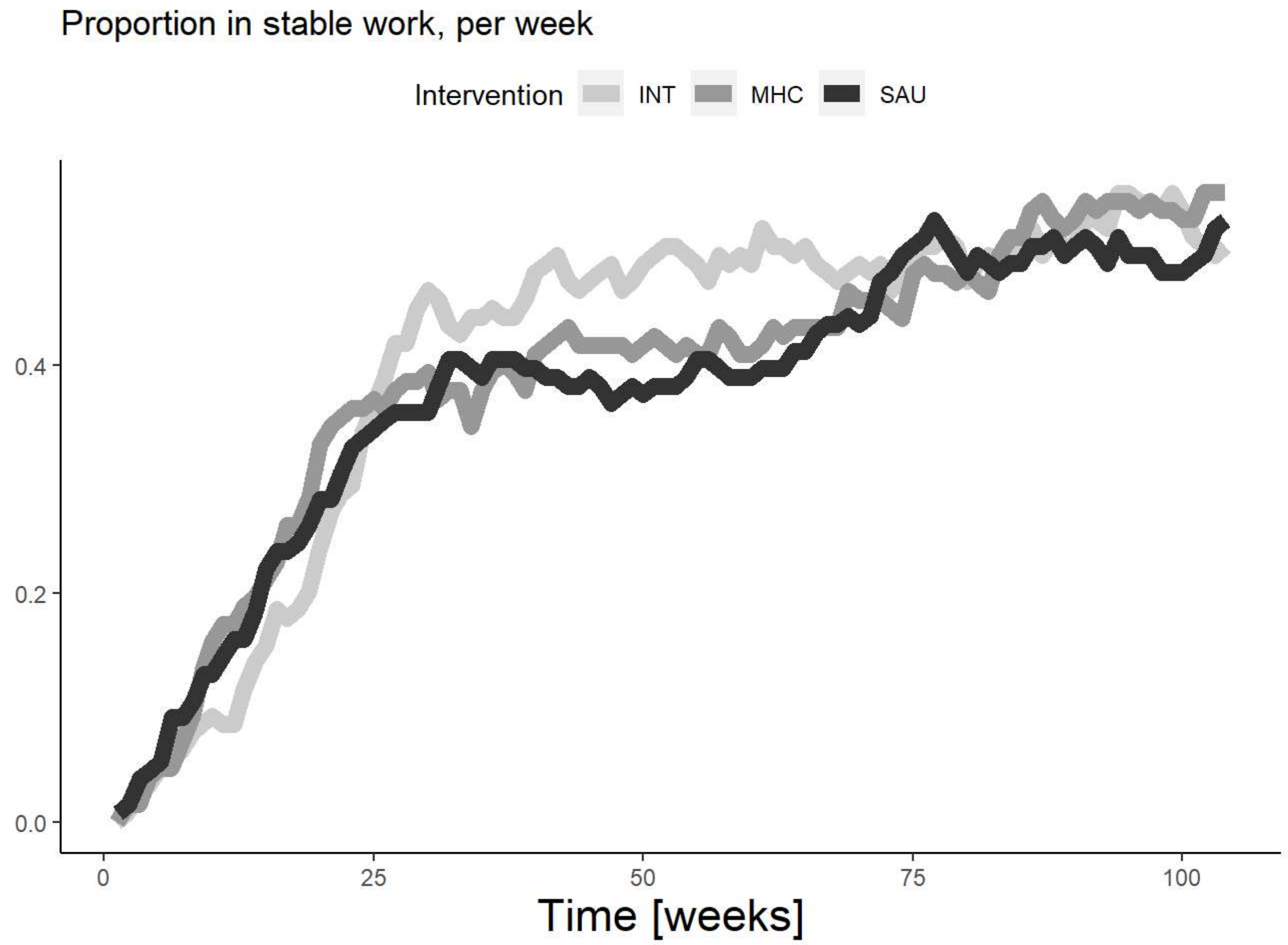

Vocational outcomes at 24-month follow-up from subgroup >depr< in RCT1

	Group values			SAU-MHC				SAU-INT				MHC-INT			
	**INT**	**MHC**	**SAU**	**Est.**	**p**	**low CI**	**high CI**	**Est.**	**p**	**low CI**	**high CI**	**Est.**	**p**	**low CI**	**high CI**
RTW, FU24	30	36	41	0.96	0.77	0.67	1.37	0.86	0.29	0.6	1.22	0.91	0.52	0.64	1.29
PROP, FU24	53.3	55.6	53.5	0.92	0.74	0.49	1.71	1.01	0.98	0.54	1.88	1.09	0.74	0.58	2.04
WEEKS, FU24	44.6	41.3	39.9	0.96	0.72	0.75	1.24	0.9	0.3	0.71	1.14	0.93	0.48	0.74	1.18

Kaplan Meier-curve at 24-month follow-up regarding: depr

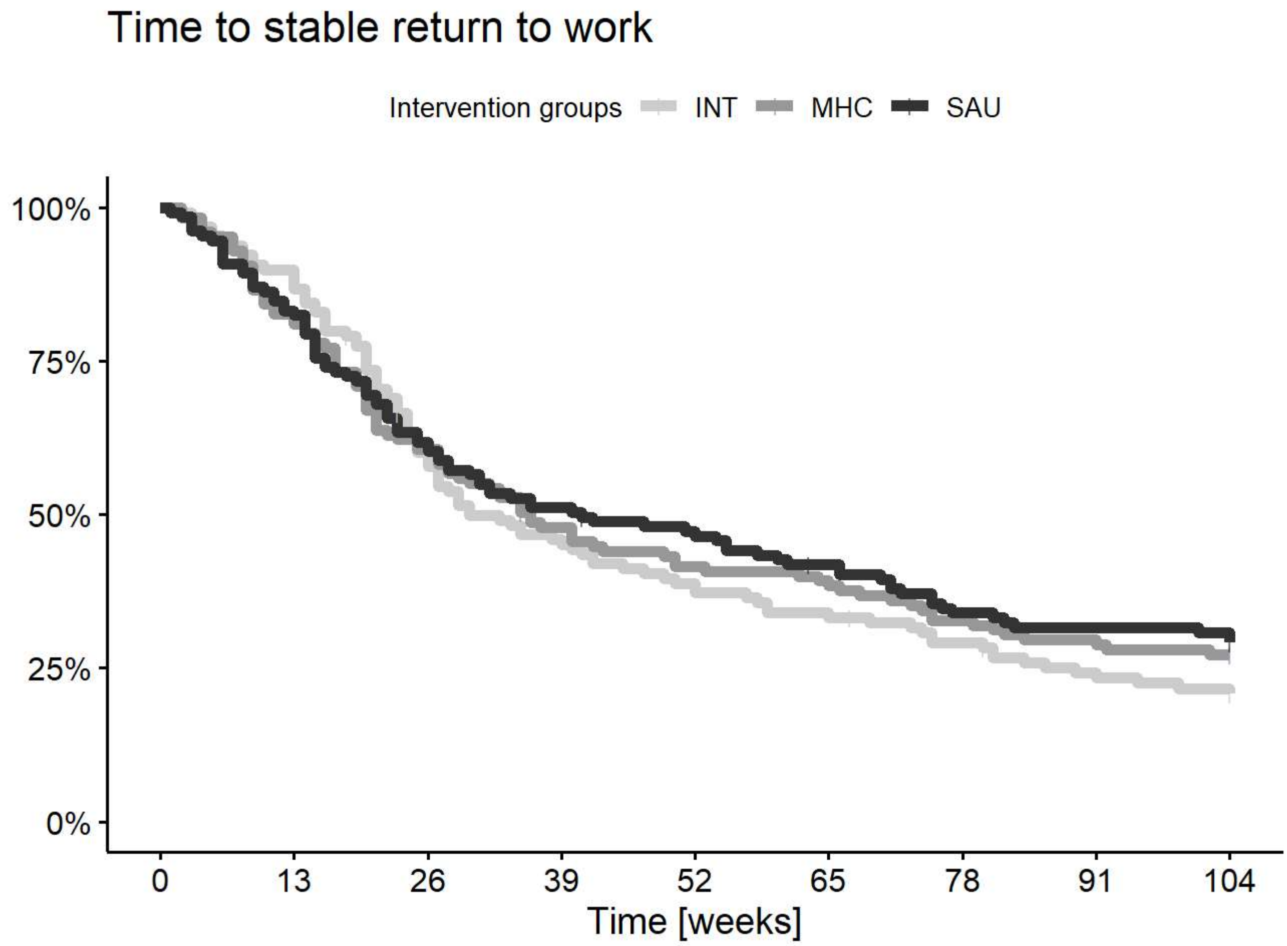

Self-reported outcomes at 24-month follow-up regarding: depr

	Group values						SAU-MHC				SAU-INT				MHC-INT			
	INT	(SD)	MHC	(SD)	SAU	(SD)	Est.	p	low CI	high CI	Est.	p	low CI	high CI	Est.	p	low CI	high CI
BAI [FU24]	12.31	(9.92)	10.78	(9.68)	11.39	(8.81)	1.18	0.34	-1.76	4.11	0.46	0.7	-2.47	3.4	-0.7	0.56	-3.55	2.15
BDI [FU24]	12.56	(11.39)	11	(11.67)	11.87	(10.73)	1.13	0.43	-2.29	4.55	0.2	0.89	-3.19	3.6	-0.85	0.54	-4.14	2.44
PSS [FU24]	16.05	(8.73)	14.9	(7.69)	15.27	(7.48)	0.55	0.58	-1.8	2.9	-0.04	0.97	-2.47	2.39	-0.59	0.54	-2.93	1.74
KES [FU24]	60.49	(19.77)	57.35	(20.01)	59.14	(18.23)	1.7	0.45	-3.65	7.04	-0.65	0.77	-5.96	4.66	-2.33	0.31	-7.78	3.12
DSQ-som. [FU24]	10.25	(7.46)	8.85	(6.36)	9.34	(6.04)	0.49	0.57	-1.61	2.59	-0.11	0.9	-2.14	1.93	-0.58	0.51	-2.69	1.53
DSQ-distr. [FU24]	13.09	(8.59)	12.79	(8.67)	12.63	(8.63)	0.05	0.96	-2.4	2.5	-0.13	0.9	-2.53	2.28	-0.16	0.87	-2.52	2.2
DSQ-anx. [FU24]	3.62	(4.56)	3.48	(5.09)	3.14	(4.44)	0.21	0.75	-1.37	1.79	0.03	0.96	-1.53	1.59	-0.18	0.78	-1.7	1.35
DSQ-depr. [FU24]	1.68	(2.76)	1.81	(2.7)	1.87	(3)	0.18	0.65	-0.79	1.15	0.21	0.6	-0.75	1.17	0.04	0.91	-0.85	0.94
WSAS [FU24]	12.73	(10.99)	11.17	(10.71)	12.51	(10.18)	1	0.47	-2.32	4.32	0.2	0.88	-2.99	3.39	-0.77	0.56	-3.93	2.39
SPS [FU24]	18.66	(4.23)	18.74	(4.4)	19.38	(2.89)	0.77	0.31	-1.02	2.57	1.05	0.12	-0.54	2.63	0.31	0.67	-1.47	2.09
IPQ [FU24]	16.9	(4.7)	16.65	(4.43)	16.64	(4.31)	0.07	0.9	-1.25	1.38	-0.19	0.73	-1.49	1.12	-0.24	0.66	-1.55	1.07
GSS [FU24]	27.73	(6.88)	28.42	(7.47)	28.78	(6.95)	0.09	0.93	-2.13	2.3	0.45	0.63	-1.75	2.64	0.33	0.72	-1.87	2.53
QoLs [FU24]	71.6	(16.87)	72.59	(17.23)	71.41	(14.85)	-0.85	0.67	-5.58	3.88	0.36	0.85	-4.3	5.02	1.14	0.56	-3.53	5.8
EQ5 [FU24]	0.78	(0.17)	0.8	(0.18)	0.8	(0.15)	-0.01	0.78	-0.05	0.04	0.01	0.6	-0.04	0.06	0.02	0.42	-0.03	0.07

Analyses regarding: >anx<

Proportion over time-curve at 24-month follow-up regarding: anx<

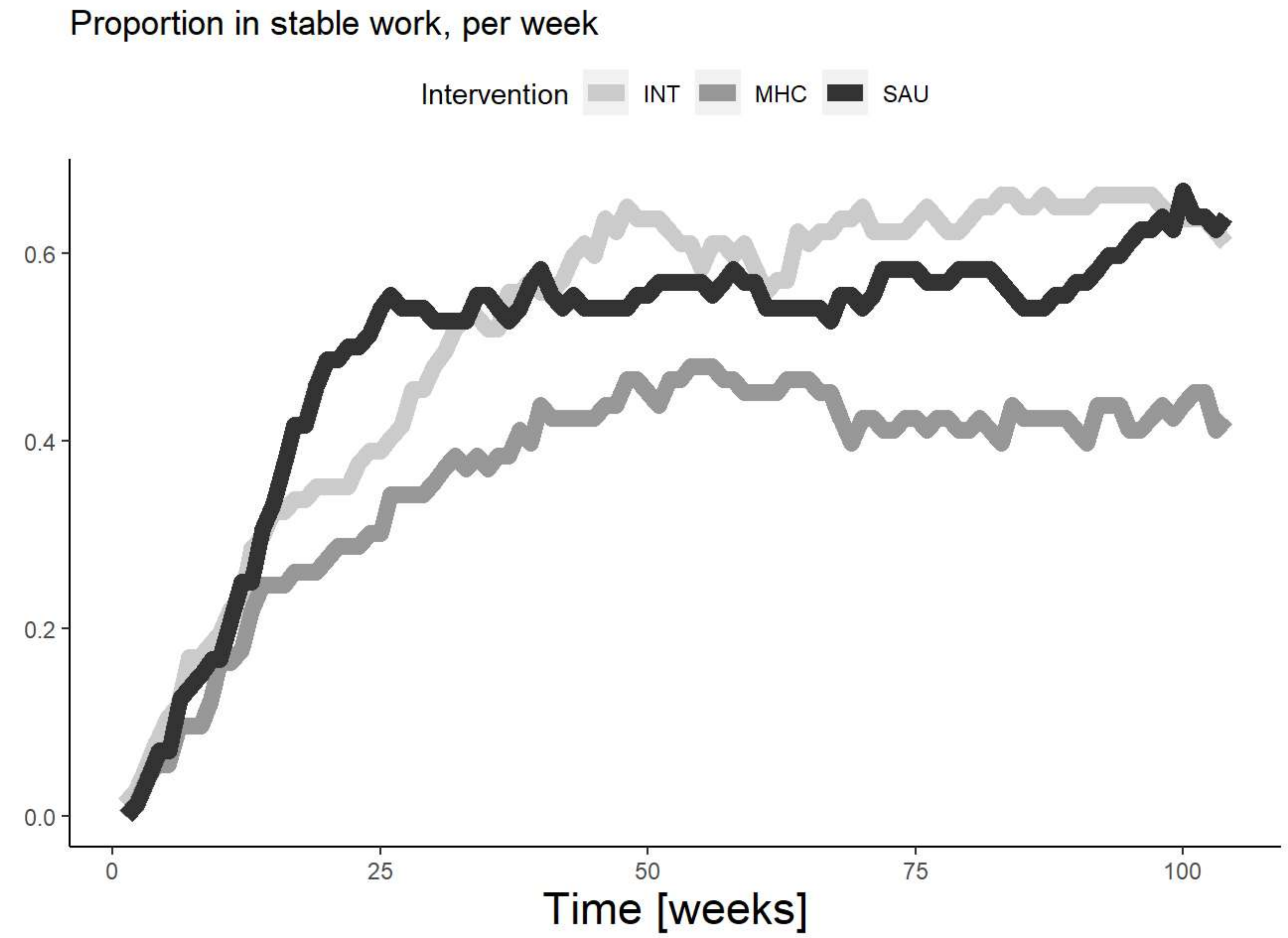

Vocational outcomes at 24-month follow-up from subgroup >anx< in RCT1

	Group values			SAU-MHC				SAU-INT				MHC-INT			
	**INT**	**MHC**	**SAU**	**Est.**	**p**	**low CI**	**high CI**	**Est.**	**p**	**low CI**	**high CI**	**Est.**	**p**	**low CI**	**high CI**
RTW, FU24	30.0	40.0	20.0	1.65	**0.0128	1.01	2.68	1.04	0.85	0.66	1.62	0.62	*0.0177	0.39	1.01
PROP, FU24	62.7	43.1	64.8	2.57	**0.0074	1.09	6.03	1.07	0.84	0.46	2.49	0.42	**0.0143	0.18	0.99
WEEKS, FU24	55.2	38.9	52.8	1.37	*0.0244	0.98	1.93	0.96	0.70	0.74	1.25	0.70	**0.0084	0.51	0.97

Kaplan Meier-curve at 24-month follow-up regarding: anx

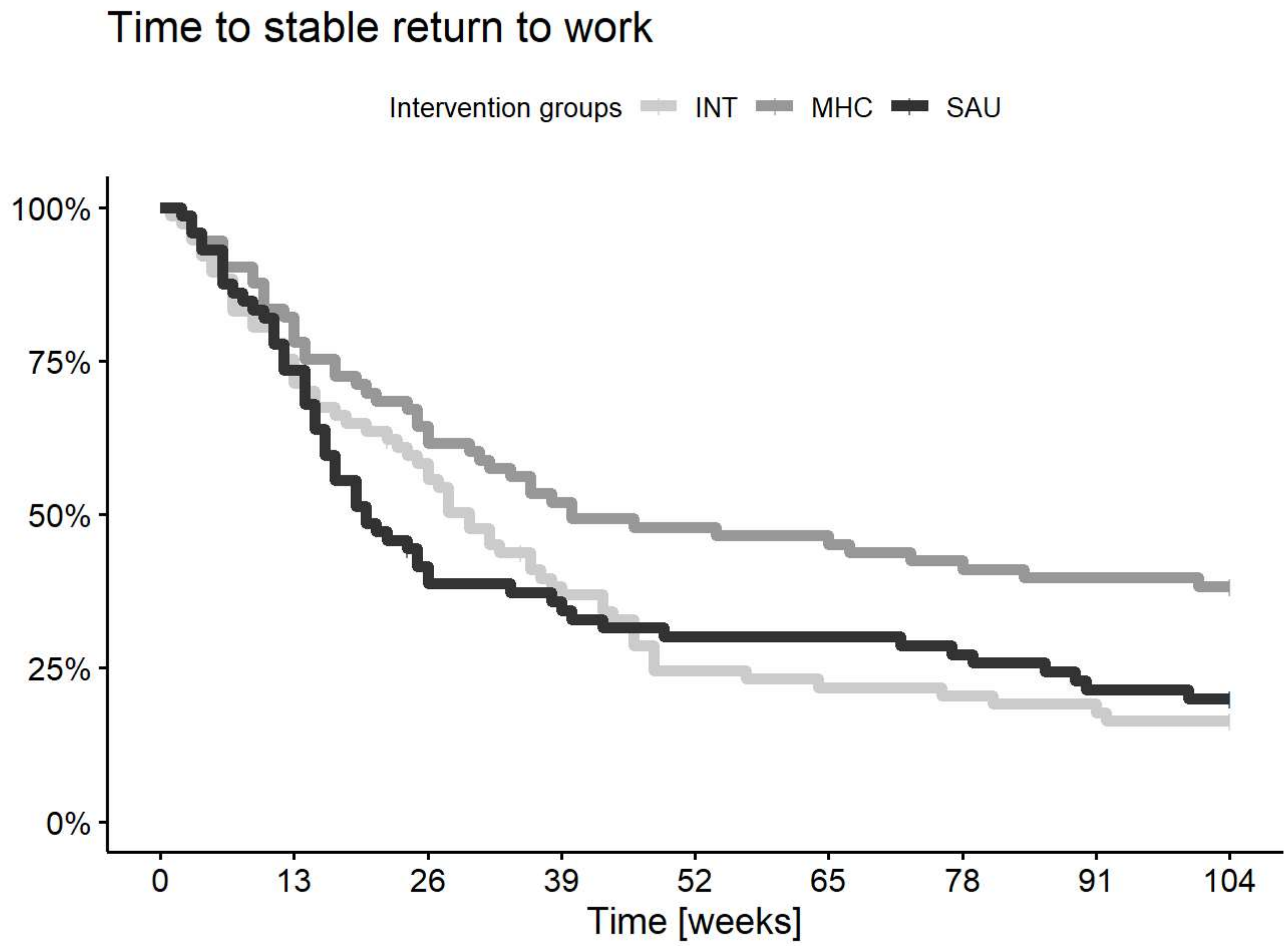

Self-reported outcomes at 24-month follow-up regarding: anx

	Group values						SAU-MHC				SAU-INT				MHC-INT			
	INT	(SD)	MHC	(SD)	SAU	(SD)	Est.	p	low CI	high CI	Est.	p	low CI	high CI	Est.	p	low CI	high CI
BAI [FU24]	10.84	(7.46)	14.67	(8.29)	12.28	(9.91)	-1.14	0.48	-5.06	2.77	1.46	0.34	-2.23	5.14	2.63	0.07	-0.83	6.09
BDI [FU24]	9.1	(10.91)	12.03	(9.97)	9	(9.49)	-2.04	0.24	-6.22	2.14	0.4	0.81	-3.53	4.33	2.44	0.12	-1.36	6.24
PSS [FU24]	14.79	(7.81)	16.89	(6.28)	14.38	(7.85)	-1.29	0.31	-4.34	1.77	0.28	0.82	-2.7	3.26	1.56	0.19	-1.27	4.38
KES [FU24]	53.62	(19.22)	60.93	(18.89)	56.51	(18.9)	-2.96	0.32	-10.08	4.16	2.49	0.41	-4.69	9.67	5.52	*0.0492	-1.21	12.25
DSQ-som. [FU24]	8.04	(5.89)	10.29	(5.92)	10.47	(7.2)	0.16	0.89	-2.51	2.82	1.55	0.17	-1.16	4.27	1.47	0.16	-1.04	3.97
DSQ-distr. [FU24]	10.84	(7.77)	13.61	(7.62)	11.53	(8.35)	-1.62	0.21	-4.73	1.5	0.51	0.7	-2.7	3.72	2.18	0.08	-0.78	5.13
DSQ-anx. [FU24]	3.18	(4.05)	5.89	(5.05)	4.12	(4.72)	-1.19	0.18	-3.32	0.94	0.59	0.48	-1.41	2.58	1.83	*0.0288	-0.18	3.83
DSQ-depr. [FU24]	1.52	(2.74)	1.8	(2.99)	1.45	(2.62)	-0.3	0.52	-1.42	0.82	-0.07	0.88	-1.18	1.05	0.26	0.57	-0.81	1.33
WSAS [FU24]	9.6	(9.68)	13.27	(9.9)	8.68	(8.7)	-3.09	0.08	-7.28	1.11	0.08	0.96	-3.74	3.91	3.14	0.05	-0.71	6.99
SPS [FU24]	18.54	(4.41)	20.05	(2.25)	18.5	(4.18)	0.77	0.31	-1.02	2.57	1.05	0.12	-0.54	2.63	0.31	0.67	-1.47	2.09
IPQ [FU24]	18.42	(3.57)	16.94	(3.57)	17.51	(3.73)	0.33	0.64	-1.4	2.07	-0.87	0.2	-2.51	0.76	-1.19	0.07	-2.77	0.38
GSS [FU24]	28.76	(6.8)	27.35	(6.95)	29.87	(7.15)	1.16	0.34	-1.76	4.08	0.09	0.94	-2.69	2.87	-1.12	0.32	-3.81	1.58
QoLs [FU24]	75.38	(16.3)	71.62	(17.3)	75.9	(16.38)	2.31	0.38	-4.04	8.67	0.05	0.99	-5.88	5.97	-2.39	0.32	-8.2	3.41
EQ5 [FU24]	0.83	(0.19)	0.78	(0.15)	0.83	(0.14)	0.03	0.29	-0.04	0.09	-0.01	0.84	-0.07	0.06	-0.03	0.19	-0.1	0.03

Analyses regarding: >interaction<

Proportion over time-curve at 24-month follow-up regarding: interaction<

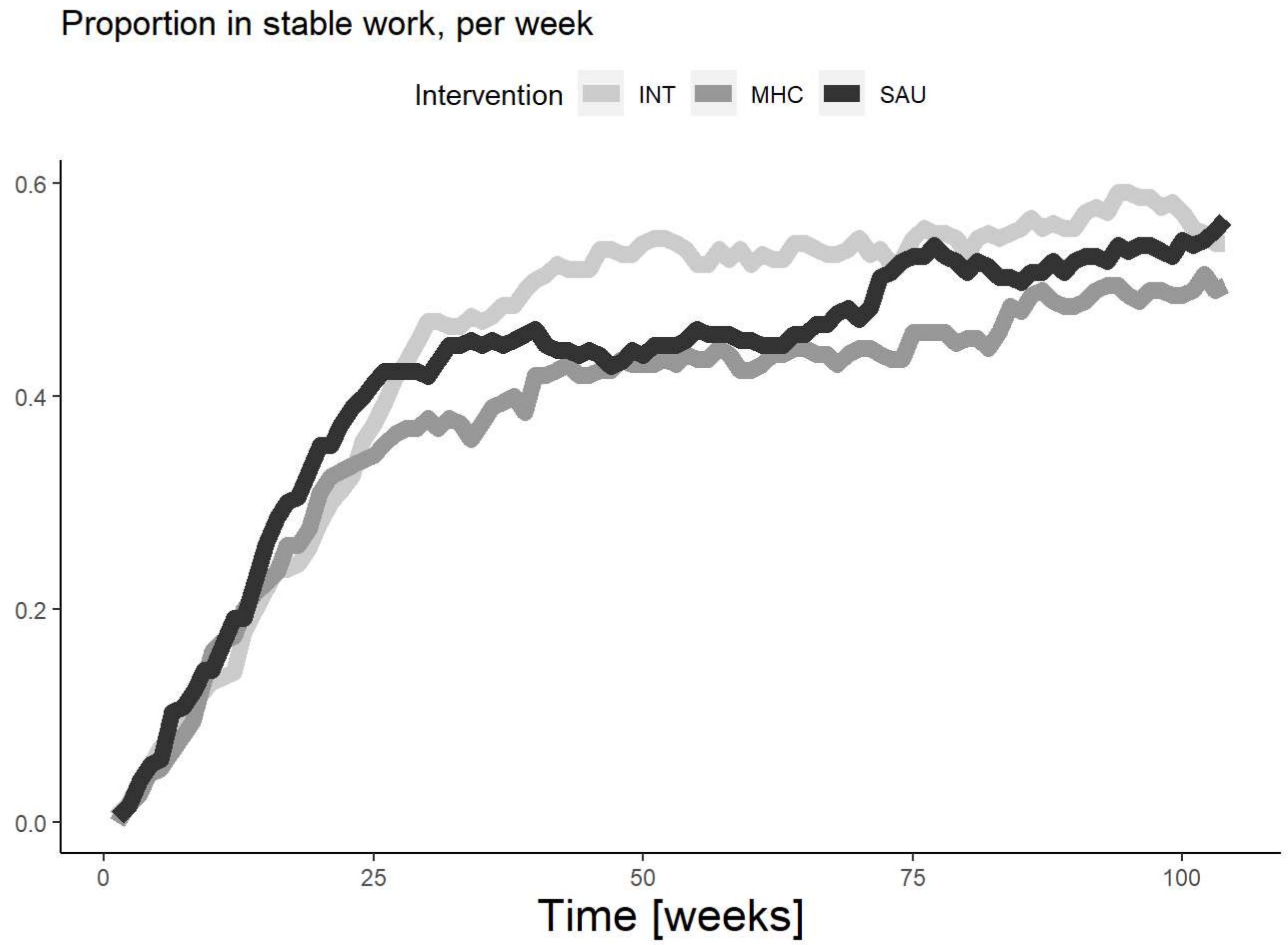

Vocational outcomes at 24-month follow-up from subgroup >interaction< in RCT1

	Group values			SAU-MHC					SAU-INT					MHC-INT				
	INT	MHC	SAU	Est.	p	low CI	:	high CI	Est.	p	low CI	:	high CI	Est.	p	low CI	:	high CI
RTW, FU24	30.0	37.0	31.0	1.70	**0.0081	1.05		2.74	1.06	0.77	0.68		1.64	0.61	**0.0122	0.38		0.98
RTW, FU24:stratdiagdepr				0.56	*0.019				0.81	0.38				1.49	0.1			
PROP, FU24	56.9	51.0	57.5	2.59	**0.0069	1.11		6.05	1.12	0.74	0.49		2.59	0.45	*0.0181	0.20		1.01
PROP, FU24:stratdiagdepr				-1.04	*0.0169				-0.11	0.80				0.90	*0.0361			
WEEKS, FU24	48.5	40.4	44.5	1.37	*0.023	0.98		1.92	0.97	0.76	0.74		1.26	0.70	**0.0091	0.51		0.97
WEEKS, FU24:stratdiagdepr				-0.40	0.05				-0.11	0.55				0.29	0.15			

Kaplan Meier-curve at 24-month follow-up regarding: interaction

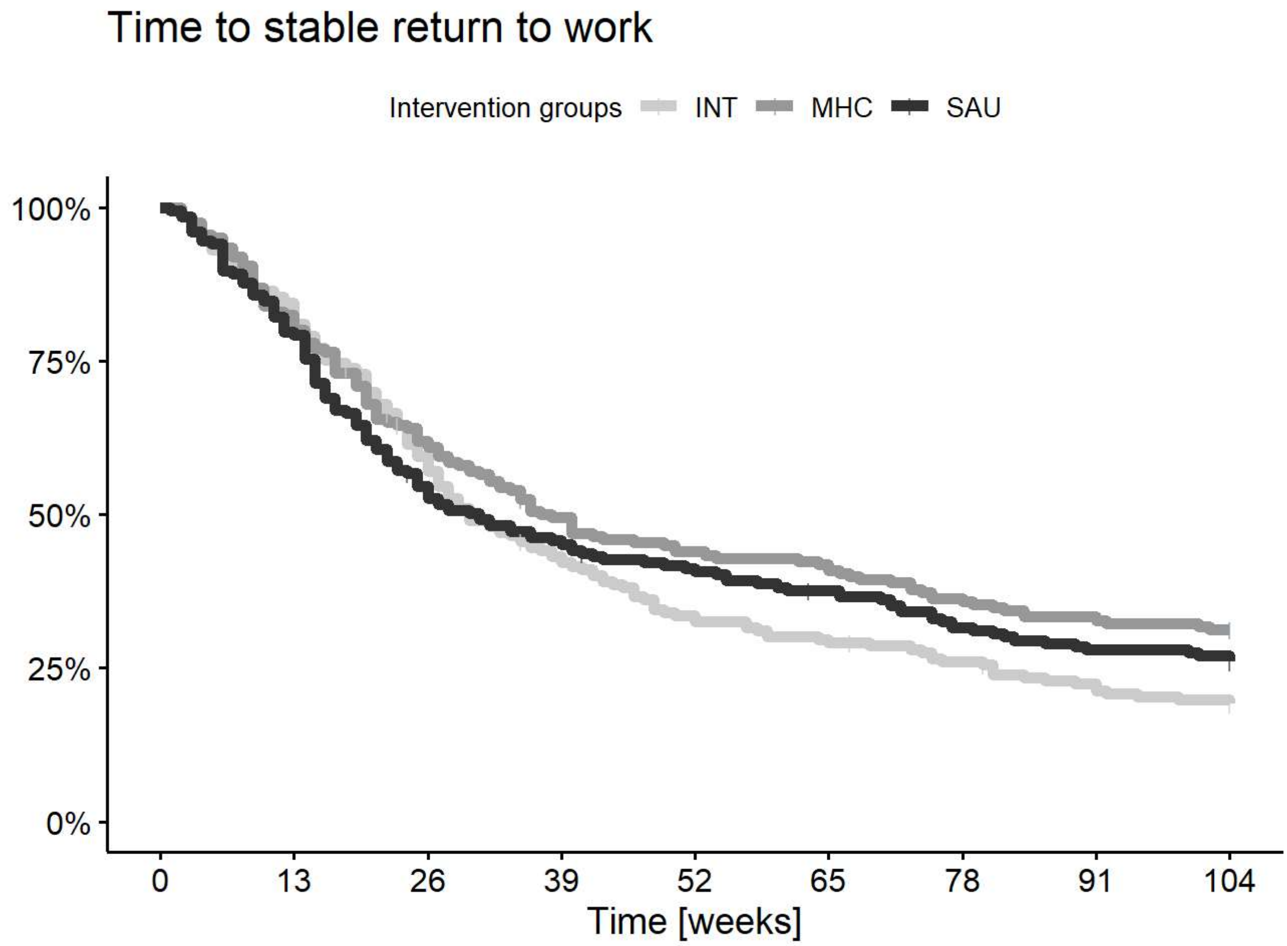

Self-reported outcomes at 24-month follow-up regarding: interaction

	Group values						SAU-MHC				SAU-INT				MHC-INT			
	INT	(SD)	MHC	(SD)	SAU	(SD)	Est.	p	low CI	high CI	Est.	p	low CI	high CI	Est.	p	low CI	high CI
BAI [FU24]	11.73	(9.04)	12.34	(9.32)	11.69	(9.17)	-0.71	0.62	-4.12	2.69	1.57	0.25	-1.69	4.83	2.31	0.08	-0.85	5.46
BDI [FU24]	11.21	(11.3)	11.41	(10.99)	10.9	(10.38)	-0.8	0.6	-4.45	2.86	0.33	0.83	-3.22	3.87	1.07	0.45	-2.35	4.5
PSS [FU24]	15.55	(8.37)	15.7	(7.2)	14.97	(7.59)	-0.38	0.71	-2.87	2.11	0.46	0.66	-2.05	2.98	0.83	0.41	-1.57	3.24
KES [FU24]	57.78	(19.77)	58.78	(19.58)	58.25	(18.42)	-1.54	0.54	-7.52	4.44	1.55	0.53	-4.42	7.52	3.08	0.21	-2.78	8.95
DSQ-som. [FU24]	9.39	(6.96)	9.43	(6.2)	9.73	(6.45)	0.37	0.71	-2.02	2.76	0.98	0.32	-1.38	3.34	0.64	0.51	-1.69	2.96
DSQ-distr. [FU24]	12.21	(8.33)	13.12	(8.25)	12.25	(8.51)	-1.06	0.34	-3.69	1.58	0.09	0.94	-2.56	2.74	1.14	0.29	-1.42	3.69
DSQ-anx. [FU24]	3.45	(4.36)	4.44	(5.2)	3.48	(4.54)	-1.26	0.11	-3.12	0.6	0.25	0.73	-1.51	2.01	1.51	*0.039	-0.24	3.26
DSQ-depr. [FU24]	1.62	(2.74)	1.81	(2.81)	1.72	(2.87)	-0.1	0.82	-1.13	0.94	-0.12	0.78	-1.15	0.91	-0.03	0.94	-1	0.94
WSAS [FU24]	11.5	(10.57)	12.01	(10.41)	11.2	(9.83)	-1.98	0.18	-5.54	1.58	0.07	0.96	-3.28	3.43	2.02	0.15	-1.32	5.36
SPS [FU24]	18.61	(4.29)	19.23	(3.78)	19.08	(3.39)	-0.25	0.84	-3.08	2.59	-0.16	0.88	-2.57	2.26	0.74	0.55	-2.18	3.65
IPQ [FU24]	17.49	(4.35)	16.77	(4.11)	16.94	(4.13)	0.02	0.98	-1.44	1.47	-0.8	0.17	-2.19	0.58	-0.81	0.16	-2.18	0.56
GSS [FU24]	28.13	(6.84)	28	(7.27)	29.15	(7.01)	0.51	0.62	-1.97	2.98	-0.22	0.82	-2.62	2.17	-0.76	0.44	-3.1	1.59
QoLs [FU24]	73.08	(16.69)	72.21	(17.2)	72.93	(15.46)	1.55	0.49	-3.81	6.9	-0.14	0.95	-5.3	5.02	-1.65	0.44	-6.78	3.47
EQ5 [FU24]	0.8	(0.18)	0.79	(0.16)	0.81	(0.15)	0.02	0.39	-0.03	0.07	-0.01	0.75	-0.06	0.05	-0.03	0.24	-0.08	0.03

Analyses regarding: >emp<

Proportion over time-curve at 24-month follow-up regarding: emp<

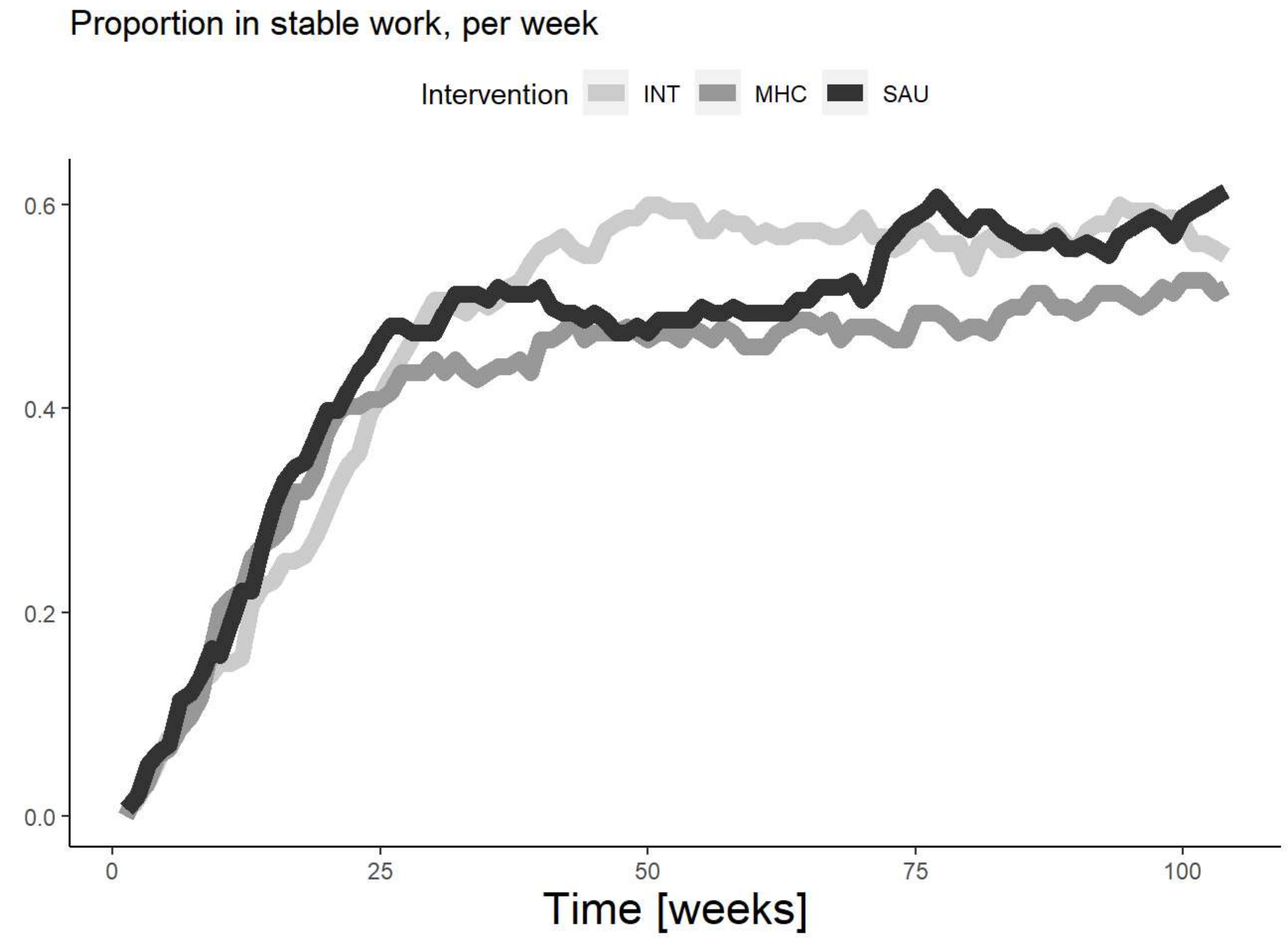

Vocational outcomes at 24-month follow-up from subgroup >emp< in RCT1

	Group values			SAU-MHC				SAU-INT				MHC-INT			
	INT	MHC	SAU	Est.	p	low CI	high CI	Est.	p	low CI	high CI	Est.	p	low CI	high CI
RTW, FU24	28	30	25	1.16	0.25	0.85	1.6	0.99	0.91	0.73	1.34	0.85	0.2	0.62	1.16
PROP, FU24	57.5	52.3	62.2	1.53	0.07	0.87	2.7	1.24	0.37	0.7	2.17	0.81	0.36	0.46	1.41
WEEKS, FU24	51.2	44.3	49	1.11	0.22	0.9	1.38	0.97	0.67	0.8	1.17	0.87	0.1	0.71	1.06

Kaplan Meier-curve at 24-month follow-up regarding: emp

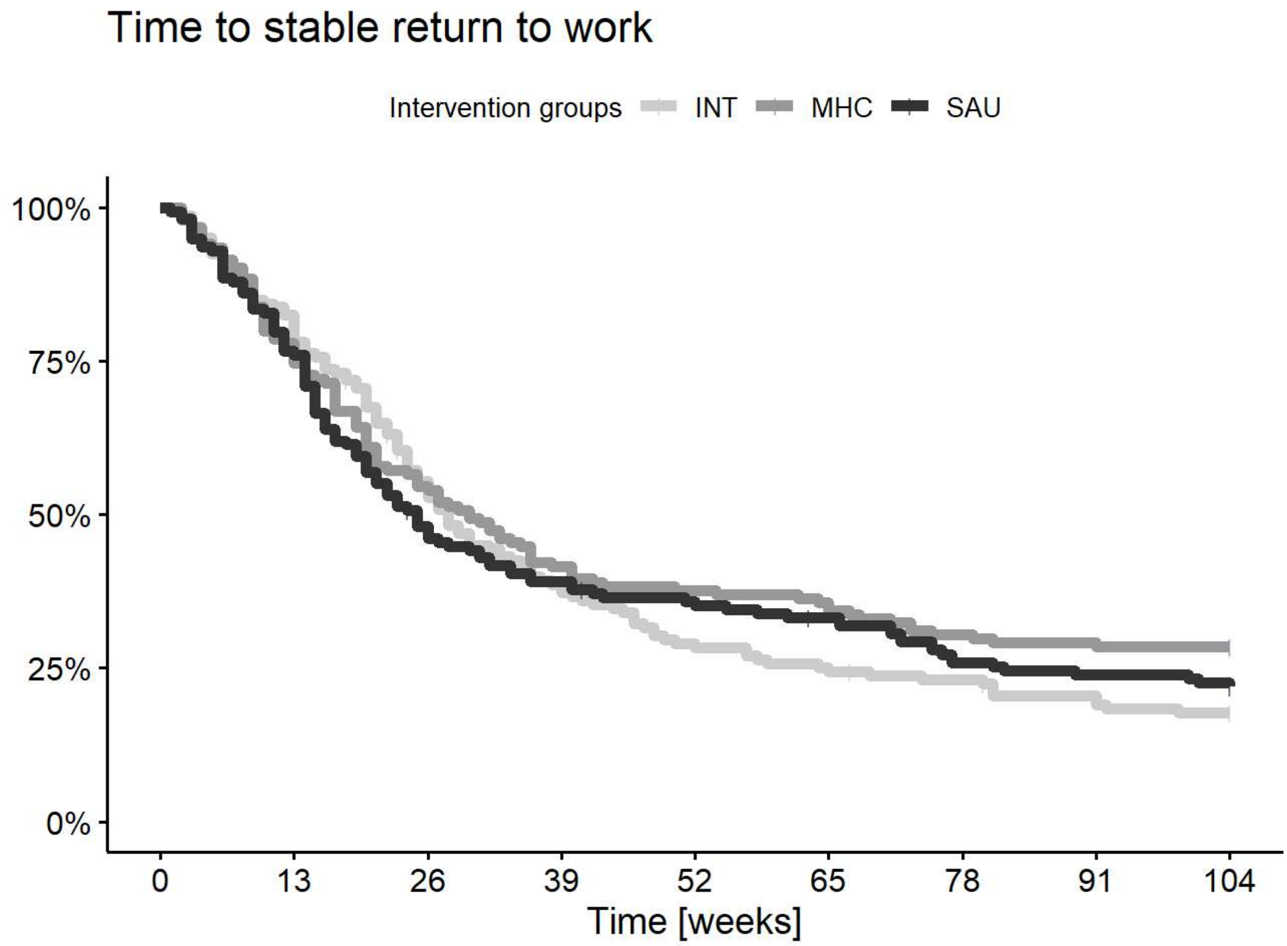

Self-reported outcomes at 24-month follow-up regarding: emp

	Group values						SAU-MHC				SAU-INT				MHC-INT			
	INT	(SD)	MHC	(SD)	SAU	(SD)	Est.	p	low CI	high CI	Est.	p	low CI	high CI	Est.	p	low CI	high CI
BAI [FU24]	12.07	(9.23)	12.91	(9.48)	12.19	(9.26)	0.13	0.91	-2.54	2.8	0.62	0.57	-2	3.23	0.53	0.62	-2.01	3.07
BDI [FU24]	11.5	(11.28)	11.98	(11.27)	10.94	(10.43)	-0.47	0.71	-3.49	2.54	-0.16	0.9	-3.12	2.8	0.32	0.79	-2.53	3.18
PSS [FU24]	15.47	(8.43)	16.29	(7.24)	15.03	(7.78)	-0.52	0.55	-2.61	1.57	0.13	0.89	-2.01	2.27	0.66	0.43	-1.36	2.67
KES [FU24]	58.11	(19.62)	59.47	(19.68)	59.01	(18.51)	-0.06	0.98	-4.84	4.72	0.83	0.68	-3.98	5.64	0.92	0.65	-3.88	5.72
DSQ-som. [FU24]	9.53	(6.81)	9.7	(6.19)	9.98	(6.38)	0.3	0.7	-1.57	2.17	0.36	0.64	-1.47	2.19	0.08	0.92	-1.77	1.92
DSQ-distr. [FU24]	12.33	(8.33)	13.61	(8.26)	12.38	(8.5)	-0.87	0.34	-3.05	1.31	0.1	0.91	-2.07	2.28	0.98	0.26	-1.11	3.06
DSQ-anx. [FU24]	3.58	(4.49)	4.56	(5.31)	3.66	(4.72)	-0.48	0.42	-1.9	0.94	0.04	0.94	-1.32	1.4	0.54	0.35	-0.84	1.92
DSQ-depr. [FU24]	1.59	(2.65)	1.83	(2.74)	1.89	(3.08)	-0.01	0.98	-0.84	0.82	0.14	0.67	-0.67	0.96	0.15	0.64	-0.62	0.92
WSAS [FU24]	11.81	(10.71)	12.52	(10.72)	10.97	(9.96)	-1.04	0.39	-3.97	1.88	-0.39	0.73	-3.14	2.35	0.66	0.57	-2.12	3.44
SPS [FU24]	18.69	(4.18)	19.35	(3.69)	19.59	(2.96)	0.73	0.36	-1.15	2.61	0.98	0.16	-0.66	2.61	0.31	0.68	-1.46	2.07
IPQ [FU24]	17.42	(4.45)	16.59	(4.13)	16.88	(4.27)	0.27	0.57	-0.89	1.44	-0.22	0.65	-1.37	0.93	-0.49	0.3	-1.63	0.64
GSS [FU24]	27.95	(6.96)	27.7	(7.31)	29.24	(7.23)	0.76	0.37	-1.27	2.79	0.72	0.37	-1.23	2.67	-0.05	0.95	-1.97	1.87
QoLs [FU24]	73.04	(16.64)	72.03	(17.55)	72.83	(15.61)	0.36	0.84	-3.94	4.66	0.18	0.92	-3.94	4.3	-0.2	0.91	-4.38	3.97
EQ5 [FU24]	0.79	(0.18)	0.78	(0.17)	0.81	(0.15)	0.02	0.41	-0.03	0.06	0.01	0.52	-0.03	0.06	0	0.85	-0.05	0.04

Analyses regarding: >vac<

Proportion over time-curve at 24-month follow-up regarding: vac<

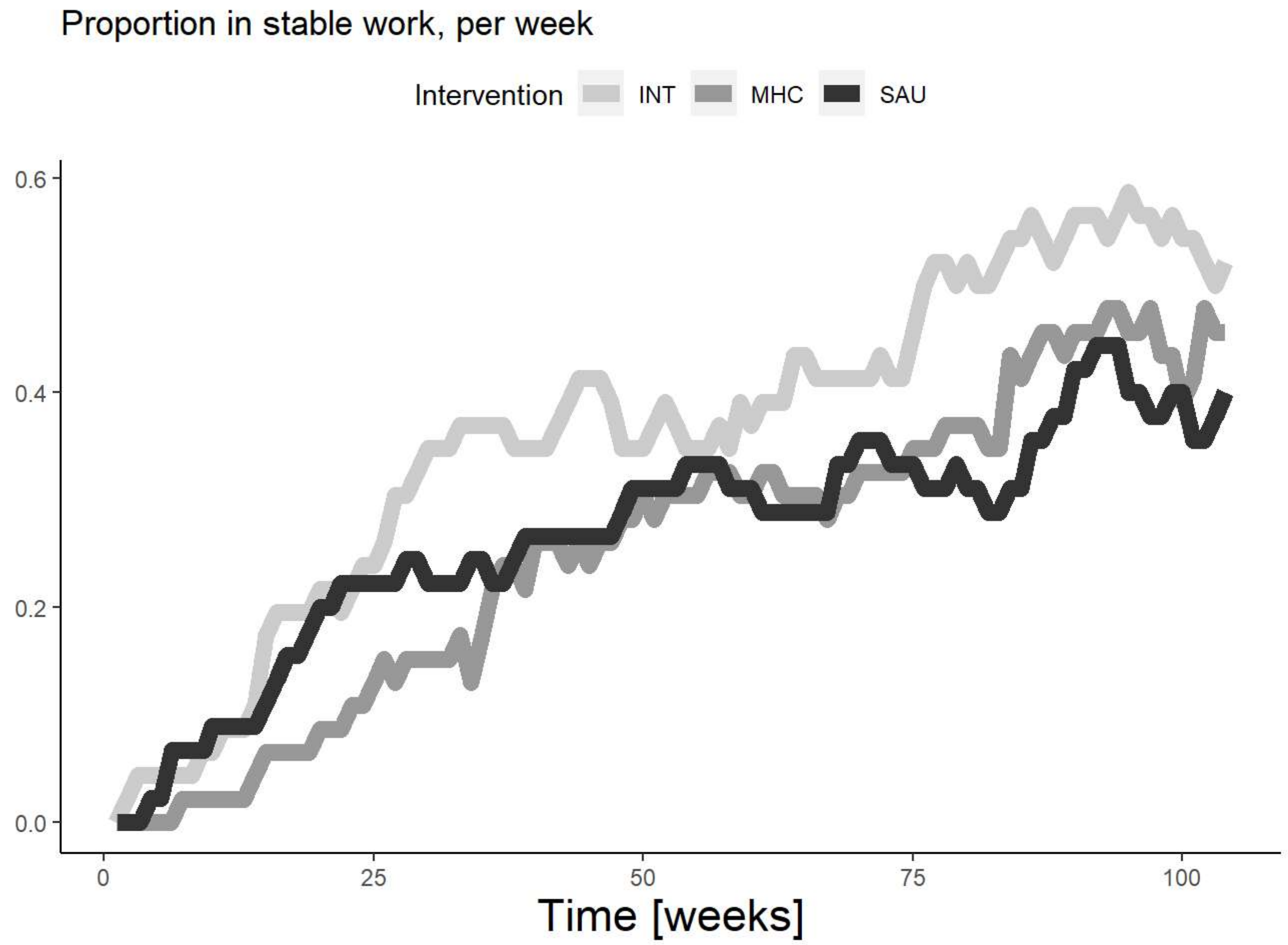

Vocational outcomes at 24-month follow-up from subgroup >vac< in RCT1

	Group values			SAU-MHC				SAU-INT				MHC-INT			
	**INT**	**MHC**	**SAU**	**Est.**	**p**	**low CI**	**high CI**	**Est.**	**p**	**low CI**	**high CI**	**Est.**	**p**	**low CI**	**high CI**
RTW, FU24	48	84	79	1.12	0.68	0.56	2.25	0.73	0.24	0.39	1.39	0.67	0.12	0.35	1.26
PROP, FU24	54.5	46.7	40.9	0.78	0.57	0.26	2.3	0.6	0.25	0.2	1.77	0.71	0.45	0.23	2.17
WEEKS, FU24	39.4	27.3	28.6	1.05	0.85	0.59	1.87	0.75	0.17	0.45	1.25	0.7	0.08	0.43	1.14

Kaplan Meier-curve at 24-month follow-up regarding: vac

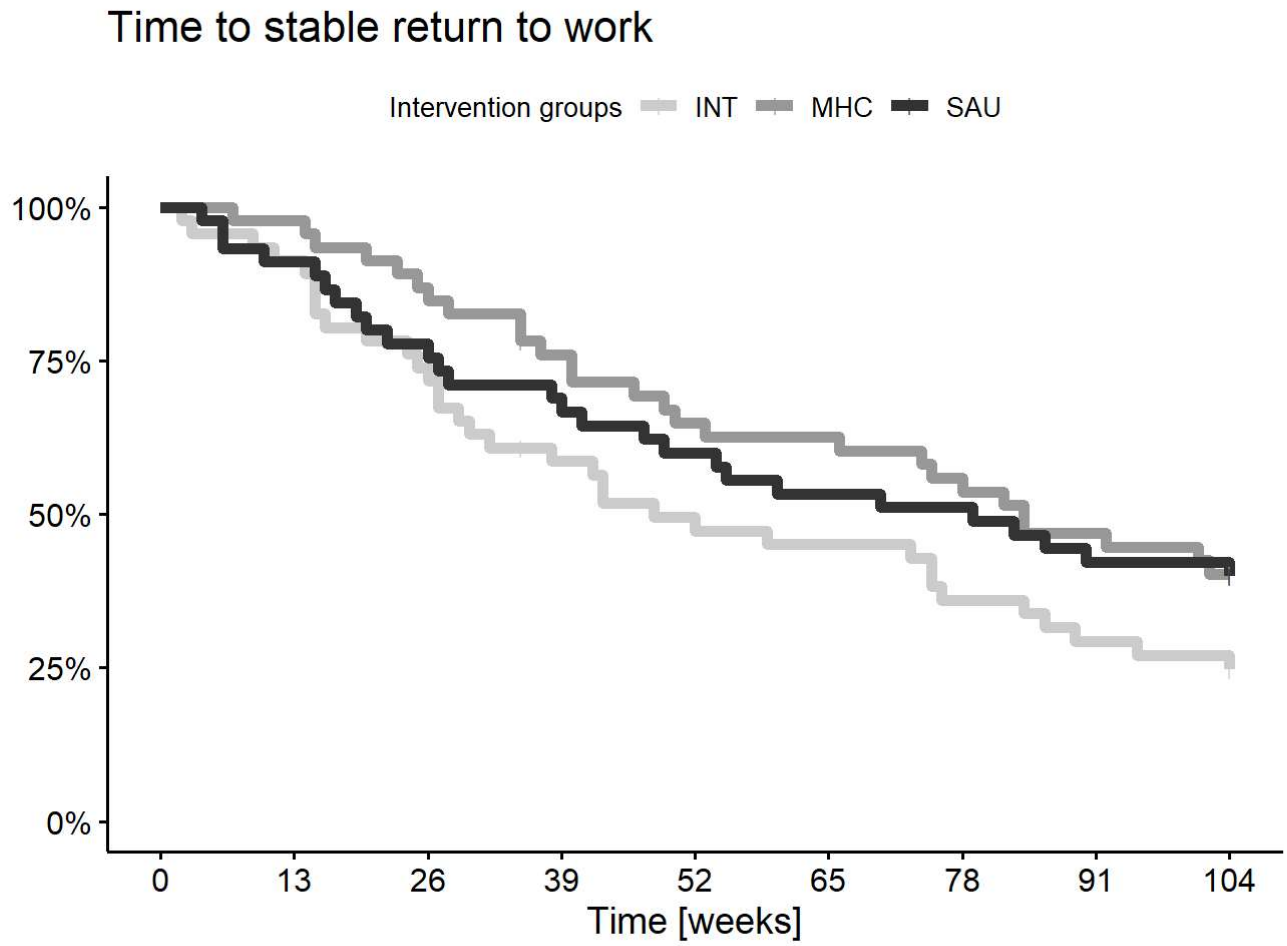

Self-reported outcomes at 24-month follow-up regarding: vac

	Group values						SAU-MHC				SAU-INT				MHC-INT			
	INT	(SD)	MHC	(SD)	SAU	(SD)	Est.	p	low CI	high CI	Est.	p	low CI	high CI	Est.	p	low CI	high CI
BAI [FU24]	10.04	(7.93)	9.8	(8.23)	9.55	(8.63)	0.79	0.72	-4.5	6.09	1.35	0.52	-3.68	6.39	0.59	0.75	-3.96	5.14
BDI [FU24]	9.73	(11.53)	8.87	(9.41)	10.72	(10.39)	1.41	0.56	-4.44	7.26	1.79	0.45	-3.92	7.5	0.45	0.84	-4.91	5.82
PSS [FU24]	15.96	(8.25)	13.08	(6.56)	14.68	(6.85)	1.22	0.48	-2.95	5.39	-0.13	0.94	-4.21	3.96	-1.33	0.42	-5.33	2.67
KES [FU24]	56	(20.96)	55.72	(19.21)	54.86	(18.04)	-0.11	0.98	-9.5	9.28	-1	0.8	-10.34	8.35	-0.73	0.84	-9.58	8.12
DSQ-som. [FU24]	8.65	(7.83)	8.24	(6.23)	8.68	(6.8)	0.39	0.8	-3.27	4.05	0.83	0.59	-2.85	4.51	0.46	0.75	-2.99	3.9
DSQ-distr. [FU24]	11.57	(8.48)	10.96	(8.01)	11.68	(8.73)	0.29	0.87	-3.96	4.54	0.01	0.99	-4.31	4.34	-0.21	0.9	-4.28	3.85
DSQ-anx. [FU24]	2.74	(3.58)	3.92	(4.72)	2.73	(3.69)	0.19	0.87	-2.66	3.04	0.9	0.43	-1.87	3.67	0.7	0.51	-1.85	3.25
DSQ-depr. [FU24]	1.74	(3.24)	1.72	(3.16)	1	(1.6)	-0.03	0.97	-1.74	1.68	-0.05	0.94	-1.76	1.66	0	1	-1.61	1.61
WSAS [FU24]	9.92	(9.91)	9.76	(8.73)	12.18	(9.43)	1.47	0.54	-4.3	7.23	2.03	0.38	-3.58	7.65	0.66	0.77	-4.83	6.15
SPS [FU24]	18.25	(4.82)	18.62	(4.29)	16.36	(4.29)	0.73	0.36	-1.15	2.61	0.98	0.16	-0.66	2.61	0.31	0.68	-1.46	2.07
IPQ [FU24]	17.9	(3.79)	17.52	(3.99)	17.19	(3.52)	-0.17	0.87	-2.56	2.22	-1.1	0.26	-3.45	1.26	-0.88	0.35	-3.15	1.39
GSS [FU24]	29.19	(6.21)	29.28	(7.09)	28.71	(6.03)	-0.37	0.82	-4.2	3.46	-1.13	0.45	-4.73	2.48	-0.7	0.64	-4.35	2.95
QoLs [FU24]	73.29	(17.41)	73	(15.91)	73.38	(15.14)	0.28	0.94	-7.99	8.55	0.52	0.88	-7.61	8.66	0.2	0.95	-7.43	7.83
EQ5 [FU24]	0.83	(0.14)	0.84	(0.12)	0.81	(0.13)	-0.02	0.59	-0.1	0.06	-0.02	0.65	-0.1	0.07	0	0.93	-0.07	0.08

Analyses regarding: >intempl<

Proportion over time-curve at 24-month follow-up regarding: intempl<

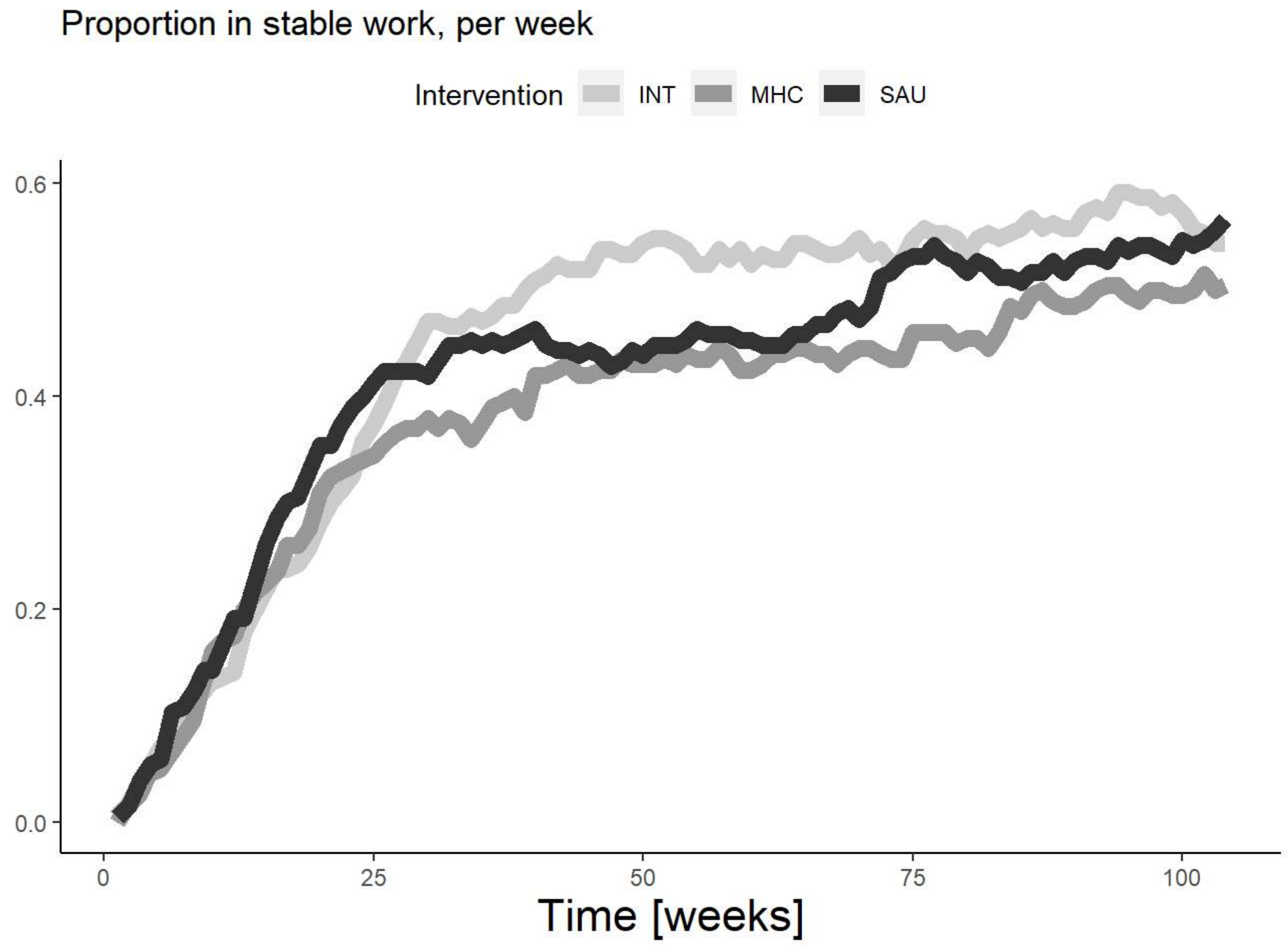

Vocational outcomes at 24-month follow-up from subgroup >intempl< in RCT1

	Group values			SAU-MHC					SAU-INT					MHC-INT				
	**INT**	**MHC**	**SAU**	**Est.**	**p**	**low CI**	**:**	**high CI**	**Est.**	**p**	**low CI**	**:**	**high CI**	**Est.**	**p**	**low CI**	**:**	**high CI**
RTW, FU24	30	37	31	1.17	0.24	0.85		1.61	0.98	0.87	0.72		1.33	0.83	0.15	0.61		1.13
RTW, FU24:stratlabormarketvac				0.99	0.97				0.76	0.35				0.8	0.44			
PROP, FU24	56.9	51	57.5	1.54	0.07	0.87		2.7	1.24	0.36	0.7		2.17	0.8	0.35	0.46		1.41
PROP, FU24:stratlabormarketvac				-0.68	0.17				-0.74	0.13				-0.11	0.83			
WEEKS, FU24	48.5	40.4	44.5	1.11	0.22	0.9		1.38	0.97	0.69	0.8		1.17	0.87	0.09	0.71		1.06
WEEKS, FU24:stratlabormarketvac				0.22	0.52				-0.26	0.38				-0.47	0.14			

Kaplan Meier-curve at 24-month follow-up regarding: intempl

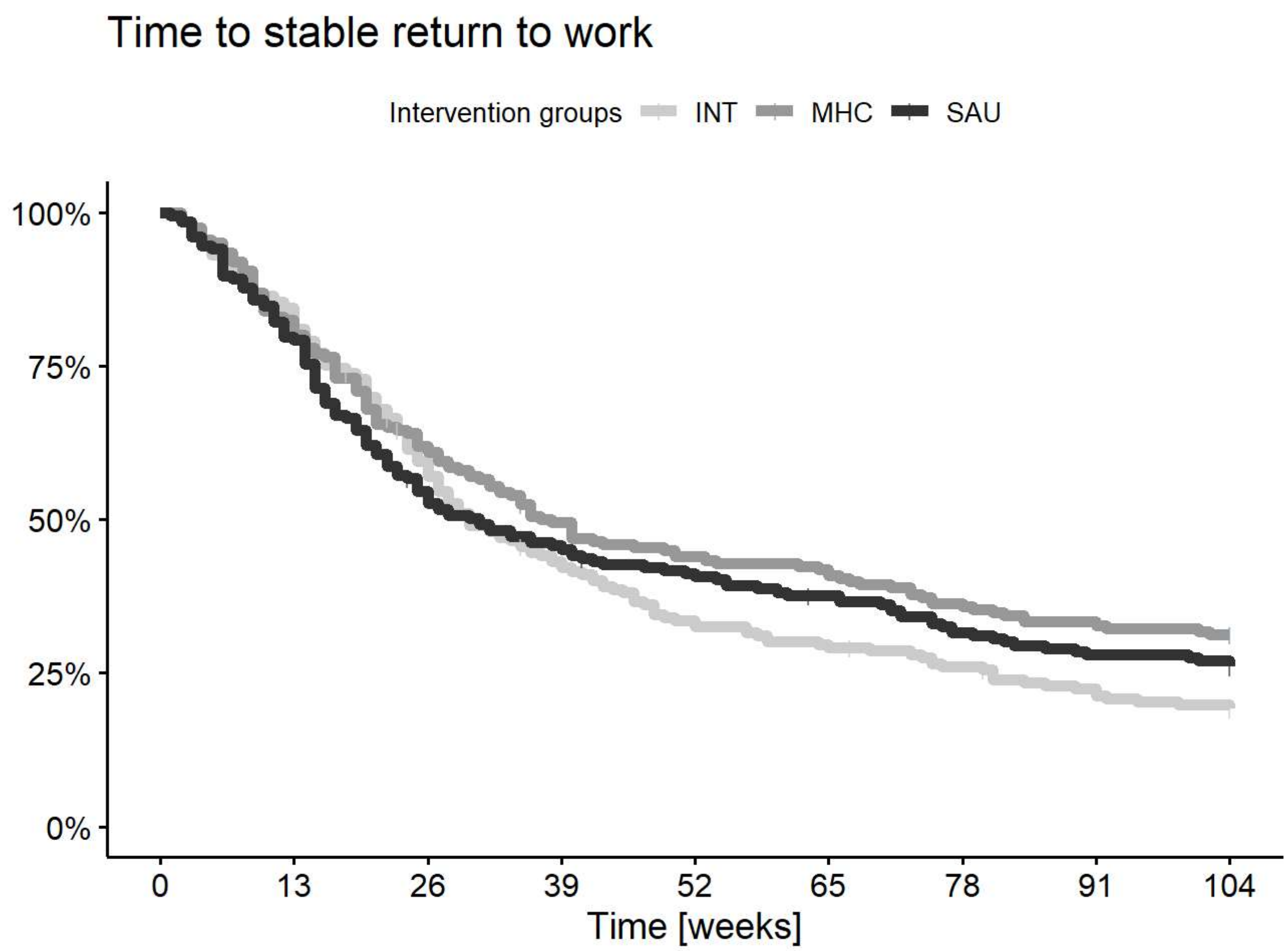

Analyses regarding: >north<

Proportion over time-curve at 24-month follow-up regarding: north<

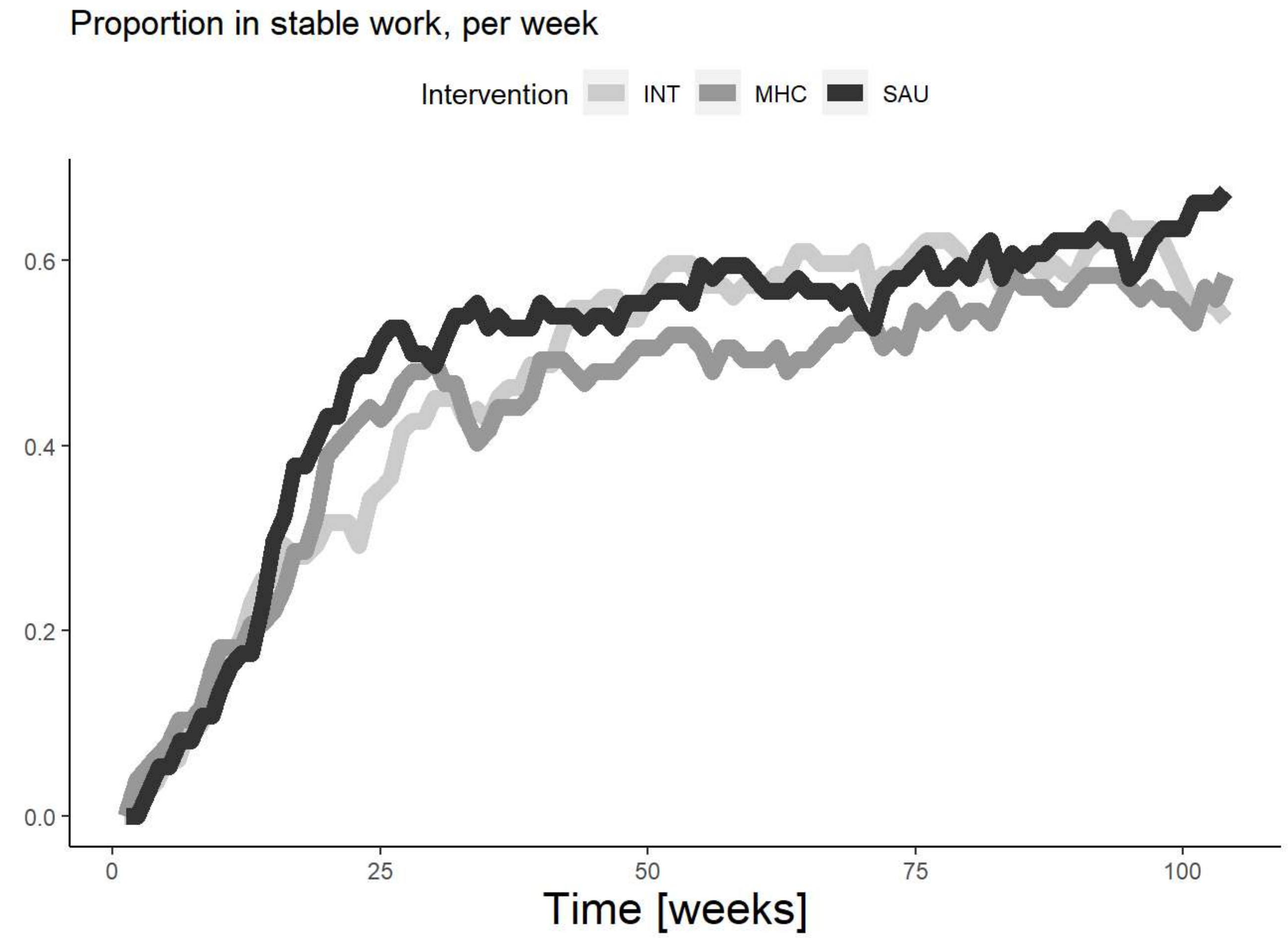

Vocational outcomes at 24-month follow-up from subgroup >north< in RCT1

	Group values			SAU-MHC				SAU-INT				MHC-INT			
	INT	MHC	SAU	Est.	p	low CI	high CI	Est.	p	low CI	high CI	Est.	p	low CI	high CI
RTW, FU24	30	27	23	1.12	0.53	0.71	1.77	1.11	0.56	0.72	1.72	0.97	0.87	0.63	1.51
PROP, FU24	56.4	58.4	67.6	1.48	0.25	0.65	3.41	1.6	0.17	0.7	3.63	1.06	0.86	0.48	2.36
WEEKS, FU24	51.3	46.8	51.9	1.11	0.38	0.84	1.47	1.02	0.89	0.78	1.31	0.91	0.42	0.68	1.21

Kaplan Meier-curve at 24-month follow-up regarding: north

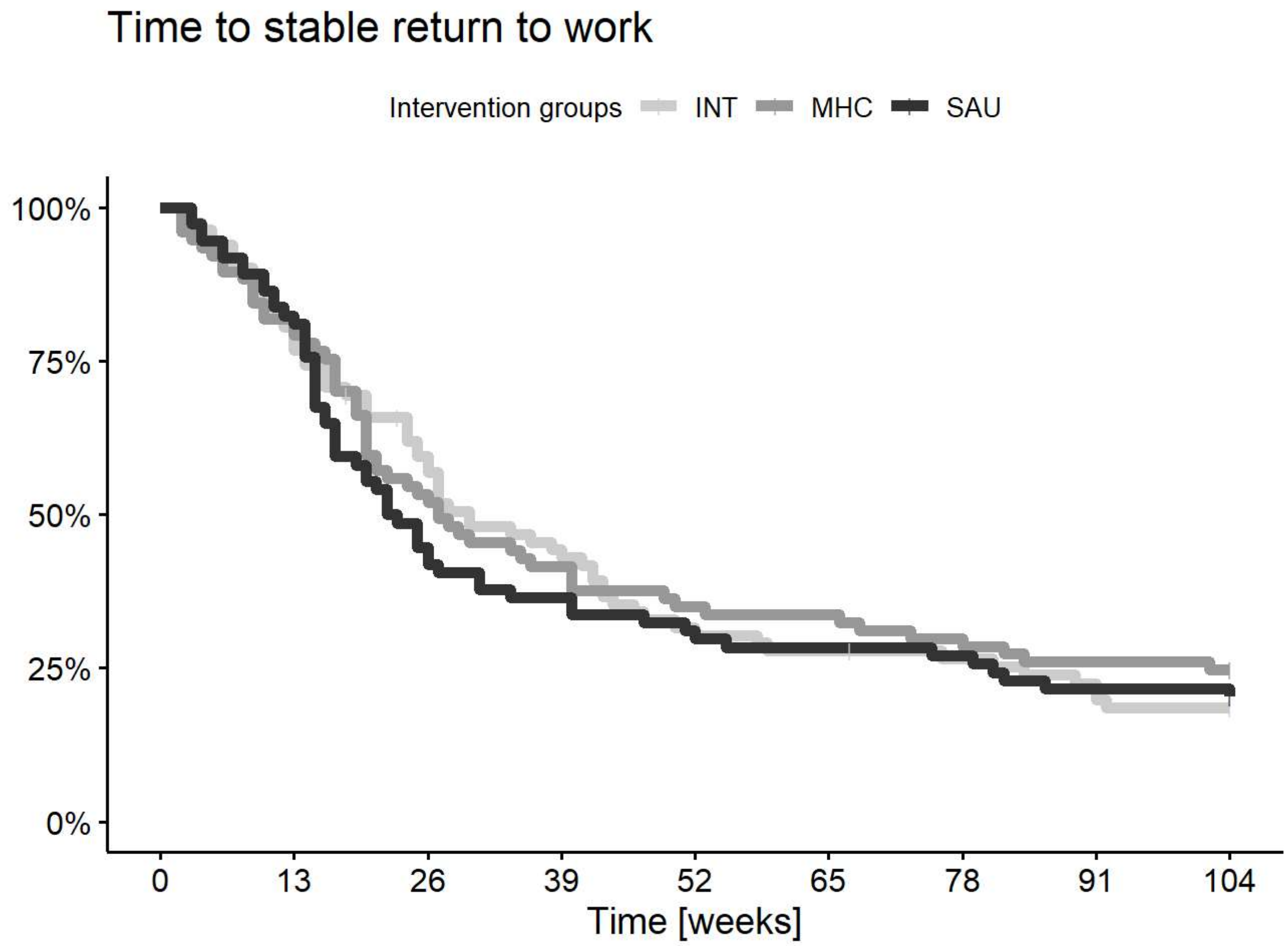

Self-reported outcomes at 24-month follow-up regarding: north

	Group values						SAU-MHC				SAU-INT				MHC-INT			
	INT	(SD)	MHC	(SD)	SAU	(SD)	Est.	p	low CI	high CI	Est.	p	low CI	high CI	Est.	p	low CI	high CI
BAI [FU24]	11.76	(8.8)	11.79	(8.33)	11.72	(10.1)	0.09	0.95	-3.51	3.7	-0.01	0.99	-3.66	3.64	-0.1	0.95	-3.56	3.37
BDI [FU24]	11.39	(11.33)	10.24	(9.77)	11.75	(10.59)	1.28	0.45	-2.82	5.37	0.8	0.63	-3.23	4.82	-0.48	0.77	-4.38	3.42
PSS [FU24]	16.44	(8.01)	14.58	(6.7)	14.52	(7.92)	0.13	0.92	-2.74	2.99	-1.24	0.31	-4.19	1.71	-1.36	0.24	-4.13	1.41
KES [FU24]	60.63	(19.79)	56.73	(17.55)	57.68	(18.3)	1.24	0.66	-5.56	8.04	-1.47	0.61	-8.29	5.36	-2.69	0.33	-9.27	3.89
DSQ-som. [FU24]	9.96	(7.45)	9.58	(6.05)	9.56	(5.83)	-0.09	0.93	-2.65	2.46	-0.47	0.67	-3.08	2.15	-0.39	0.72	-3.01	2.23
DSQ-distr. [FU24]	12.8	(8.17)	12.06	(7.45)	12.52	(8.85)	0.31	0.81	-2.75	3.36	-0.01	0.99	-3.18	3.16	-0.32	0.79	-3.26	2.63
DSQ-anx. [FU24]	2.93	(3.32)	3.75	(4.93)	3.16	(3.92)	-0.13	0.87	-2	1.74	-0.03	0.97	-1.89	1.83	0.15	0.85	-1.74	2.04
DSQ-depr. [FU24]	1.57	(2.28)	1.47	(2.41)	1.72	(2.87)	0.29	0.53	-0.81	1.39	0.15	0.75	-0.95	1.25	-0.14	0.75	-1.2	0.91
WSAS [FU24]	12.89	(11.59)	11.68	(10.14)	11.24	(10.05)	-0.44	0.8	-4.65	3.77	-1.03	0.54	-5.1	3.04	-0.56	0.74	-4.57	3.45
SPS [FU24]	18.63	(4.04)	18.43	(4.01)	18.51	(4.03)	0.65	0.39	-1.15	2.45	0.81	0.25	-0.87	2.49	0.42	0.57	-1.34	2.18
IPQ [FU24]	16.49	(4.68)	17.1	(3.61)	16.76	(3.86)	-0.35	0.62	-2.04	1.34	0.17	0.81	-1.51	1.84	0.52	0.45	-1.13	2.16
GSS [FU24]	28.09	(6.21)	28	(6.92)	28.6	(7.15)	0.27	0.82	-2.57	3.11	0.44	0.7	-2.26	3.14	0.16	0.89	-2.54	2.86
QoLs [FU24]	73.19	(16.47)	75.1	(15.63)	72.28	(15.89)	-2.48	0.33	-8.54	3.59	-0.78	0.76	-6.82	5.26	1.65	0.49	-4.08	7.38
EQ5 [FU24]	0.78	(0.2)	0.8	(0.13)	0.8	(0.14)	0	0.88	-0.06	0.07	0.02	0.39	-0.04	0.09	0.02	0.5	-0.05	0.08

Analyses regarding: >city<

Proportion over time-curve at 24-month follow-up regarding: city<

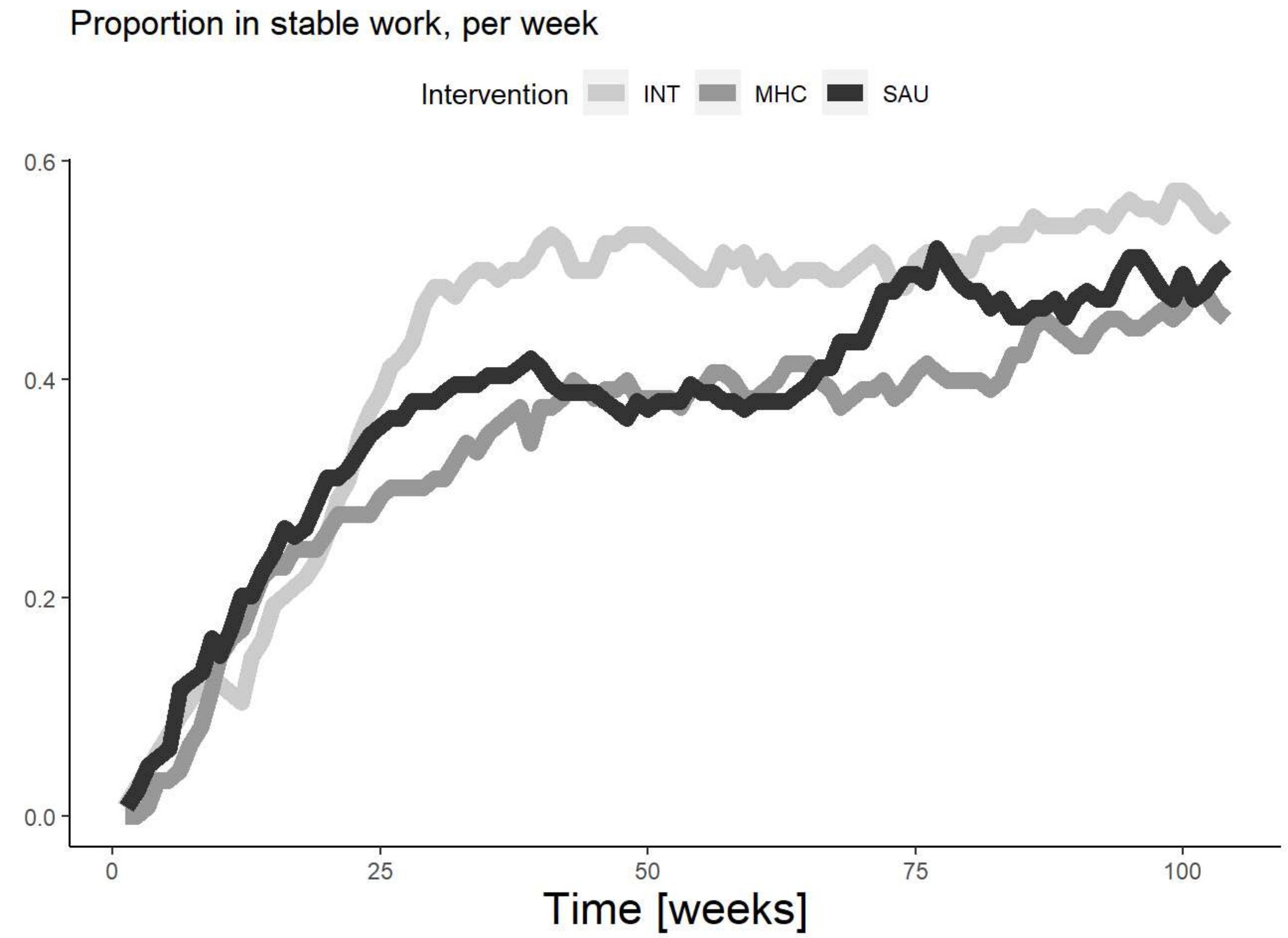

Vocational outcomes at 24-month follow-up from subgroup >city< in RCT1

	Group values			SAU-MHC				SAU-INT				MHC-INT			
	**INT**	**MHC**	**SAU**	**Est.**	**p**	**low CI**	**:high CI**	**Est.**	**p**	**low CI**	**:high CI**	**Est.**	**p**	**low CI**	**:high CI**
RTW, FU24	30.0	50.0	41.0	1.20	0.23	0.83	1.74	0.82	0.19	0.58	1.17	0.68	**0.0107	0.47	0.98
PROP, FU24	57.1	46.3	51.6	1.24	0.41	0.66	2.30	0.79	0.38	0.42	1.50	0.65	0.1	0.34	1.22
WEEKS, FU24	46.7	36.4	40.2	1.10	0.41	0.83	1.46	0.87	0.15	0.68	1.10	0.79	*0.0248	0.61	1.02

Kaplan Meier-curve at 24-month follow-up regarding: city

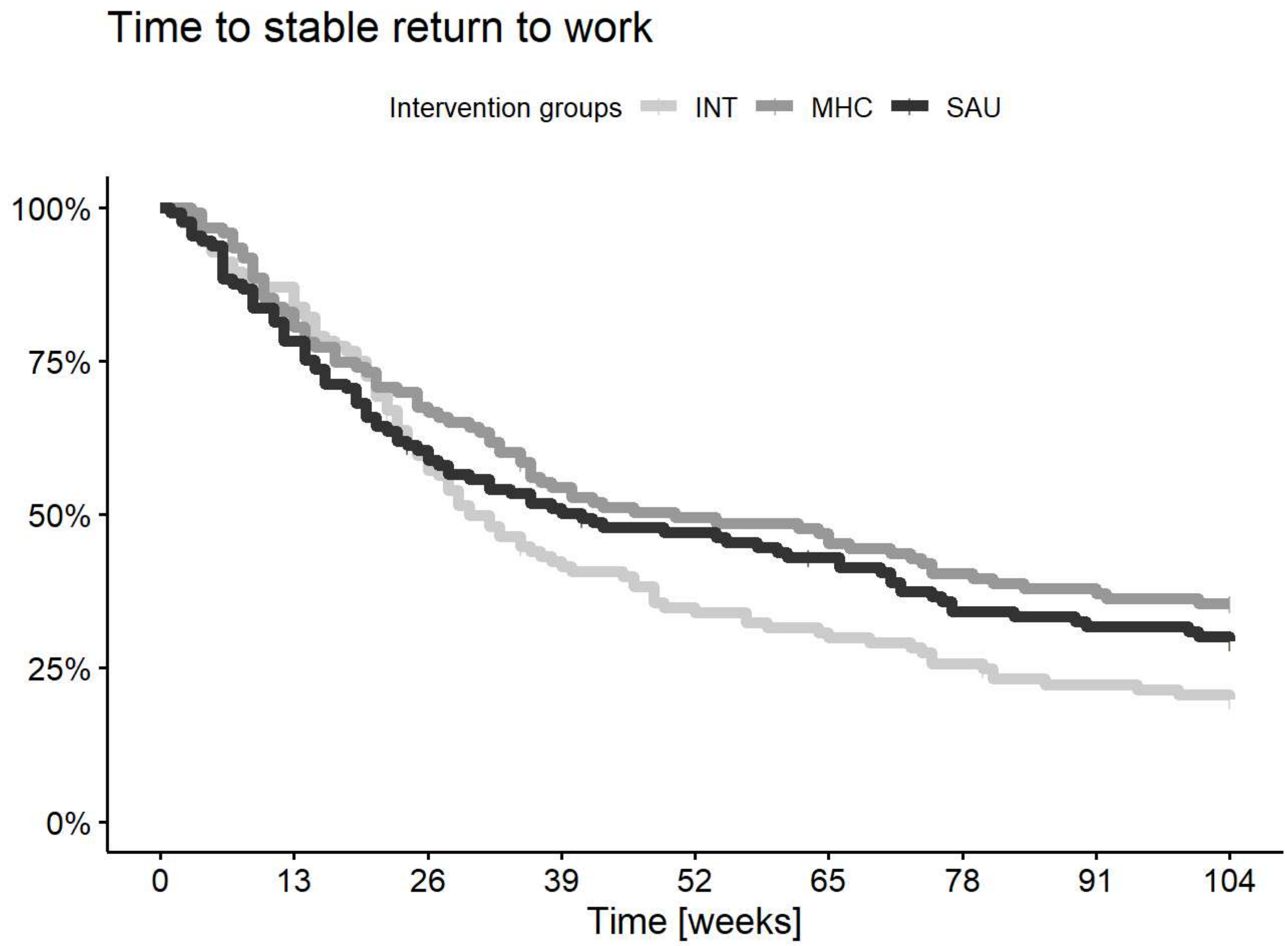

Self-reported outcomes at 24-month follow-up regarding: city

	Group values						SAU-MHC				SAU-INT				MHC-INT			
	INT	(SD)	MHC	(SD)	SAU	(SD)	Est.	p	low CI	high CI	Est.	p	low CI	high CI	Est.	p	low CI	high CI
BAI [FU24]	11.71	(9.22)	12.69	(9.92)	11.67	(8.49)	0.38	0.77	-2.73	3.49	1.25	0.32	-1.75	4.26	0.88	0.47	-2.02	3.79
BDI [FU24]	11.1	(11.34)	12.15	(11.69)	10.25	(10.25)	-0.85	0.56	-4.32	2.63	-0.03	0.99	-3.5	3.45	0.83	0.55	-2.52	4.17
PSS [FU24]	15.02	(8.58)	16.4	(7.45)	15.3	(7.37)	-0.28	0.78	-2.7	2.14	0.89	0.4	-1.66	3.44	1.17	0.23	-1.19	3.53
KES [FU24]	56.03	(19.67)	60.02	(20.72)	58.69	(18.64)	-0.86	0.71	-6.39	4.68	1.67	0.47	-3.92	7.27	2.55	0.27	-2.94	8.04
DSQ-som. [FU24]	9.04	(6.66)	9.35	(6.33)	9.86	(6.93)	0.57	0.53	-1.59	2.73	1.05	0.24	-1.09	3.2	0.49	0.58	-1.61	2.59
DSQ-distr. [FU24]	11.85	(8.45)	13.76	(8.68)	12.05	(8.31)	-1.14	0.27	-3.62	1.34	0.17	0.87	-2.32	2.65	1.31	0.19	-1.11	3.74
DSQ-anx. [FU24]	3.76	(4.87)	4.87	(5.33)	3.73	(4.98)	-0.45	0.52	-2.17	1.26	0.36	0.6	-1.28	2	0.82	0.22	-0.77	2.41
DSQ-depr. [FU24]	1.64	(3)	2.01	(3.02)	1.73	(2.9)	-0.19	0.65	-1.19	0.81	0.09	0.83	-0.89	1.07	0.28	0.47	-0.65	1.2
WSAS [FU24]	10.67	(9.9)	12.23	(10.63)	11.16	(9.74)	-0.52	0.71	-3.86	2.82	0.89	0.5	-2.31	4.08	1.42	0.29	-1.8	4.65
SPS [FU24]	18.6	(4.46)	19.68	(3.6)	19.53	(2.74)	0.65	0.39	-1.15	2.45	0.81	0.25	-0.87	2.49	0.42	0.57	-1.34	2.18
IPQ [FU24]	18.09	(4.04)	16.57	(4.39)	17.08	(4.34)	0.51	0.37	-0.86	1.87	-0.78	0.16	-2.1	0.54	-1.29	*0.0179	-2.59	0.01
GSS [FU24]	28.16	(7.23)	28	(7.51)	29.57	(6.91)	0.66	0.49	-1.65	2.96	0.26	0.79	-2.03	2.55	-0.41	0.66	-2.65	1.84
QoLs [FU24]	73.01	(16.92)	70.47	(17.95)	73.43	(15.22)	2.04	0.32	-2.85	6.94	0.81	0.68	-3.95	5.58	-1.25	0.53	-6.05	3.56
EQ5 [FU24]	0.81	(0.16)	0.79	(0.18)	0.81	(0.15)	0.01	0.65	-0.04	0.06	0	0.83	-0.05	0.05	-0.01	0.49	-0.06	0.04

Analyses regarding: >intteams<

Proportion over time-curve at 24-month follow-up regarding: intteams<

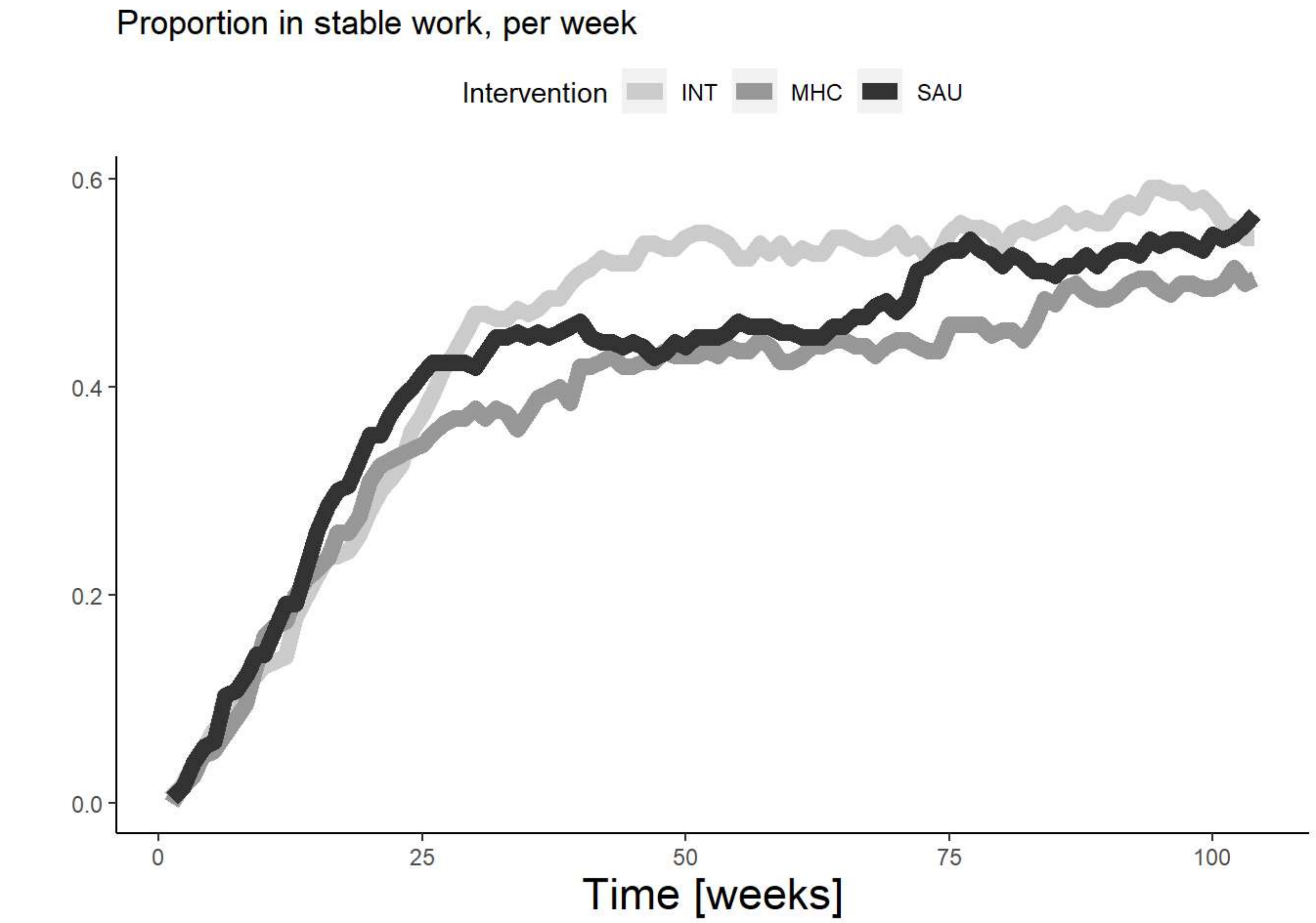

Vocational outcomes at 24-month follow-up from subgroup >intteams< in RCT1

	Group values			SAU-MHC				SAU-INT				MHC-INT			
	**INT**	**MHC**	**SAU**	**Est.**	**p**	**low CI**	**high CI**	**Est.**	**p**	**low CI**	**high CI**	**Est.**	**p**	**low CI**	**high CI**
RTW, FU24	30.0	37.0	31.0	1.20	0.23	0.83	1.74	0.83	0.20	0.58	1.18	0.69	**0.0134	0.48	0.99
RTW, FU24:teamnorth				0.93	0.76			1.34	0.21			1.41	0.14		
PROP, FU24	56.9	51.0	57.5	1.23	0.41	0.67	2.29	0.80	0.39	0.43	1.49	0.65	0.1	0.35	1.21
PROP, FU24:teamnorth				0.19	0.65			0.73	0.09			0.51	0.23		
WEEKS, FU24	48.5	40.4	44.5	1.10	0.41	0.83	1.45	0.87	0.15	0.68	1.10	0.79	*0.0248	0.61	1.02
WEEKS, FU24:teamnorth				-0.01	0.98			0.23	0.20			0.23	0.23		

Kaplan Meier-curve at 24-month follow-up regarding: intteams

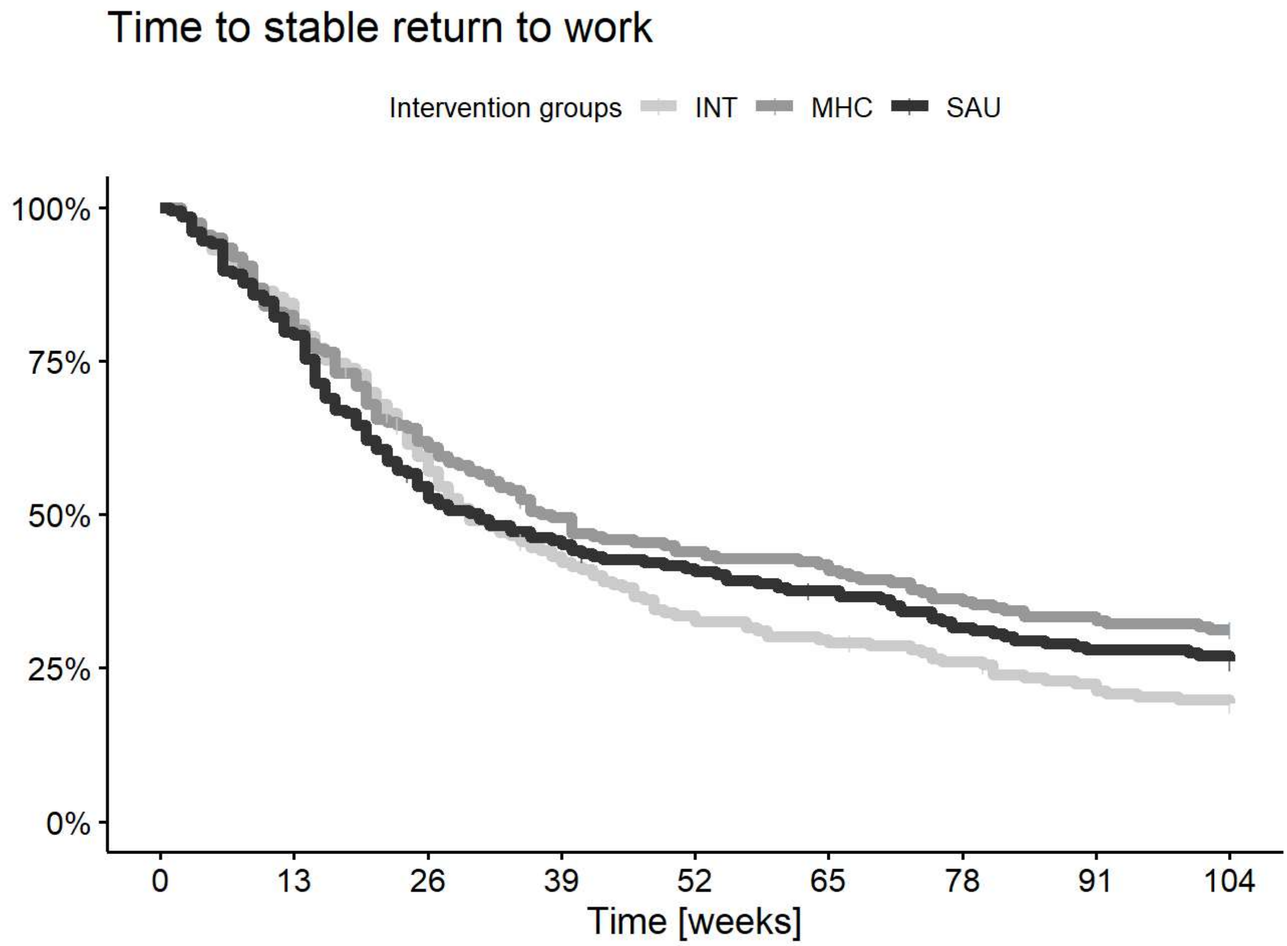

Analyses regarding: >first<

Proportion over time-curve at 24-month follow-up regarding: first<

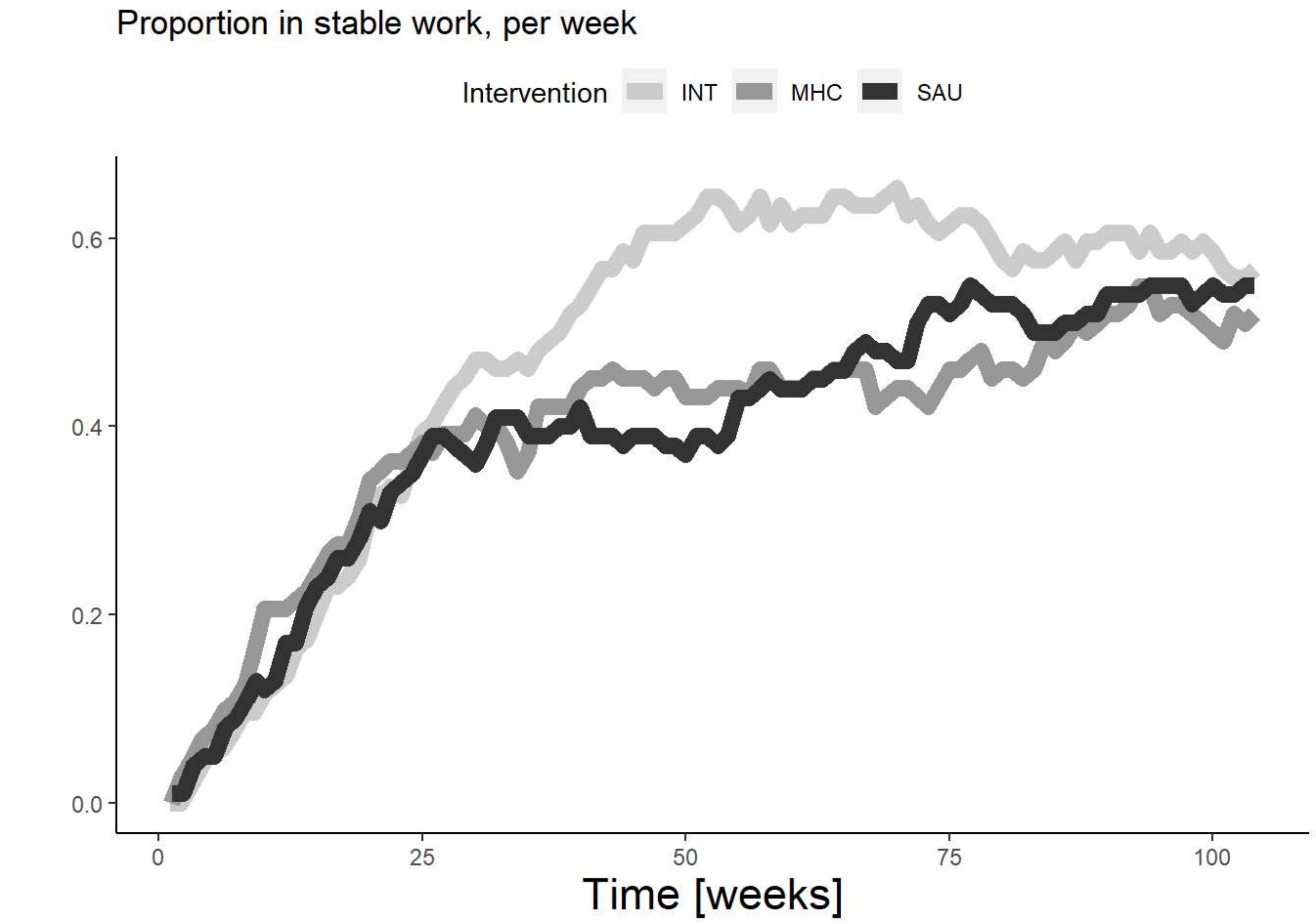

Vocational outcomes at 24-month follow-up from subgroup >first< in RCT1

	Group values			SAU-MHC				SAU-INT				MHC-INT			
	**INT**	**MHC**	**SAU**	**Est.**	**p**	**low CI**	**high CI**	**Est.**	**p**	**low CI**	**high CI**	**Est.**	**p**	**low CI**	**high CI**
RTW, FU24	28.0	36.0	32.0	1.12	0.49	0.75	1.68	0.86	0.35	0.59	1.27	0.78	0.13	0.53	1.16
PROP, FU24	60.2	52.5	56.1	1.27	0.42	0.62	2.62	0.85	0.59	0.42	1.74	0.69	0.21	0.34	1.41
WEEKS, FU24	52.8	42.1	42.2	1.04	0.70	0.79	1.37	0.81	*0.043	0.64	1.04	0.79	*0.0239	0.61	1.02

Kaplan Meier-curve at 24-month follow-up regarding: first

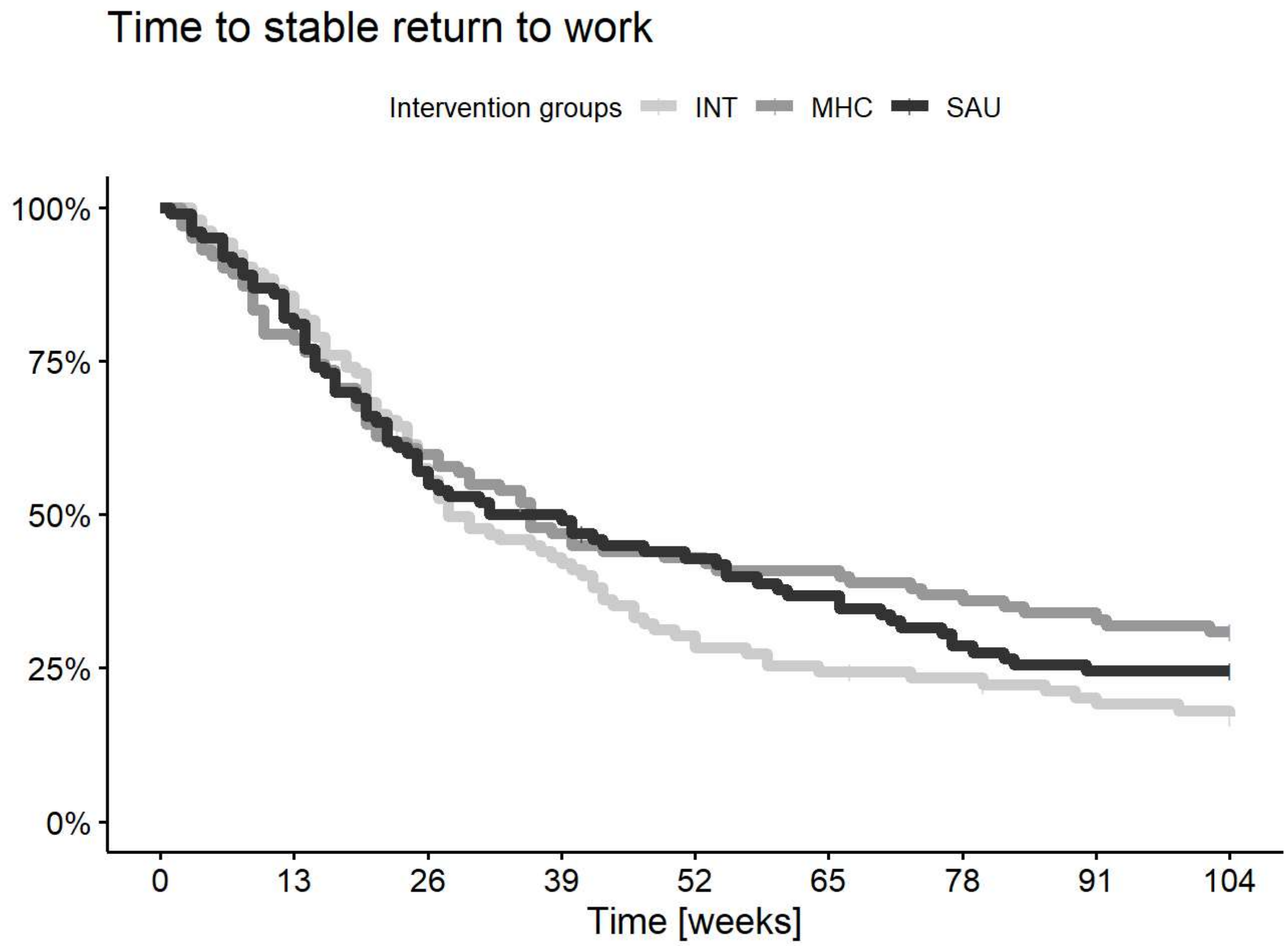

Self-reported outcomes at 24-month follow-up regarding: first

	Group values						SAU-MHC				SAU-INT				MHC-INT			
	INT	(SD)	MHC	(SD)	SAU	(SD)	Est.	p	low CI	high CI	Est.	p	low CI	high CI	Est.	p	low CI	high CI
BAI [FU24]	12.49	(10.07)	11.06	(7.48)	12.62	(9.1)	1.51	0.25	-1.66	4.68	0.6	0.66	-2.69	3.88	-1.08	0.41	-4.21	2.06
BDI [FU24]	11.42	(10.84)	8.57	(8.3)	11.85	(10.27)	2.51	0.1	-1.17	6.2	1.02	0.51	-2.7	4.75	-1.57	0.27	-5.02	1.88
PSS [FU24]	15.86	(8.57)	14.08	(6.49)	15.97	(7.45)	1.44	0.19	-1.19	4.07	0.41	0.72	-2.31	3.14	-1.12	0.29	-3.65	1.4
KES [FU24]	59.14	(20.83)	53.24	(16.61)	61.35	(18.62)	5.58	*0.025	-0.38	11.54	1.33	0.61	-4.88	7.55	-4.57	0.06	-10.46	1.32
DSQ-som. [FU24]	10.18	(7.17)	9.17	(6.06)	10.18	(6.64)	0.7	0.47	-1.64	3.03	0.08	0.93	-2.23	2.39	-0.73	0.45	-3.05	1.6
DSQ-distr. [FU24]	12.11	(8.36)	10.92	(7.19)	13.47	(8.82)	1.63	0.16	-1.14	4.39	0.96	0.41	-1.85	3.76	-0.77	0.47	-3.33	1.79
DSQ-anx. [FU24]	3.19	(3.83)	3	(3.26)	3.96	(4.56)	0.71	0.31	-0.98	2.41	0.5	0.5	-1.28	2.28	-0.3	0.66	-1.94	1.34
DSQ-depr. [FU24]	1.54	(2.45)	1.1	(2.14)	1.68	(2.7)	0.44	0.3	-0.57	1.45	0.15	0.72	-0.87	1.17	-0.32	0.4	-1.24	0.59
WSAS [FU24]	11.65	(10.29)	9.88	(9.13)	11.76	(10.23)	1.18	0.43	-2.45	4.82	0.36	0.81	-3.18	3.9	-0.93	0.52	-4.39	2.52
SPS [FU24]	18.76	(4.09)	18.35	(3.66)	19.37	(3.27)	0.78	0.31	-1.03	2.59	1.04	0.12	-0.56	2.63	0.31	0.68	-1.47	2.08
IPQ [FU24]	17.25	(4.56)	16.94	(4.11)	16.91	(3.65)	0.09	0.89	-1.44	1.61	-0.26	0.67	-1.74	1.22	-0.27	0.66	-1.77	1.23
GSS [FU24]	27.96	(6.52)	28.87	(6.28)	28.07	(6.96)	-0.63	0.55	-3.15	1.89	0.01	0.99	-2.46	2.48	0.7	0.48	-1.65	3.04
QoLs [FU24]	73.26	(15.68)	75.97	(14.51)	71.56	(15.16)	-2.84	0.19	-8.03	2.36	-0.9	0.68	-6.1	4.31	2.07	0.31	-2.84	6.98
EQ5 [FU24]	0.79	(0.19)	0.82	(0.13)	0.8	(0.15)	-0.01	0.55	-0.07	0.04	0.01	0.77	-0.05	0.06	0.02	0.27	-0.03	0.08

Analyses regarding: >last<

Proportion over time-curve at 24-month follow-up regarding: last<

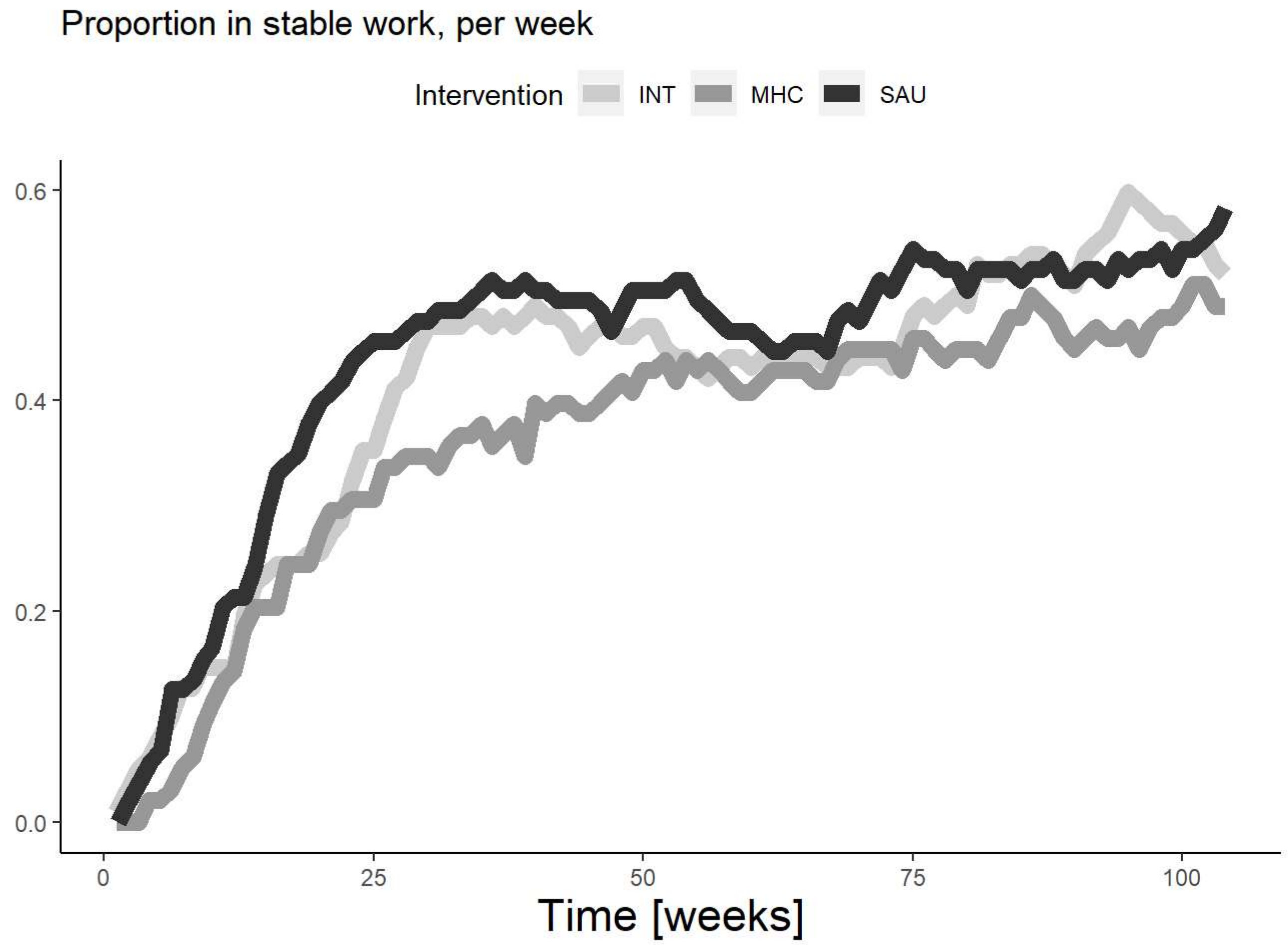

Vocational outcomes at 24-month follow-up from subgroup >last< in RCT1

	Group values			SAU-MHC				SAU-INT				MHC-INT			
	INT	MHC	SAU	Est.	p	low CI	high CI	Est.	p	low CI	high CI	Est.	p	low CI	high CI
RTW, FU24	32	40	27	1.24	0.2	0.82	1.89	1	0.98	0.67	1.5	0.79	0.17	0.53	1.19
PROP, FU24	53.5	49.5	58.8	1.45	0.21	0.71	2.92	1.3	0.38	0.64	2.64	0.86	0.6	0.42	1.76
WEEKS, FU24	44.3	38.6	46.6	1.21	0.11	0.9	1.62	1.07	0.49	0.84	1.38	0.88	0.29	0.67	1.17

Kaplan Meier-curve at 24-month follow-up regarding: last

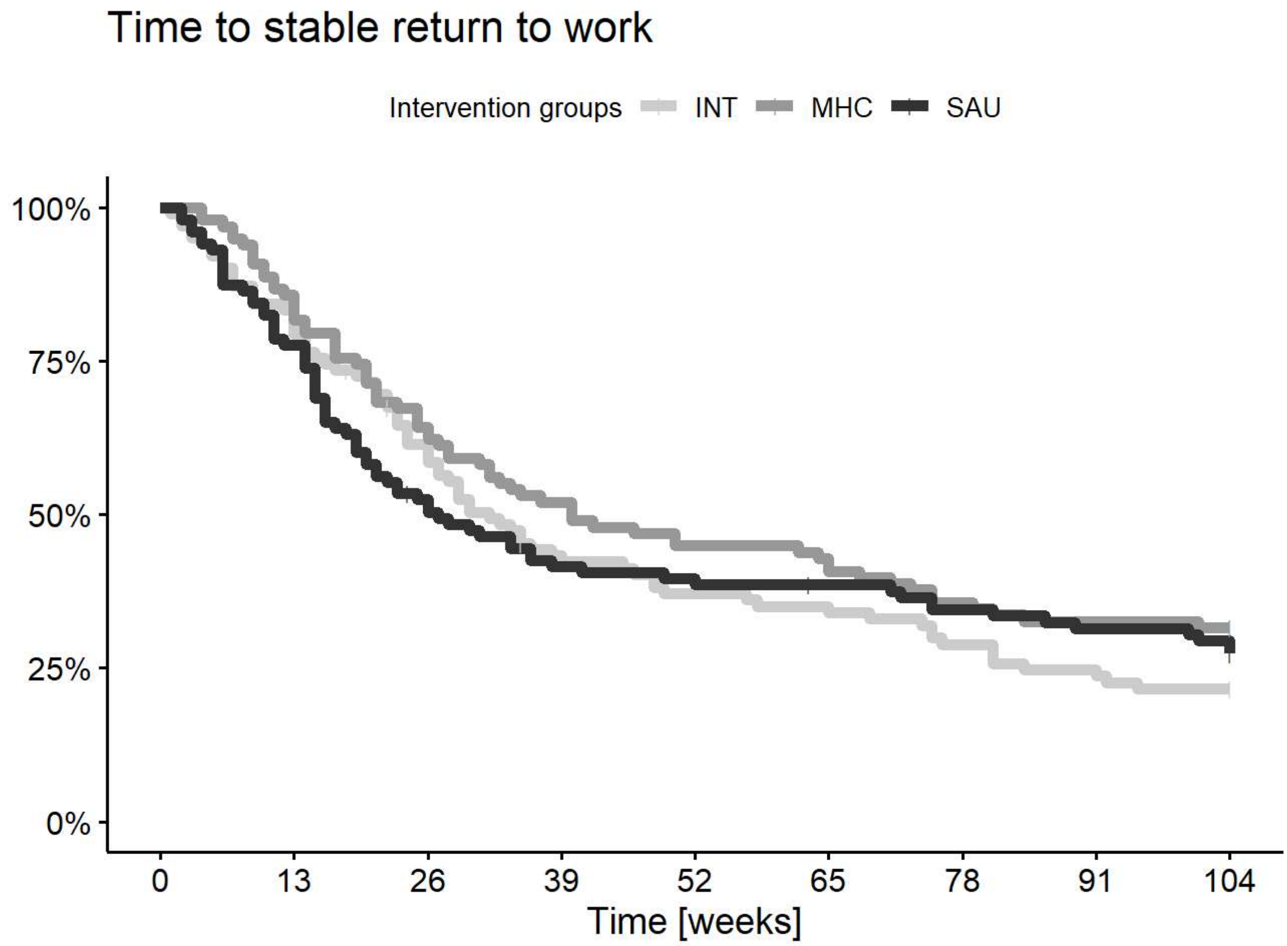

Self-reported outcomes at 24-month follow-up regarding: last

	Group values						SAU-MHC				SAU-INT				MHC-INT			
	INT	(SD)	MHC	(SD)	SAU	(SD)	Est.	p	low CI	high CI	Est.	p	low CI	high CI	Est.	p	low CI	high CI
BAI [FU24]	10.99	(7.89)	13.47	(10.59)	10.78	(9.22)	-1.15	0.42	-4.6	2.3	0.94	0.48	-2.27	4.15	2.19	0.09	-0.95	5.32
BDI [FU24]	11	(11.81)	13.91	(12.43)	9.95	(10.49)	-2.86	0.07	-6.59	0.87	-0.53	0.72	-4.17	3.11	2.29	0.12	-1.27	5.85
PSS [FU24]	15.25	(8.22)	17.12	(7.53)	13.98	(7.66)	-1.83	0.09	-4.44	0.78	-0.29	0.79	-2.9	2.33	1.57	0.14	-0.95	4.1
KES [FU24]	56.39	(18.67)	63.62	(20.78)	55.21	(17.85)	-6.19	**0.0139	-12.22	-0.16	-0.57	0.82	-6.49	5.34	5.69	*0.0232	-0.31	11.69
DSQ-som. [FU24]	8.6	(6.69)	9.66	(6.35)	9.31	(6.29)	-0.19	0.85	-2.52	2.15	0.83	0.39	-1.49	3.16	1.07	0.26	-1.22	3.35
DSQ-distr. [FU24]	12.31	(8.35)	15.04	(8.67)	11.07	(8.11)	-3.02	**0.0074	-5.71	-0.32	-0.83	0.46	-3.54	1.88	2.16	0.05	-0.53	4.84
DSQ-anx. [FU24]	3.7	(4.85)	5.71	(6.18)	3.02	(4.52)	-1.52	0.06	-3.47	0.43	-0.1	0.9	-1.86	1.67	1.49	*0.0484	-0.32	3.3
DSQ-depr. [FU24]	1.69	(3.02)	2.43	(3.17)	1.76	(3.05)	-0.54	0.23	-1.62	0.54	0.03	0.94	-1.02	1.09	0.57	0.18	-0.45	1.59
WSAS [FU24]	11.34	(10.92)	13.89	(11.14)	10.64	(9.48)	-2.42	0.11	-6.01	1.18	-0.12	0.93	-3.53	3.29	2.37	0.1	-1.11	5.85
SPS [FU24]	18.46	(4.51)	20.02	(3.74)	18.8	(3.51)	0.78	0.31	-1.03	2.59	1.04	0.12	-0.56	2.63	0.31	0.68	-1.47	2.08
IPQ [FU24]	17.74	(4.13)	16.62	(4.12)	16.97	(4.58)	0.31	0.62	-1.2	1.81	-0.55	0.36	-2	0.89	-0.87	0.14	-2.29	0.56
GSS [FU24]	28.32	(7.21)	27.24	(7.99)	30.21	(6.94)	1.72	0.1	-0.78	4.23	0.66	0.52	-1.78	3.09	-1.08	0.3	-3.57	1.42
QoLs [FU24]	72.88	(17.8)	68.93	(18.74)	74.28	(15.76)	4.06	0.07	-1.39	9.51	1.5	0.49	-3.69	6.69	-2.36	0.29	-7.75	3.03
EQ5 [FU24]	0.81	(0.17)	0.77	(0.19)	0.81	(0.14)	0.03	0.16	-0.02	0.09	0	0.83	-0.05	0.06	-0.03	0.27	-0.09	0.03

Analyses regarding: >inttemp<

Proportion over time-curve at 24-month follow-up regarding: inttemp<

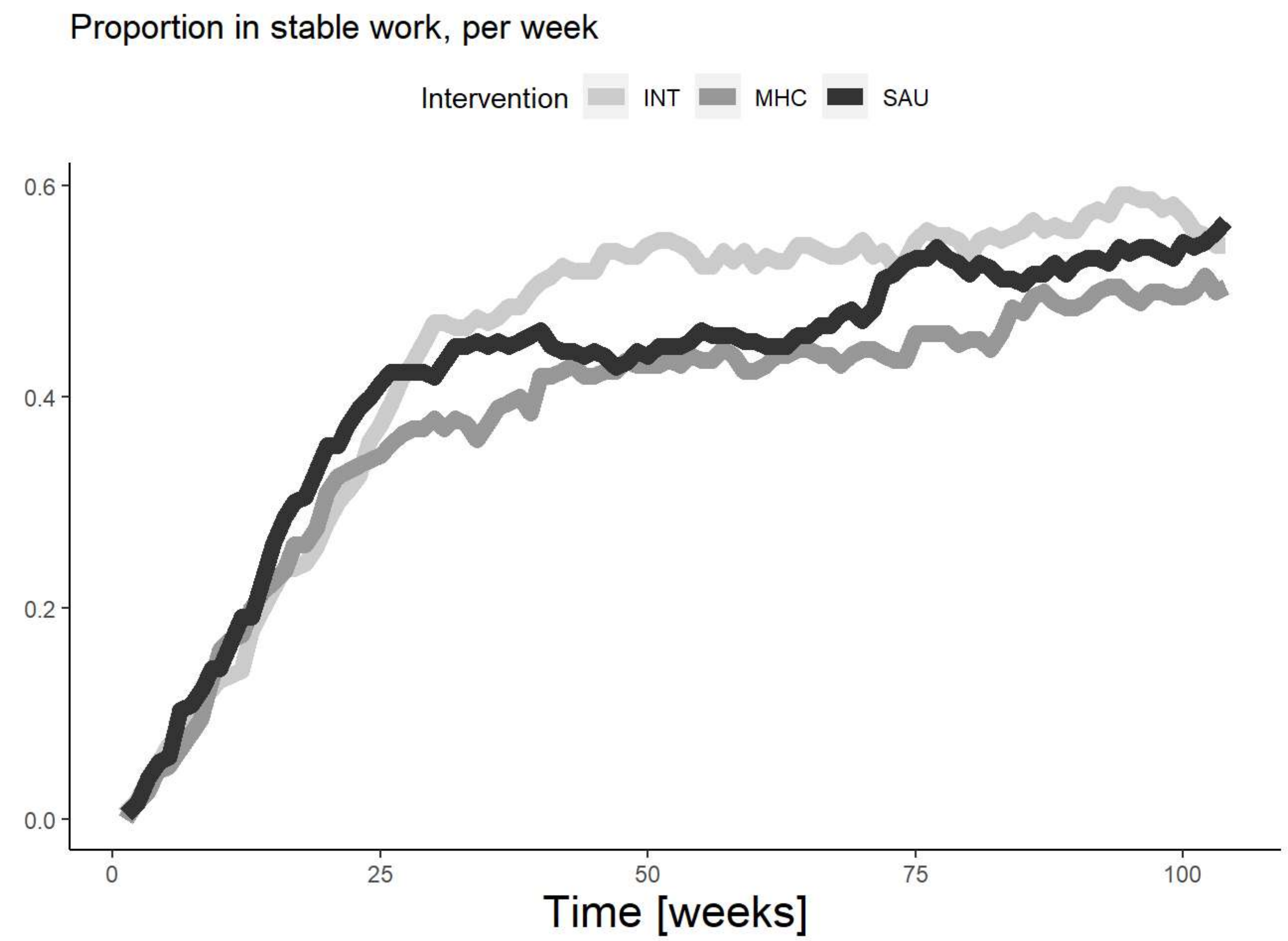

Vocational outcomes at 24-month follow-up from subgroup >inttemp< in RCT1

	Group values			SAU-MHC					SAU-INT					MHC-INT				
	INT	MHC	SAU	Est.	p	low CI	:	high CI	Est.	p	low CI	:	high CI	Est.	p	low CI	:	high CI
RTW, FU24	30.0	37.0	31.0	1.12	0.48	0.75		1.68	0.88	0.43	0.60		1.29	0.78	0.12	0.53		1.15
RTW, FU24:temphalflast				1.08	0.75				1.11	0.64				1.04	0.87			
PROP, FU24	56.9	51.0	57.5	1.23	0.48	0.61		2.49	0.85	0.59	0.42		1.73	0.70	0.22	0.35		1.41
PROP, FU24:temphalflast				0.14	0.74				0.40	0.33				0.23	0.58			
WEEKS, FU24	48.5	40.4	44.5	1.03	0.82	0.78		1.35	0.81	*0.0422	0.63		1.04	0.79	*0.0272	0.61		1.02
WEEKS, FU24:temphalflast				0.35	0.08				0.32	0.09				-0.05	0.79			

Kaplan Meier-curve at 24-month follow-up regarding: inttemp

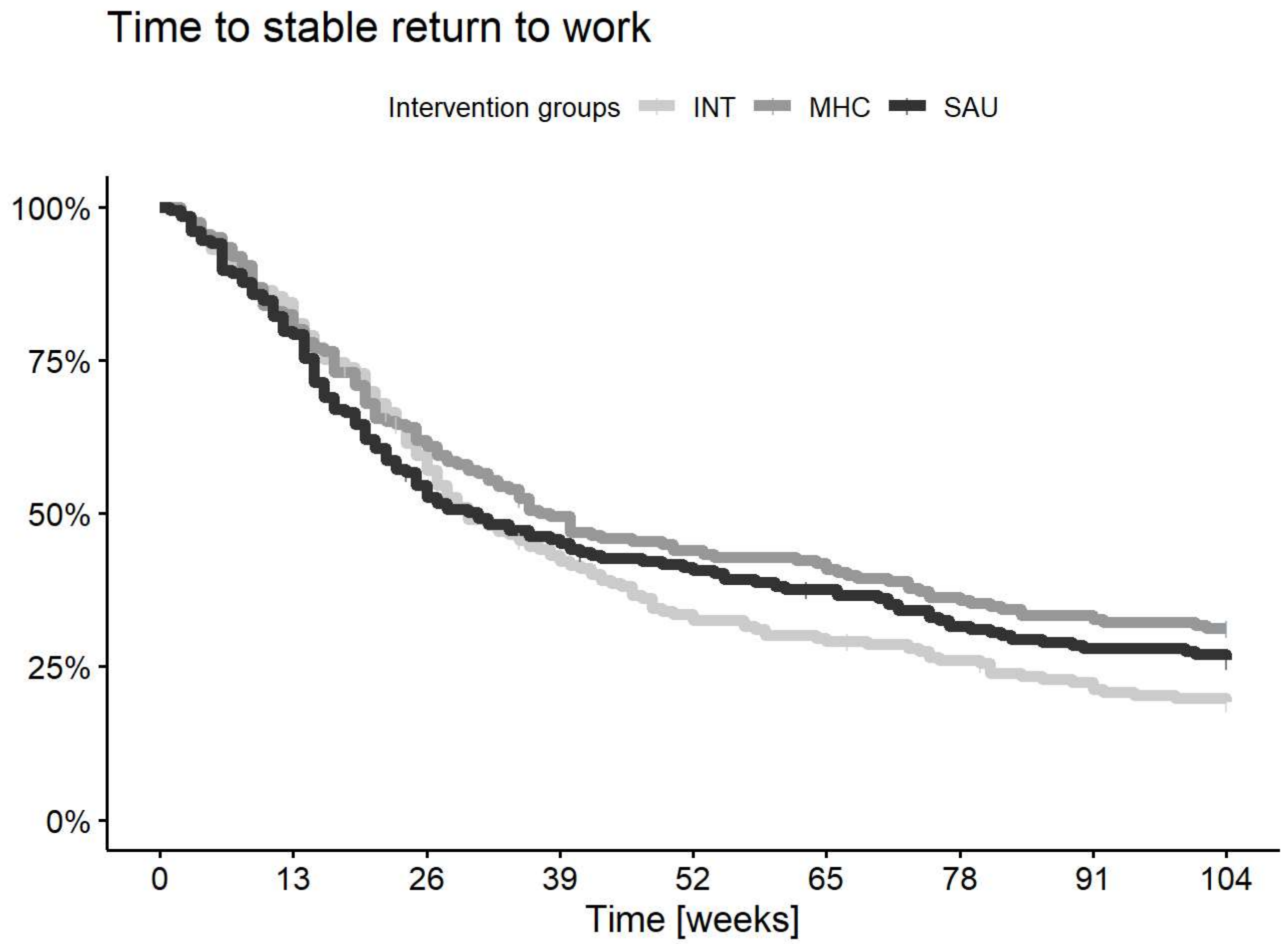
Time to stable return to work
Intervention groups INT MHC SAU
100%
75%
50%
25%
0%
0 13 26 39 52 65 78 91 104
Time [weeks]

Supplement 7: Sensitivity analyses

The following pages contains the results from the sensitivity analyses of the IBBIS Randomized Trial for anxiety and depression

Supplement: Sensitivity analyses

Abbrev.: IBBIS: Integreret Behandlings- og BeskaeftigelsesIndsats til Sygedagpengemodtagere (English translation: Integrated Health Care and Vocational Rehabilitation for Sick Leave Benefit Recipients); HR: Hazard Ratio; INT: Integrated intervention; MHC: Mental health care; RCT: Randomized controlled trial; RTW: Return to work; SAU: Service as usual; OR: Odds Ratio; RR: Relative risk; FUn: n-month follow-up

Table legends: “Est.”: Estimate “p”: p-value “low/high CI”: Low vs. high bounds of the 98.3% Confidence Interval of the estimate.

Legend: *: 0,05 > p > 0,016667 | **: p < 0,016667

In this document the term RCT1 refers to this RCT

This report chapter systematically displays all results from the IBBIS RCT trial covering diagnoses of anxiety and depression.

Sensitivity analysis

All sensitivity analysis are performed using the entire group (consult the statistical analysis plan for details)

Sensitivity scenario: >best<

Proportion over time-curve at 24-month follow-up regarding scenario: >best<

Vocational outcomes at 24-month follow-up from subgroup >all< in RCT1

	Group values			SAU-MHC				SAU-INT				MHC-INT			
	INT	MHC	SAU	Est.	p	low CI	high CI	Est.	p	low CI	high CI	Est.	p	low CI	high CI
RTW, FU24	29.0	36.0	27.0	1.17	0.20	0.88	1.55	0.93	0.52	0.71	1.22	0.79	*0.0444	0.60	1.05
PROP, FU24	58.3	52.9	58.9	1.32	0.18	0.81	2.17	1.05	0.83	0.64	1.72	0.79	0.24	0.48	1.29
WEEKS, FU24	50.4	42.9	46.5	1.10	0.23	0.91	1.35	0.93	0.31	0.78	1.11	0.84	*0.024	0.69	1.01

Kaplan Meier-curve at 24-month follow-up regarding scenario: best

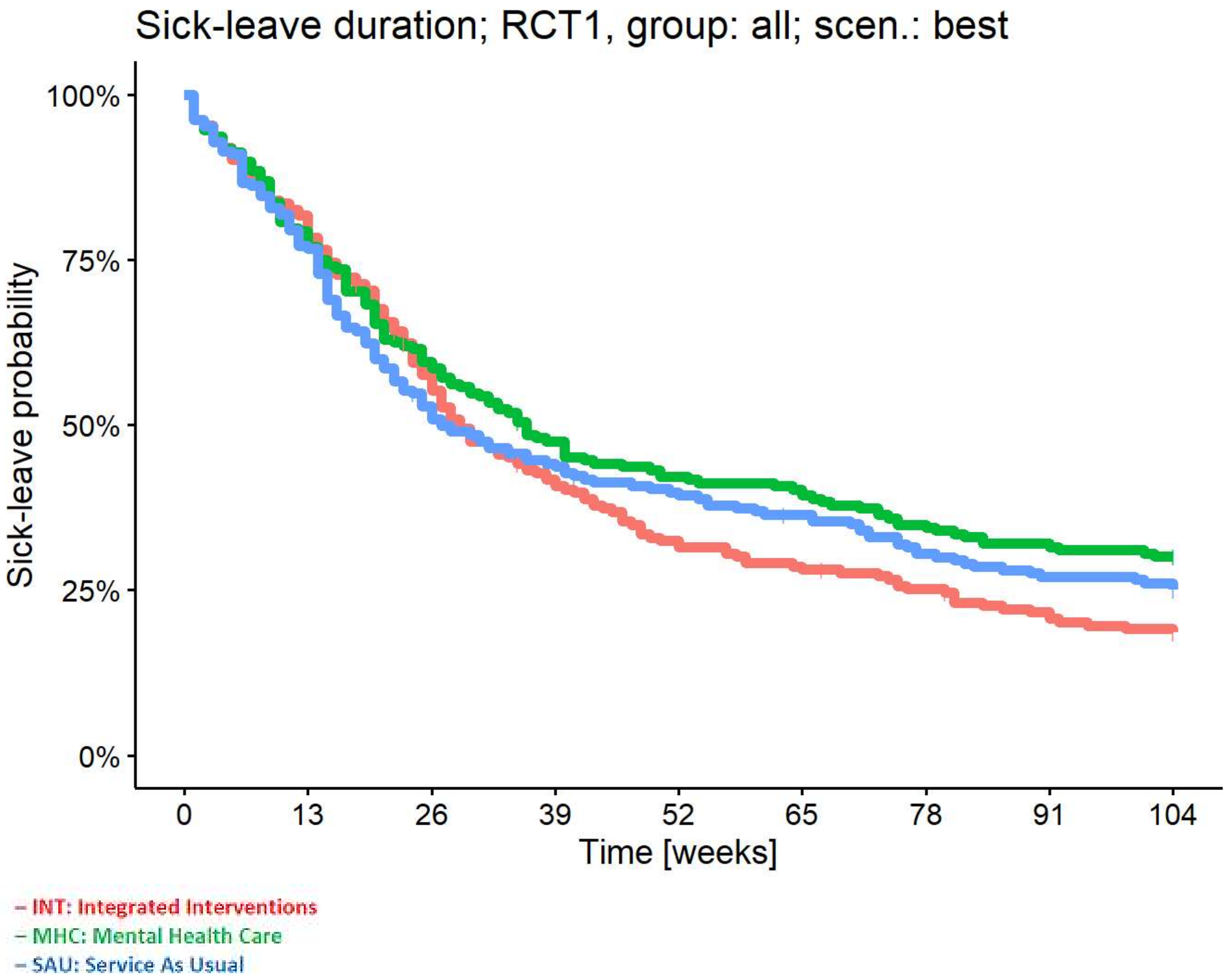

Proportion over time-curve at 24-month follow-up regarding scenario: >best<

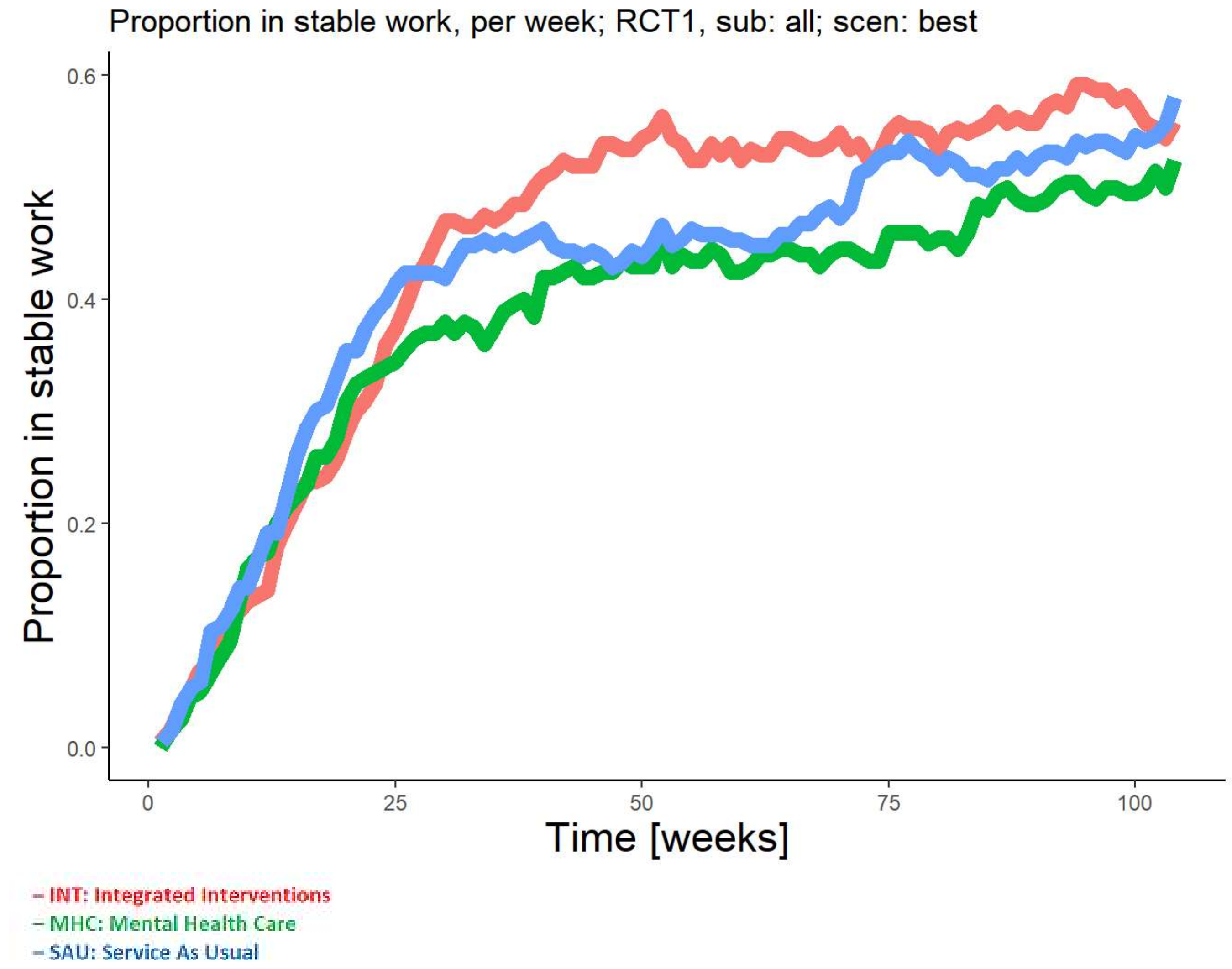

Self-reported outcomes at 24-month follow-up regarding scenario: best (RCT1)

	Group values						SAU-MHC				SAU-INT				MHC-INT			
	INT	(SD)	MHC	(SD)	SAU	(SD)	Est.	p	low CI	high CI	Est.	p	low CI	high CI	Est.	p	low CI	high CI
BAI [FU24]	6.13	(11.2)	6.25	(11.55)	3.97	(11.44)	-2.4	*0.0259	-4.97	0.18	-2.31	*0.027	-4.82	0.19	0.12	0.91	-2.35	2.58
BDI [FU24]	4.53	(13.73)	4.4	(13.68)	1.91	(13.71)	-2.62	*0.0319	-5.55	0.3	-3.03	**0.0113	-5.88	-0.17	-0.38	0.74	-3.19	2.42
PSS [FU24]	10.72	(10.07)	10.57	(9.53)	8.56	(9.75)	-2.18	**0.009	-4.17	-0.18	-2.36	**0.0049	-4.38	-0.35	-0.19	0.82	-2.16	1.78
KES [FU24]	45.34	(24.13)	45.78	(24.37)	41.44	(24.22)	-4.61	*0.0327	-9.78	0.56	-4.47	*0.0345	-9.54	0.59	0.14	0.95	-4.87	5.15
DSQ-som. [FU24]	5.3	(8.32)	5.11	(7.91)	3.97	(8.34)	-1.25	0.11	-3.1	0.61	-1.46	0.06	-3.31	0.39	-0.19	0.8	-2.02	1.63
DSQ-distr. [FU24]	6.96	(10.23)	7.43	(10.57)	5.2	(10.58)	-2.31	**0.014	-4.57	-0.06	-2.02	*0.0285	-4.22	0.19	0.29	0.75	-1.89	2.47
DSQ-anx. [FU24]	0.56	(5.52)	1.1	(6.3)	-0.47	(5.69)	-1.59	**0.0083	-3.03	-0.15	-1.1	0.06	-2.47	0.28	0.51	0.38	-0.87	1.89
DSQ-depr. [FU24]	-0.13	(3.39)	-0.07	(3.5)	-0.68	(3.57)	-0.65	0.06	-1.49	0.19	-0.63	0.07	-1.45	0.2	0.02	0.96	-0.78	0.81
WSAS [FU24]	5.18	(12.94)	5.23	(12.95)	2.61	(12.71)	-2.78	*0.0171	-5.58	0.01	-2.82	**0.0123	-5.51	-0.12	-0.02	0.98	-2.74	2.69
SPS [FU24]	22.49	(5.04)	23.01	(4.51)	23.43	(4.33)	0.33	0.41	-0.63	1.28	0.57	0.2	-0.49	1.63	0.18	0.68	-0.9	1.27
IPQ [FU24]	14.67	(5.49)	14.05	(5.1)	13.23	(5.12)	-0.7	0.13	-1.79	0.4	-1.35	**0.0028	-2.43	-0.27	-0.64	0.16	-1.73	0.46
GSS [FU24]	32.88	(8.78)	32.98	(9)	34.96	(8.6)	2.08	**0.0121	0.1	4.06	2.27	**0.0049	0.34	4.21	0.19	0.82	-1.73	2.1
QoLs [FU24]	62.32	(21.07)	61.38	(20.89)	57.86	(20.17)	-2.74	0.13	-7.12	1.63	-3.93	*0.0285	-8.23	0.37	-1.14	0.52	-5.45	3.16
EQ5 [FU24]	0.91	(0.21)	0.9	(0.21)	0.95	(0.2)	0.04	*0.0208	0	0.09	0.04	*0.0184	0	0.09	0	1	-0.04	0.05

Sensitivity scenario: >worst<

Proportion over time-curve at 24-month follow-up regarding scenario: >worst<

Vocational outcomes at 24-month follow-up from subgroup >all< in RCT1

	Group values			SAU-MHC				SAU-INT				MHC-INT			
	**INT**	**MHC**	**SAU**	**Est.**	**p**	**low CI**	**:high CI**	**Est.**	**p**	**low CI**	**:high CI**	**Est.**	**p**	**low CI**	**:high CI**
RTW, FU24	32.0	40.0	32.0	1.17	0.20	0.88	1.55	0.93	0.52	0.71	1.22	0.79	*0.0444	0.60	1.05
PROP, FU24	54.9	49.0	55.6	1.32	0.18	0.81	2.17	1.05	0.83	0.64	1.72	0.79	0.24	0.48	1.29
WEEKS, FU24	46.9	38.9	43.0	1.10	0.23	0.91	1.35	0.93	0.31	0.78	1.11	0.84	*0.024	0.69	1.01

Kaplan Meier-curve at 24-month follow-up regarding scenario: worst

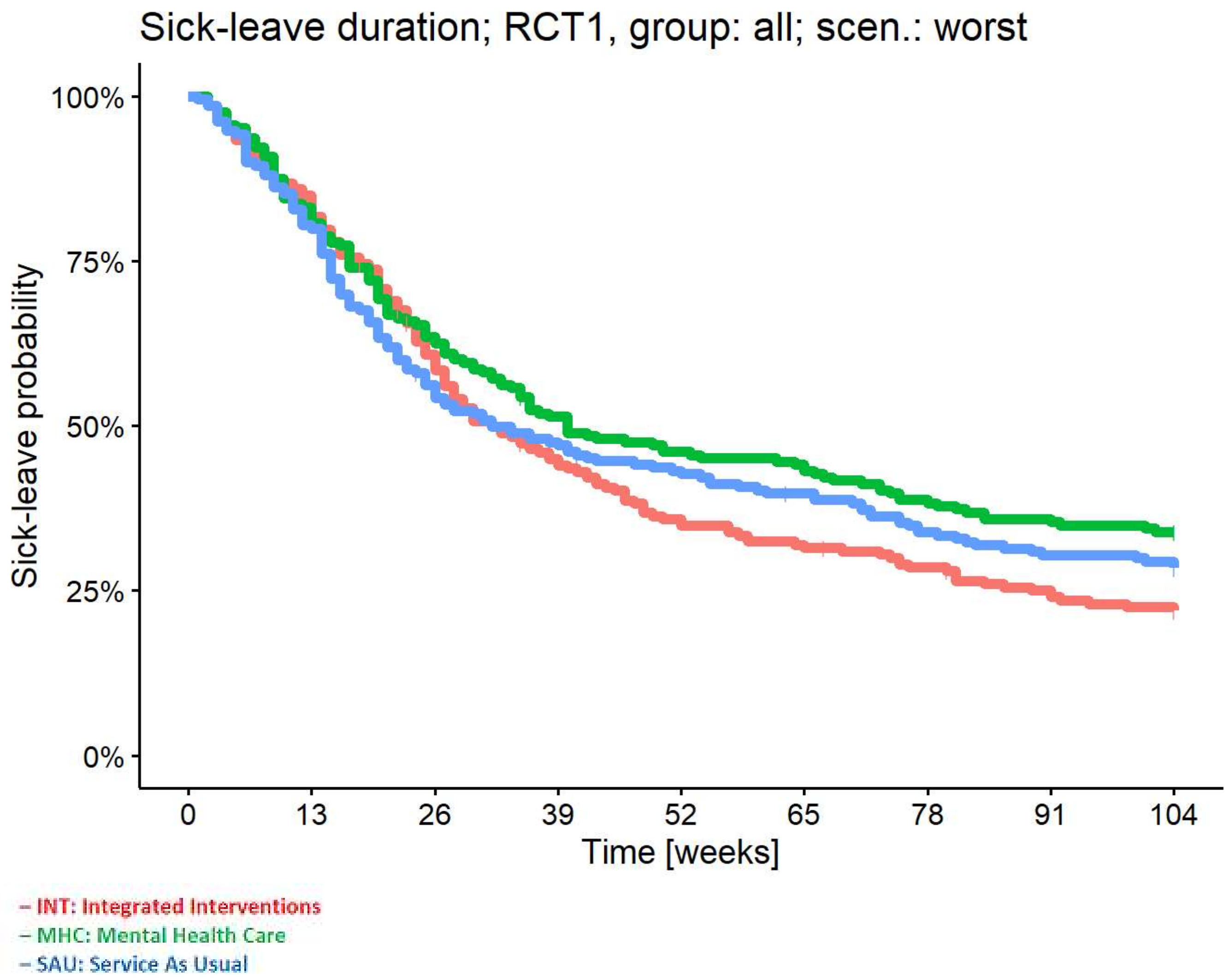

Proportion over time-curve at 24-month follow-up regarding scenario: >worst<

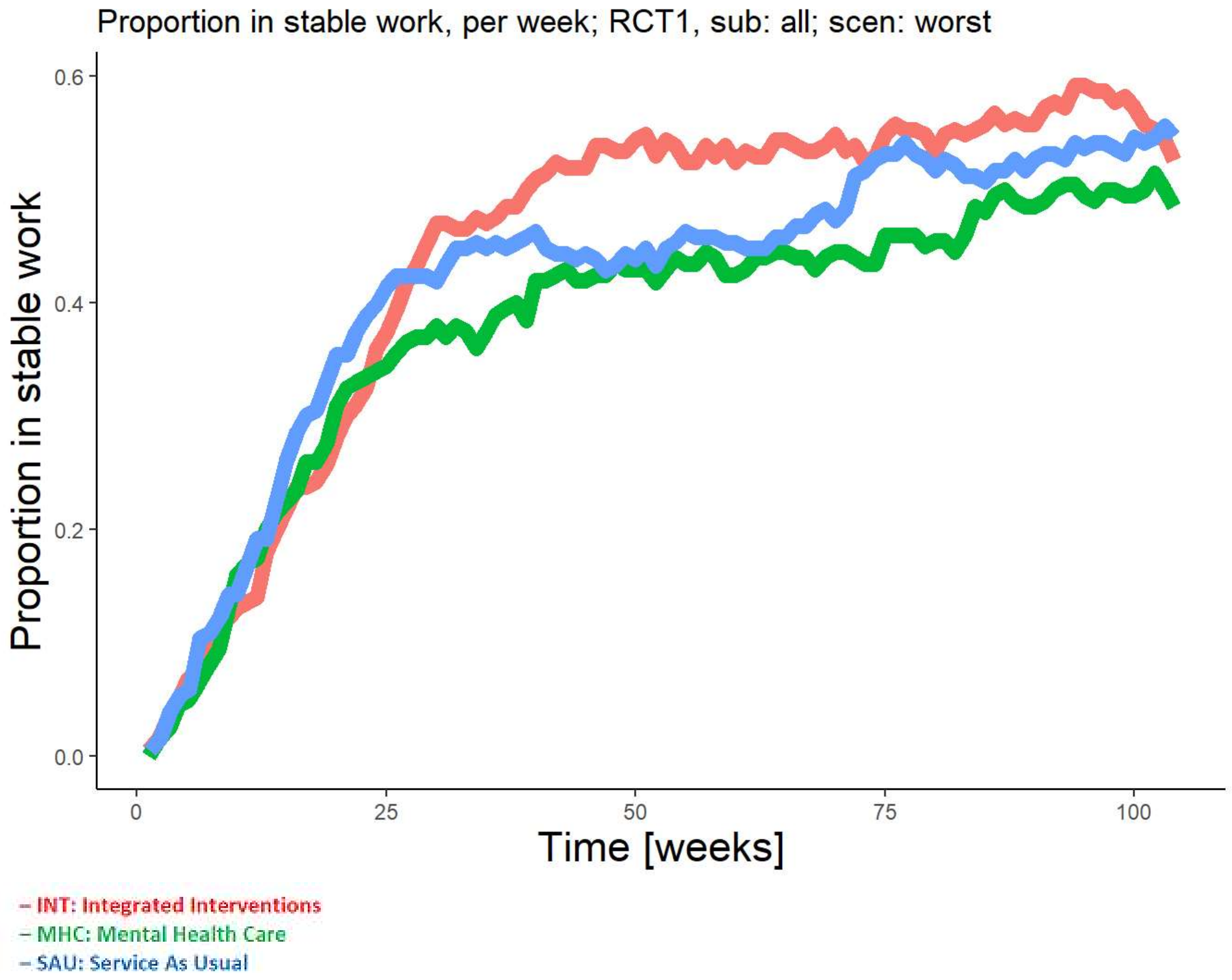

Self-reported outcomes at 24-month follow-up regarding scenario: worst (RCT1)

	Group values						SAU-MHC				SAU-INT				MHC-INT			
	INT	(SD)	MHC	(SD)	SAU	(SD)	Est.	p	low CI	high CI	Est.	p	low CI	high CI	Est.	p	low CI	high CI
BAI [FU24]	17.29	(11.37)	17.86	(11.38)	19.82	(11.42)	1.56	0.14	-1	4.13	2.32	*0.0259	-0.17	4.81	0.76	0.46	-1.73	3.25
BDI [FU24]	17.75	(13.86)	18.36	(13.58)	20.76	(13.64)	1.92	0.11	-0.96	4.8	2.54	*0.0319	-0.3	5.38	0.61	0.6	-2.19	3.4
PSS [FU24]	20.32	(10.04)	20.55	(9.45)	22.19	(9.9)	1.23	0.14	-0.76	3.21	1.62	0.05	-0.38	3.62	0.37	0.66	-1.61	2.34
KES [FU24]	70.14	(24.68)	71.18	(24.17)	75.85	(23.7)	3.8	0.07	-1.27	8.87	5.01	*0.0169	-0.01	10.04	1.18	0.58	-3.86	6.21
DSQ-som. [FU24]	13.43	(8.42)	13.56	(8.06)	15.47	(7.99)	1.6	*0.039	-0.26	3.45	1.88	**0.0138	0.05	3.7	0.28	0.71	-1.54	2.11
DSQ-distr. [FU24]	17.51	(10.57)	18.4	(10.19)	19.9	(10.6)	1.15	0.22	-1.07	3.37	2.08	*0.024	-0.13	4.29	0.91	0.31	-1.25	3.06
DSQ-anx. [FU24]	6.47	(5.79)	7.24	(5.9)	7.76	(5.88)	0.35	0.56	-1.07	1.77	1.2	*0.0374	-0.18	2.58	0.85	0.14	-0.53	2.23
DSQ-depr. [FU24]	3.39	(3.48)	3.58	(3.45)	4.21	(3.5)	0.5	0.14	-0.31	1.32	0.73	*0.0301	-0.08	1.54	0.22	0.5	-0.57	1.01
WSAS [FU24]	17.79	(13.06)	18.35	(12.77)	20.51	(12.79)	1.69	0.14	-1.06	4.44	2.42	*0.0309	-0.27	5.11	0.71	0.53	-1.98	3.39
SPS [FU24]	15.09	(4.76)	15.21	(4.75)	14.61	(4.43)	-0.29	0.48	-1.27	0.69	-0.04	0.93	-1.06	0.99	0.31	0.48	-0.74	1.37
IPQ [FU24]	20.13	(5.16)	19.72	(5.32)	20.69	(5.3)	0.96	*0.0434	-0.18	2.1	0.62	0.17	-0.48	1.73	-0.33	0.47	-1.41	0.76
GSS [FU24]	23.72	(8.67)	23.46	(8.8)	22.45	(9.03)	-0.69	0.39	-2.65	1.26	-1.04	0.19	-2.97	0.88	-0.34	0.67	-2.24	1.57
QoLs [FU24]	83.84	(20.72)	83.74	(21.08)	87.29	(20.41)	3.78	*0.0428	-0.69	8.24	3.87	*0.0338	-0.5	8.24	0.09	0.96	-4.24	4.42
EQ5 [FU24]	0.7	(0.21)	0.69	(0.2)	0.66	(0.2)	-0.03	0.14	-0.07	0.02	-0.04	*0.0488	-0.08	0.01	-0.01	0.64	-0.05	0.04
